# BCL::Score—Knowledge Based Energy Potentials for Ranking Protein Models Represented by Idealized Secondary Structure Elements

**DOI:** 10.1371/journal.pone.0049242

**Published:** 2012-11-16

**Authors:** Nils Woetzel, Mert Karakaş, Rene Staritzbichler, Ralf Müller, Brian E. Weiner, Jens Meiler

**Affiliations:** Department of Chemistry, Vanderbilt University, Nashville, Tennessee, United States of America; University Of Oxford, United Kingdom

## Abstract

The topology of most experimentally determined protein domains is defined by the relative arrangement of secondary structure elements, i.e. α-helices and β-strands, which make up 50–70% of the sequence. Pairing of β-strands defines the topology of β-sheets. The packing of side chains between α-helices and β-sheets defines the majority of the protein core. Often, limited experimental datasets restrain the position of secondary structure elements while lacking detail with respect to loop or side chain conformation. At the same time the regular structure and reduced flexibility of secondary structure elements make these interactions more predictable when compared to flexible loops and side chains. To determine the topology of the protein in such settings, we introduce a tailored knowledge-based energy function that evaluates arrangement of secondary structure elements only. Based on the amino acid C_β_ atom coordinates within secondary structure elements, potentials for amino acid pair distance, amino acid environment, secondary structure element packing, β-strand pairing, loop length, radius of gyration, contact order and secondary structure prediction agreement are defined. Separate penalty functions exclude conformations with clashes between amino acids or secondary structure elements and loops that cannot be closed. Each individual term discriminates for native-like protein structures. The composite potential significantly enriches for native-like models in three different databases of 10,000–12,000 protein models in 80–94% of the cases. The corresponding application, “BCL::ScoreProtein,” is available at www.meilerlab.org.

## Introduction

Many protein structures have been determined using experimental techniques such as X-ray crystallography and Nuclear Magnetic Resonance (NMR) spectroscopy. Of the approximately 69,000 protein structures deposited in the Protein Data Bank (PDB) as of August 2011, X-ray crystallography [1] contributed 88% and nuclear magnetic resonance (NMR) [2] contributed almost all of the remaining 12% [3]. Although the number of experimentally determined protein structures grows, challenges still exist. Membrane proteins are hard to express, crystallize and are usually too large to be studied by NMR [4]. Some proteins evade atomic detail structure determination in isolation and adopt their biologically relevant structure only in the context of a complete biomolecular assembly, e.g. a virus or macromolecular machine [5].

The biological importance of these proteins justifies large efforts to collect limited experimental datasets that describe their structure. Often these data restrain the topology of the protein, i.e. the relative placement of secondary structure elements (SSEs). For example, electron density maps of medium resolution (4–10 Å) obtained by X-ray crystallography or cryo-Electron Microscopy (cryo-EM) [6]–[9] display the location of secondary structure elements but omit loop regions and side chains. Small-Angle X-ray Scattering (SAXS) and Small-Angle Neutron Scattering (SANS) display the overall shape of the protein topology [5], [10]. NMR spectroscopy of large and/or membrane proteins often yields distance and orientation restraints for atoms in the backbone of SSEs which are easier to label, assign, and interpret. Site-Directed Spin Labeling Electron Paramagnetic Resonance (SDSL-EPR) spectroscopy is applied to interrogate the relative positioning of SSEs relating the information from the tip of the non-natural and flexible spin label back onto the protein backbone [11], [12]. Lastly, cross-linking experiments interpreted with mass spectrometry yield typically distance restraints that again focus on the relative position of SSEs [13]. To facilitate construction and evaluation of protein structural models from such limited datasets a tailored energy function that only evaluates the relative positioning of SSEs in topologies would be of great value. Ideally, this energy function should predict the free energy of all states an amino acid sequence can access, and the lowest free energy should be associated with the native structure [14]. In principle, the free energy of a protein structure and its native conformation can then be derived with sufficient sampling of the potential energy surface using molecular mechanics force fields (e.g. CHARMM [15] or AMBER [16]). This approach is often computationally prohibitive and sometimes suffers from inaccuracies in the potential energy function. It has been shown that these potentials do not always distinguish native-like from incorrect structures [17].

An alternative approach constructs scoring functions whose global energy minimum coincides with the native conformation for a database of experimentally determined protein structures of different sequence. Early versions of such knowledge-based or statistical potentials were based on contact frequencies [18] and likely exposure states of amino acid types [19]. Since then, a large variety of such potentials have been developed (for a review see [20]), and their applicability to fold recognition (threading) [19] and protein folding [21] was demonstrated. The underlying assumption that the knowledge based distribution of features is a Boltzmann-like distribution can be challenged, e.g. for amino acid pair distances [22]. This is particularly true in protein structure prediction, where the reference state is dependent on the type and density of sampling used [23].

Knowledge based energy functions employ probability theory, and in particular Bayes’ theorem, to circumvent the assumption of a Boltzmann distribution [22]. Shen and Sali derive a Discrete Optimized Protein Energy (DOPE) from a sample of native structures based entirely on probability theory [20]. The potential achieves enrichments between 3 and 9 for the identification of native structures in a set of models [24]. Protein structure prediction with Rosetta uses a low resolution knowledge-based scoring function consisting of an amino acid environment term defined by the burial of an amino acid and an amino acid pair interaction potential defined by all amino acid pair distances [21]. It further includes a secondary structure packing potential for α-helix packing and β-strand pairing in β-sheets. A dot product captures hydrogen bonding in β-strand pairing. This potential uses the loop length connecting two SSEs as an additional dependent variable [25].

The energy function developed herein works off the hypothesis that interactions between SSEs define the core of the protein structure and are the major contributor to the stability of the protein fold, at least for a large fraction of folded proteins. In turn, the majority of stabilizing interactions in the protein structure is present in SSE-only models. Further, it is hypothesized that these stabilizing interactions can be more accurately predicted as flexibility is reduced in the backbone of SSEs when compared to loop regions or amino acid side chains. The expected higher accuracy in placing the SSEs will result in a higher accuracy of the energetic evaluation. As a result, a smoothened energy landscape is expected that can be searched more readily as it is devoid of noise introduced by inaccurately placed loop regions and side chains. The advantages of reduced conformational search space and smoothened energy landscape pair nicely with the above-mentioned settings with limited experimental data, since most experimental restraints relate to SSEs and can thus still be employed in protein folding. It is expected that models constructed and evaluated with this energy function can be readily completed through established protocols for the construction of loops and side chains. For example, loops can be modeled using fragment replacement, cyclic coordinate descent [26], [27], or kinematic loop closure [28]. Side chains are added using dead end elimination or Monte Carlo sampling of rotamer libraries as implemented for example in SCWRL [29] or Rosetta [30].

The present manuscript introduces a comprehensive knowledge-based energy potential for proteins which is based on a simplified representation of the protein including only SSEs, i.e. α-helices and β-strands. The hypothesis is that for the majority of well-structured domains the assembly of the SSEs in three-dimensional space defines the domain topology, i.e. fold. Based on the amino acid C_β_ atom coordinates within the SSEs (H_α2_ atom for Glycine) we define an amino acid pair potential, an amino acid environment potential, a secondary structure element packing potential, a β-strand pairing potential, a loop length potential, a radius of gyration potential, a contact order potential, and a secondary structure formation potential. Separate penalty functions forbid amino acid clashes, SSE clashes and loop distances that cannot be bridged. The overall energy potential is a linearly weighted consensus scoring function. These weights balance the individual terms to evaluate the native-likeliness of the SSE arrangement and the three dimensional placement of the amino acids in the context of the fold. While the scoring function is specialized to evaluate the loop-less protein topology as defined by the SSEs, it can be applied to full chain protein models as well.

## Results and Discussion

### Bayes’ Theorem is Applied to Derive a Comprehensive Knowledge-based Potential

In deriving the present knowledge-based potential we use Bayes’ theorem to estimate the probability of a structure given the sequence. This strategy follows previously described approaches [21], [25] in expanding this probability into a series of terms that desribe certain aspect of the protein structure. This strategy avoids the requirement of a Boltzmann-like distribution of states in the databank:
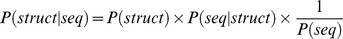
where 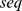 is the amino acid sequence and 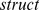 the protein’s three dimensional structure. This approach separates the probability for a given sequence to fold into a certain structure into two terms. The probability of the structure, 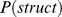, describes the relative arrangement of SSEs in space independent of their sequence. The probability of the sequence given this SSE arrangement, 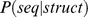, evaluates placement of specific amino acids into these SSEs. For the protein folding problem the probability of the sequence 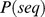 is a constant. The terms 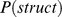 and 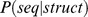 will each be expressed as a product of multiple contributing terms 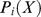.

### The Inverse Boltzmann Relation Converts Probabilities into an Approximation of Energy

The collected probabilities 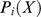 are converted into a free energy approximation using:
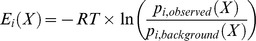



Where 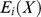 is the energy function for 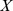 – being the feature observed, 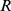 – the gas constant, 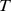 – temperature, 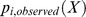 – the probability with which that feature was observed and 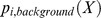 – the probability to observe that feature by chance. The normalization with 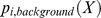 ensures that favorable states receive a negative energy, unfavorable states a positive energy. The energy unit 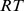 is arbitrarily defined as 1 BCL energy unit (BCLEU).

The most direct approach computes the total energy as sum of all individual contributions. One disadvantage of this strategy is that double-counting of contributions through several energy terms is difficult to entirely prevent. Other features of protein folds i.e. side chain hydrogen bonding or backbone interactions of loop residues will be ignored as they are not or only incompletely captured by the geometric features observed. To account for part of these inaccuracies, each energy term is scaled by an individual weight. This weight will be optimized to distinguish native-like from non-native models for a database of proteins.
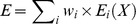



Another disadvantage of knowledge based potentials is the difficulty to assign an energy penalty to states not observed in protein structures. Typically small pseudo-counts are added which result in a positive energy. However, if a state is not observed at all, the energy assigned through a pseudo-count is arbitrary. To address this shortcoming, penalties for forbidden geometries are split into separate energy terms. Thereby the weight optimization procedure can assign a weight for these penalties independent from other contributions to the energy function.

While this approach is inherently imperfect it proved effective in the past. The resolution of protein models evaluated with the present energy function is too low to unambiguously distinguish native-like from non-native models based on energy alone. The objective of the energy function is to enrich for native-like topologies which can be done effectively in the presence of its inherent inaccuracies.

### Ensure Continuous Differentiability of All Geometric Parameters and Energy Potentials

Traditionally some geometric parameters observed contain step functions. An example is the number of neighbors within a given distance cutoff which is often used as a measure of solvent exposure [25], [31]. To avoid discontinuities at the cutoff, a continuously differentiable transition function is often introduced into the definition of a feature:
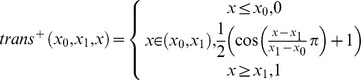


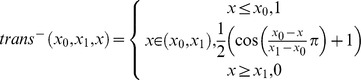



**Figure 1:**
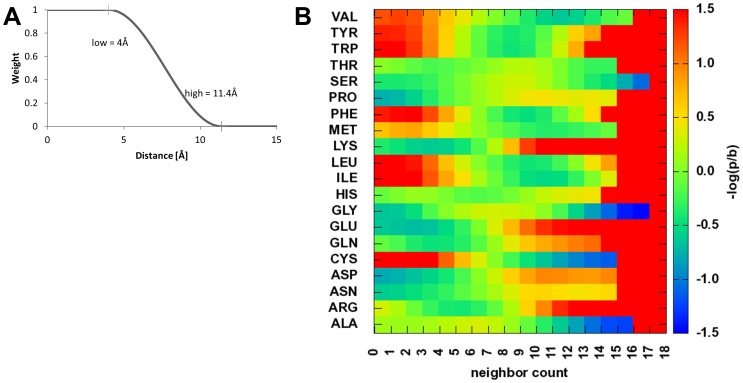
Amino acid neighbor count environment potential. A shows the transition function that is used between the lower and upper threshold in which the weight for the neighbor being considered drops from 1 (4 Å) to 0 (11.4 Å) using half of a cosine function. B shows the neighbor count energy potential for all 20 amino acids with their three letter code.

In Figure 1A an example of 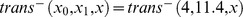 is shown, which is used to smooth the neighbor count (described below). The difference between 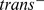 and 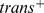 is that the first is a step-up, the latter is a step-down as a function of 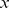. We demonstrated in the past that such a transition function allows for a neighbor count measure that is not only continuously differentiable but also more accurately approximates solvent accessible surface area [31].

### Amino Acid Environment Potential

This energy potential captures the preference of an amino acid to be buried and engage in a hydrophobic interaction in the protein core or to be exposed and interact with the solvent.
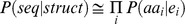



In order to measure burial, a function that counts the neighbors of an amino acid was used (Figure 1A):
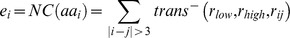



Weighing the actual neighbor count between 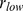 and 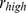 smoothens the potential and enables gradient based minimizations. The thresholds have been optimized for a high correlation of the neighbor count value with the MSMS solvent accessible surface area (SASA) approximation implemented in the molecular visualization package VMD [32]. The lower threshold is set to 4.0 Å, the upper threshold to 11.4 Å [31]. A minimal sequence separation of three residues reduces the bias introduced by sequence proximity. This step is particularly necessary to accurately determine exposure at the end of SSEs. In SSE-only protein models amino acids at the end of SSEs would otherwise have an artificially low neighbor count. The background probability distribution is the normalized sum of all normalized amino acid exposure distributions. Neighbor count bins that were empty or had one raw count were assigned a constant repulsive energy value of 18 BCLEU (Figure 1B).

### Amino Acid Pair Distance Potential


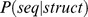
 is proportional to the amino acid pairs observed for a given distance.
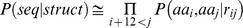



**Figure 2:**
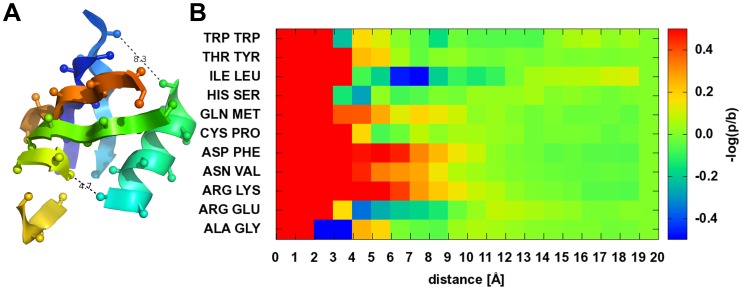
Amino acid pair distance potentials. In A the idealized structure of 1ubi with C_β_ and H_α_2 atoms is shown with the distances between ILE 32 and LEU 56 (4.7 Å) and between LYS 11 and GLU 34 (8.3 Å). B shows selected amino acid pair distance potentials for Trp-Trp as an example for π-stacking interaction, ILE-LEU as an example for vdW apolar interaction, ARG-GLU as an example for Coulomb attraction, and Arg-Lys as an example for Coulomb repulsion.

In order to define the interactions, statistics for the C_β_-atom distance between pairs of amino acids 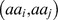 have been collected. For Glycine, the H_α2_ hydrogen position was used (Figure 2A). Distances have been collected between 0 and 20 Å in bins of size 1 Å. Amino acid pairs have been considered if they had a sequence separation of at least 12 residues 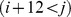 in order to reduce the bias introduced by sequence proximity. For each bin the energy was approximated using the inverse Boltzmann relation. The expected background probability is estimated through the frequency of seeing 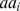 or 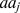 with any other amino acid at distance 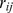. Distance bins that had fewer than five raw counts were assigned a constant repulsive energy value of 18 BCLEU (Figure 2). Note that a separate penalty will forbid very close distances not observed in protein structures. These would result in clashes of side chain atoms if implicitly present.

### Loop Length Potential

**Figure 3:**
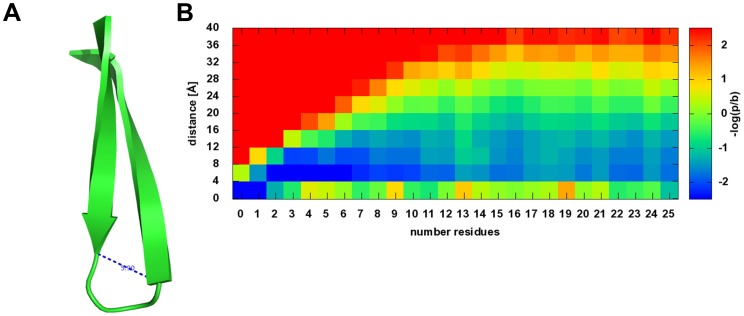
Loop closure potential. A describes two β-strands connected by a loop characterized by the Euclidean distance between the two ends and the number of residues in the loop connecting those two ends. B describes the derived energy potential, where the energy is a function of the number of residues in the loop and the Euclidean distance between the ends of the main axes.



SSEs are connected by loop or coil regions whose coordinates are not explicitly considered in the present approach to score protein folds. However, there are preferences for loops of a certain length 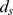 to bridge a certain Euclidean distance 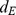 (Figure 3A). This is a sequence-independent score contributing to 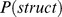. Note that the requirement that two SSEs can be physically linked with a fully extended loop is controlled by a separate loop closure penalty (read below).

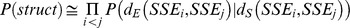




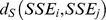
 Sequence distance between last residue of 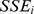 and first residue of 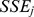



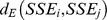
 Euclidean distance between end of main axis of last fragment of 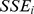 and beginning of main axis first fragment of 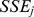
_._

The background probability is set to 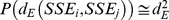 (Figure 3B). For short sequence distances it is favorable that the Euclidean distance is short. Long Euclidean distances are forbidden by a constantly increasing positive energy which is a result of the pseudo count divided by the square of the Euclidean distance. Euclidean distances below 4 Å are generally possible but are only preferred for loops of length 0 and 1 which occur in the database for bent and kinked SSEs. There is a nearly linear dependency between the sequence separation and the Euclidean distance for up to 7 residues in the loop. The maximally possible Euclidean distance increases linearly to a distance of approximately 32 Å at 10 residues. Euclidean distances longer than 32 Å are rarely observed in this database of globular proteins. As loops get longer, the range of Euclidean distance they bridge becomes wider.

### β-Strand Pairing Potential

This potential evaluates the pairing of two β-strand SSEs to form a β-sheet contact.
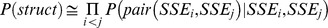



**Figure 4:**
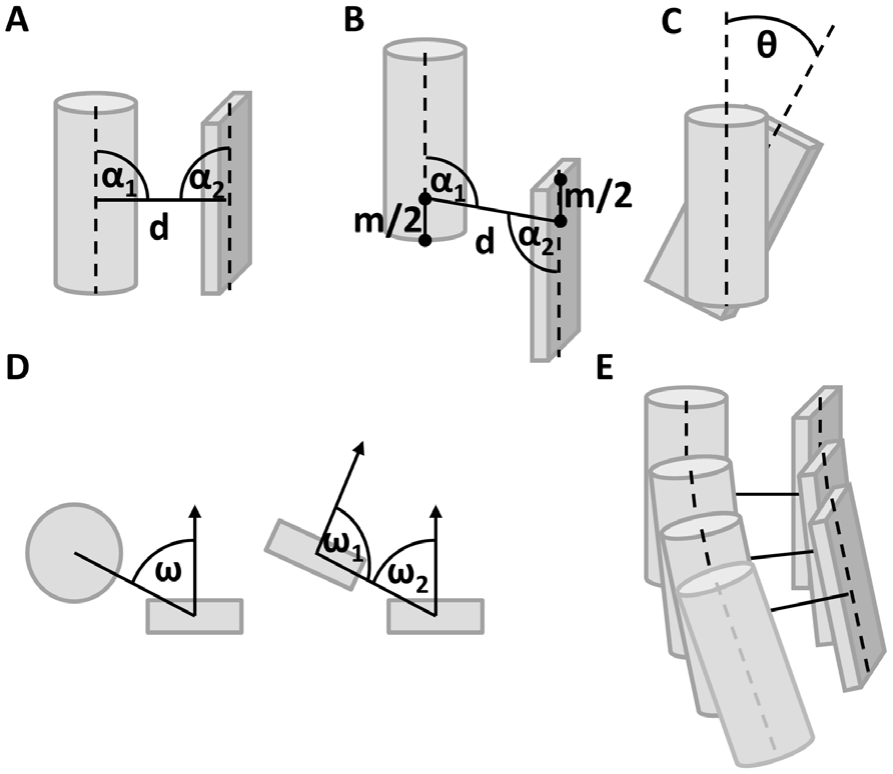
SSE Fragment packing. SSE fragments are shown with their geometric packing descriptors. A α_1_ and α_2_ are orthogonal, if the shortest connection between the main axes is orthogonal. B connection is not orthogonal, since the minimal interface length m cannot be achieved. C θ is the twist angle around the shortest connection – which is equivalent to the dihedral angle between main axis 1 – shortest connection – main axis 2. D ω is the offset from the optimal expected position for a helix-strand interaction, if it is 0°, the helix is on top of the strand, if it is 90°, the helix would interact with the backbone of the strand. ω_1_ and ω_2_ are the offsets for a strand-strand packing – for omegas close to 90°, it is a strand backbone pairing interaction dominated by hydrogen bond interaction within a sheet, if they are close to 0°, it is dominated by side chain interactions like seen in sheet-sandwiches. E every SSE is represented as multiple fragments and the SSE interaction is described by the list of all fragment interactions, leaving out additional fragments of the longer SSE with suboptimal packing (bottom grey helix fragment).



To compute 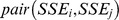 both strands are decomposed into overlapping fragments of three amino acids (Figure 4E). A β-sheet contact then is defined as a series of 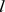 pairs of aligned fragments. The distance 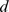 and torsion angle 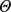 between each pair of fragments is evaluated (Figure 4A). Further, a weight 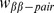 is used to distinguish a planar arrangement of two β-strands (β-strand pairing) from an opposing arrangement (β-sheet packing, Figure 4D, for details see Methods). 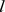 is limited to the number of fragments in the shorter SSE.:

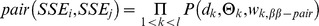




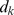
 shortest, orthogonal distance in fragment pair 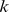



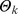
 torsion angle at shortest, orthogonal distance in fragment pair 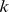



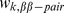
 weight that decreases as the arrangement deviates from planar β-strand pairing

**Figure 5:**
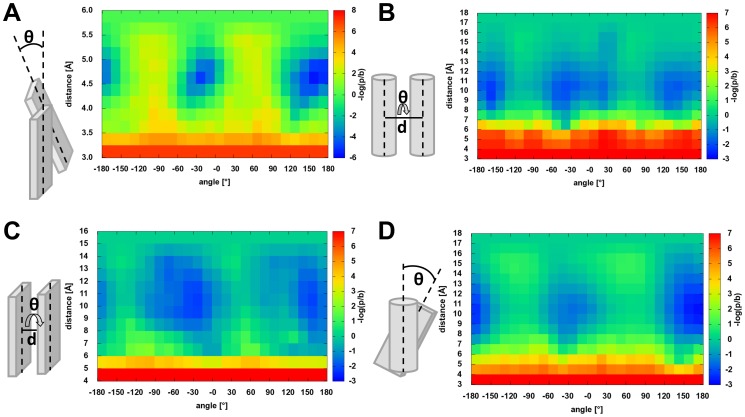
Strand pairing and SSE packing potential. Shown are all secondary structure element packing potentials with their schematic shortest connections, twist angle and their derived potentials. A shows the β-Strand-β-Strand pairing potential with prominent distance of 4.75 Å and angles of −15° and 165°. B shows the α-Helix-α-Helix packing with preferred packing distance of 10 Å and the preferred parallel angle of −45° and the anti-parallel packing of 135°. C shows the β-Sheet-β-Sheet packing potential with a preferred distance 10 Å and angles of −30° and 150 °. D shows the α-Helix-β-Sheet packing with its packing distance around 10 Å and an anti-parallel angle of 150°–180°.

The potential represents the likelihood of observing a given distance between the center of two β-strand fragments and a given twist of two β-strand fragments (Figure 5A) with respect to each other. Note that the potential omits explicit evaluation of backbone hydrogen bonds to keep the energy landscape smooth. The background probability is assumed to be proportional to 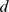 since the chance to find a second β-strand by chance in a parallel arrangements grows approximately linearly with the distance of the object, similar to the girth of a circle.

### Secondary Structure Element Packing Potential

While β-strand pairing is defined by backbone hydrogen bonds, SSE packing is driven through side chain interaction. As a result, distance and torsion angles are less tightly controlled which is why we treat both potentials separately. Aside from this separation, SSE packing potentials have been derived in a fashion similar to the β-strand pairing potential.
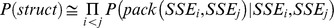



To compute 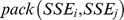 both SSEs are decomposed into overlapping fragments of three amino acids (β-strands) and five amino acids (α-helices, Figure 4E). A contact then is defined as a series of 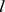 pairs of aligned fragments. The distance 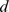 and torsion angle 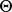 between each pair of fragments is evaluated (Figure 4, Figure 5B–D).
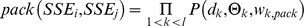




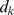
 shortest, orthogonal distance in fragment pair 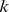



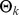
 torsion angle at shortest, orthogonal distance in fragment pair 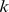



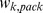
 weight that decreases if β-sheets in the packing interact via their edge

The term 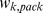 is dependent on the types of SSEs in the packing. For the helix-helix interaction, 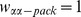. For helix-strand interactions, 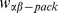 decreases from 1 if the face of the β-strand points away from the α-helix. For β-sheet packing, 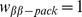 decreases from 1 if the β-strands don’t face each other (Figure 4D, details in Methods). The background probability is assumed to be proportional to 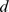. The resulting potentials plot energy with respect to distance and twist angle.

### Contact Order Score

**Figure 6:**
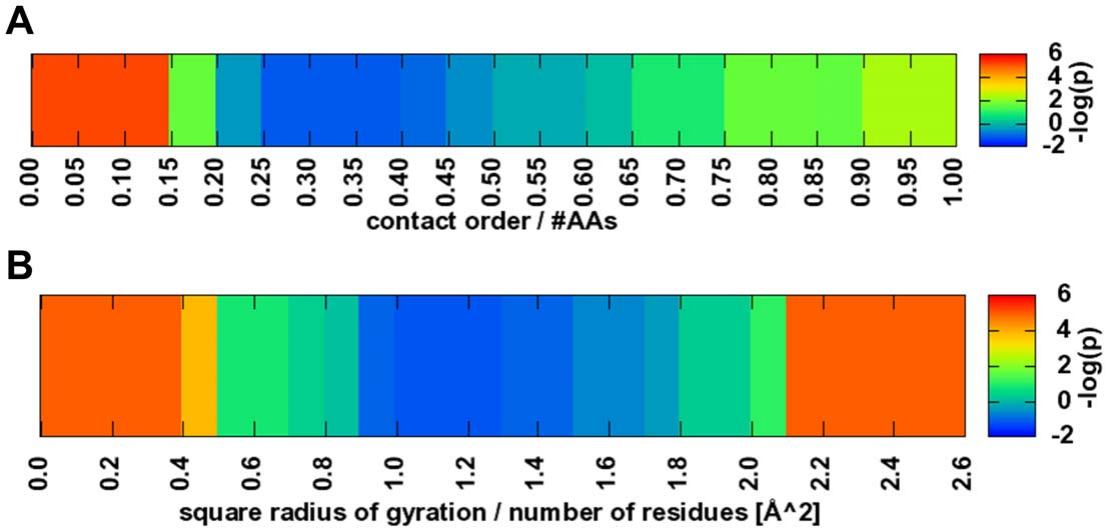
Contact order and square radius of gyration potential. A Fold complexity is represented by the contact order potential. The potential is given as the likelihood to observe a contact order to number of residues ratio in the model. B Statistics for the square radius of gyration over the number of residues were directly collected in a histogram and converted into a potential.



Using the assembly of SSEs to describe the topology of a protein enables the optimization protocol to sample topologies with many non-local contacts. One measure for the complexity of the topology is the contact order. Contact order 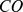 is defined as the average sequence separation of all amino acids in contact, conventionally identified by the closest heavy atom distance between two amino acids< = 8 Å [33]. In this score, the C_β_-C_β_ distance is used. A larger contact order constitutes a more complex topology. The contact order score is added to restrain the models constructed to a likely contact order range. To ensure comparability we normalize the square of the contact order with the sequence length to compute 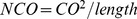. For native proteins, 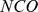 is largely independent of sequence length being in the range of 0.25 to 0.60 (Figure S1). An energy term (Figure 6A) was added based on the hypothesis:

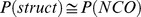



### Radius of Gyration Potential

The square of the radius of gyration is proportional to en energy term that describes the compactness of the fold [21]. It is computed as the mean square distance of all C_β_ atom coordinates (H_α2_ for Glycine) to their mean position:
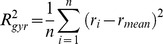



The term 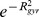 can directly be used to estimate 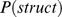 if sequence length is constant [34]. To enable the energy function to compare proteins of variable length e.g. during the assembly from SSEs, we introduce a normalized radius of gyration 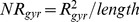. For native proteins, 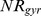 is largely independent of sequence length being in the range of 0.8 to 2.0 (Figure S2). An energy term (Figure 6B) was added based on the hypothesis:
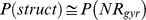



Extended α-helical coil-coiled structures as well as protomers that form obligate oligomers were removed prior to obtaining this statistic.

### Secondary Structure Prediction Agreement

Given an amino acid sequence, JUFO [35] and PSIPRED [36] calculate probabilities for each amino acid to be part of an α-helical, β-strand or a coil SSE. Those prediction methods average a per-residue accuracy of up to 80%. This fact can be used to evaluate the per-residue assigned secondary structure for a given protein model.
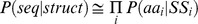




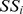
 secondary structure of amino acid *i* in the structure

Due to the inaccuracies in the secondary structure predictions, a mean probability and standard deviation for the probability for actual secondary structures are derived, and the error function of the standard score is defined as the potential used:
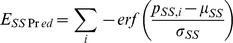




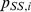
 probability of the assigned secondary structure in the model


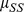
 mean probability for accurately predicted secondary structure


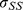
 standard deviation for accurately predicted secondary structure

The use of the standard score makes it possible to use different secondary structure prediction methods, of different sensitivity and dynamic range of probabilities. The error function projects the standard score in a less sensitive range if probabilities strongly disagree with the average. Parameters have been derived for JUFO and PSIPRED (Table 1).

**Table 1:** Mean and standard deviation of predicted probabilities.

	*µ_ss_* helix	*σ_ss_* helix	*µ_ss_* strand	*σ_ss_* strand	*µ_ss_* coil	*σ_ss_* coil
JUFO	0.67	0.21	0.58	0.24	0.59	0.18
PSIPRED	0.76	0.20	0.71	0.27	0.73	0.21

For secondary structure prediction (JUFO and PSIPRED) and secondary structure type, the predicted probabilities are averaged and a standard deviation is derived.

### Amino Acid Clash, SSE Clash and Loop Closure Constraint

A difficulty with knowledge based potentials is that a Boltzmann-like distribution is assumed for the dataset used from which the potentials are derived. Although all potentials described above are based on probabilistic theory, they are ambiguous to geometries absent in native structures. Since no counts are observed for these geometries the associated energies would be infinitely high. However, while the energy will be elevated it will not be infinite. The precise penalty for such non-native features remains difficult to evaluate. Often one pseudo count for every observation is added (according to the rule of succession, “Laplace rule”) giving all non-observed events an equally high penalty. The precise penalty for such non-native features is difficult to determine. To enable fine-tuning of the energy penalties in regions of non-observed events separate energy components are introduced. This procedure allows an independent choice of a weight changing the penalty amplitude in “structurally forbidden” regions. The procedure has a second advantage: vdW (van der Waals) repulsion is affiliated with steeply rising energies over a small change in distance. A separate potential allows for a finer binning of these penalty potentials when compared to the attractive counter-parts.

#### Amino acid pair clash

For the amino acid pair distance potentials, all occurring amino acid pair distances within protein structures have been calculated. They were binned with a resolution of 0.05 Å for each amino acid type pair. The first bin with counts>1, when iterating from shorter distances to larger distance, was determined to be the minimum permitted distance. Using this threshold, a “penalty” function is defined:
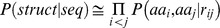


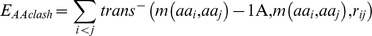




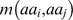
 Shortest allowed distance for amino acid type pair


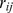
 Distance between amino acid pair

This term is complementary to the amino acid pair distance potential. If the distance between two amino acids is below the allowed distance for this pair of amino acid types, a positive energy penalty is applied, with a maximum at 1 Å below the allowed distance. A matrix of minimal distances for all amino acids types is depicted in the Figure S3.

#### SSE clash

Although the amino acid clash potential suffices in “detecting” clashes of side chains in the packing of SSE, it does not penalize special cases of overlapping SSEs. An example for these kinds of topologies is when one β-strand is positioned on top of another β-strand but offset by one amino acid. C_β_ atoms point in opposite directions avoiding any clash while backbone atoms are not explicitly modeled. To prevent such situations a clash term that is based on the packing SSE fragments was derived. From unoccupied bins in the SSE packing and pairing potentials (Figure 5) minimal distances between two SSE fragments have been defined as α-helix/α-helix 4 Å, α-helix/β-strand 4 Å, β-strand/β-strand 3 Å:
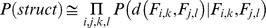


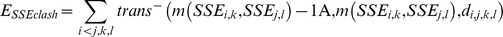




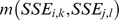
 Minimal allowed distance for aligned fragment pair 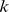 and 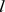 of SSEs 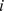 and 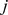



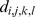
 Length of shortest connection between the two SSE fragments

This term is complementary to the SSE packing and β-strand pairing potential. If the distance between two SSE fragments is smaller than 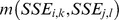, a positive energy is the result. The full positive energy is reached if the distance is 1 Å below the allowed distance for that pair of SSE types.

#### Loop closure constraint

In order to guarantee the possibility to close loops it proved necessary to add a steep penalty if the Euclidean distance between ends of SSEs becomes too long. In contrast to the loop length potential, the loop closure constraint only considers SSEs adjacent in sequence. The Euclidean distance between the terminal C atom and the starting N atom of the following SSEs 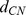 is evaluated.

s *d_CN_* is generally shorter than 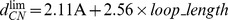. This relation was obtained by selecting the Euclidean distance for a loop length, which is the 5^th^ percentile of the longest distances. For lengths between one and twenty amino acids in the databank, a linear regression was fitted (Figure S4). We evaluate therefore 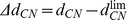:
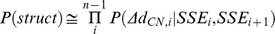


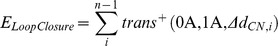



This potential is complementary to the loop length potential. It forbids loops that cannot be closed because of too large Euclidean distances. Additionally, it measures the distance between the two atoms that are the anchors for the loop, while the loop length potential is using a more crude estimation for the ends of the SSEs using only the tips of the fragment main axes.

### 53 Protein Model Sets have been Generated Using Rosetta, a BCL Perturbation Protocol and a BCL Folding Protocol

In order to benchmark the performance of the knowledge-based energy potentials, 53 diverse proteins have been selected and structural models were generated computationally using three methods: (1) Using Rosetta *de novo* protein structure prediction. (2) Removing loops from native structures and applying systematic perturbations to the structures. The sets of perturbations were chosen to generate models with preserved native-like topologies. (3) Re-assembling the SSEs present in the native structures leading to protein models of various arrangements and topologies. Details on the protocols are described in the Methods section.

The rationale for usage of three separate sets of protein models was to maximize diversity in the models thereby maximizing generalizability of the scoring function. The identification of native-like structures was based on three measures: (1) RMSD100<8 Å (C_α_ root mean square deviation normalized to a protein length of 100 residues, see [37]), (2) CR12>20% (contact recovery over 12 residues, see accompanying manuscript) and (3) GDT_TS>25% [38]. The percentage of native-like models varies between 0 and 99.5% for the protein model sets. Only model sets with percentage of native-like models between 1% and 99% have been used for the analysis in a ten-fold cross validation calculation of enrichments. The cross validation subsets have been generated by randomly removing models so that each subset contained 10% correctly folded models and 90% incorrect models.

### Enrichment is a Good Measure to Evaluate the Performance of an Energy Potential

Figure S5 shows a representative RMSD100-energy plot of a set of protein models that was prepared to contain 10% of native-like models below an 8 Å RMSD100 cutoff. The 8 Å cutoff is based on the observation that two protein models typically share the same topology below that measure. The horizontal line denotes the best 10% of the models with respect to the scoring function used. Models that are below the RMSD100 cutoff are positives 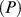, and if they are below the energy of the best 10% by energy, they are considered as true positives 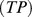. If the model has a high energy despite being correct by the RMSD100, it is considered a false positive 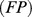. 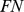 – false negative and 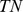 – true negative are defined similarly. The optimal result would be to have empty 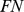 and 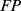 quadrants, because this would indicate that the energy function would be completely accurate in identifying native-like models by RMSD100. The enrichment is now defined by the ratio of true positives within the 10% native-like models 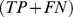 divided by the initial ratio of native-like models (defined by the RMSD100 cutoff) to the total number of models 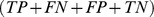.
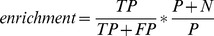



For the following benchmark 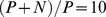 is set, limiting the maximal enrichment to 10. An enrichment of 1 corresponds to no improvement. Enrichment values smaller than 1 suggest that the score deselects native-like SSE arrangements.

### Benchmark Enrichment of Native Like Structures Through Potentials

Table 2 contains enrichments for the 53 protein sets from three different methods each, and the various scores. Note that the number of proteins considered can be smaller than 53 if the number of native-like models was insufficient to confidently determine enrichment (read above). Statistical significance was established by computing the average enrichment over 10 cross-validations, subtracting the expected mean of 1.0 (for a non-discriminating potential), and dividing the result by the standard deviation of the enrichment over of the 10 cross validation sets (Z-score). The percent of model sets that are enriched by a statistically significant factor are reported (Z-score>1.0, Table 2). In comparison to the balanced performance of the consensus scoring with an optimized weight set (Table 3), individual components of the scoring function generally discriminate well against random models for the BCL folded and perturbed structures but do perform worse for Rosetta folded models. This observation is attributed to the fact that Rosetta folded models will generally score well in the BCL::Score energy function due to the similarity of the two scoring schemes. The amino acid pair distance, amino acid neighbor count and the SSE packing potentials achieve enrichments greater than 1.0 for nearly all the protein sets. The secondary structure prediction scores using PSIPRED secondary structure probabilities enrich Rosetta and perturbation model sets, which have varying SSE content. BCL folded models cannot be discriminated, since the secondary structure is constant. The consensus scoring function enriches significantly (67% of Rosetta, 77% of perturbation model sets for RMSD<8 Å). No statistically significant improvement for BCL folded models is observed. We attribute this to the fact that these models were subject to BCL::Score energy evaluation during folding creating a circular dependence. Considering the performance with respect to GDT_TS>25%, for the three different models sets, 80%, 94% and 83% have a significant enriched model set for Rosetta as well as BCL perturbed and folded model sets.

**Table 2:** Enrichment of sets of protein models.

RMSD100<8 Å			total		amino acid clash	amino acid distance	amino acid neighbor count	contact order	loop length	loop closure	radius of gyration	SSE clash	SSE packing	strand pairing	SSPred JUFO	SSPred PSIPRED	sum
all		rosetta	18		*44*	72	56	44	*22*	*44*	61	*50*	100	33	56	78	67
		perturbation	53		100	98	96	21	94	98	49	96	89	57	47	60	77
		fold	14		64	57	29	29	64	79	29	36	29	0	29	29	43
α-helical		rosetta	12		*58*	83	58	42	*25*	*50*	58	*67*	100	*17*	50	67	58
		perturbation	24		100	96	92	17	92	100	58	92	75	*4*	42	46	63
		fold	10		60	70	30	30	50	80	20	30	40	*0*	40	40	50
β-sheet		rosetta	3		*0*	67	33	100	*0*	*33*	33	*33*	100	67	33	100	67
		perturbation	8		100	100	100	38	100	100	50	100	100	100	25	25	75
		fold	3		67	33	33	33	100	67	67	67	0	0	0	0	33
α/β		rosetta	3		*33*	33	67	0	*33*	*33*	100	*0*	100	67	100	100	100
		perturbation	21		100	100	100	19	95	95	38	100	100	100	62	90	95
		fold	1		100	0	0	0	100	100	0	0	0	0	0	0	0
≤150 AA		rosetta	12		*58*	92	58	50	*17*	*33*	58	*50*	100	25	42	75	67
		perturbation	17		100	94	100	29	100	100	76	94	82	47	41	41	88
		fold	9		67	44	22	22	78	89	22	22	11	0	22	22	22
>150 AA		rosetta	6		*17*	33	50	33	*33*	*67*	67	*50*	100	50	83	83	67
		perturbation	36		100	100	94	17	92	97	36	97	92	61	50	69	72
		fold	5		60	80	40	40	40	60	40	60	60	0	40	40	80
**GDT_TS>25%**			**total**		**amino acid clash**	**amino acid distance**	**amino acid neighbor count**	**contact order**	**loop length**	**loop closure**	**radius of gyration**	**SSE clash**	**SSE packing**	**strand pairing**	**SSPred JUFO**	**SSPred PSIPRED**	**sum**
all	rosetta		30		*23*	53	70	7	*33*	*13*	67	*47*	83	47	63	80	80
	perturbation		52		71	75	94	35	87	79	40	62	98	60	71	87	94
	fold		18		39	61	44	33	61	50	33	22	56	11	39	56	83
α-helical	rosetta		17		*18*	59	59	12	*12*	*12*	47	*53*	82	*18*	41	76	65
	perturbation		24		54	58	92	46	71	63	42	38	100	*13*	79	92	96
	fold		13		38	69	46	31	54	54	23	8	54	*0*	38	54	77
β-sheet	rosetta		5		*20*	40	80	0	*60*	*20*	80	*40*	80	80	80	80	100
	perturbation		8		63	75	88	38	100	88	50	75	100	100	75	75	88
	fold		2		0	50	50	100	100	50	50	50	0	50	50	50	100
α/β	rosetta		8		*38*	50	88	0	*63*	*13*	100	*38*	88	88	100	88	100
	perturbation		20		95	95	100	20	100	95	35	85	95	100	60	85	95
	fold		3		67	33	33	0	67	33	67	67	100	33	33	67	100
≤150 AA	rosetta		12		*8*	42	42	8	*17*	*17*	50	*17*	58	33	67	75	75
	perturbation		17		41	53	88	53	76	47	53	35	94	59	65	82	100
	fold		11		27	64	55	36	73	45	36	27	36	9	45	64	82
>150 AA	rosetta		18		*33*	61	89	6	*44*	*11*	78	*67*	100	56	61	83	83
	perturbation		35		86	86	97	26	91	94	34	74	100	60	74	89	91
	fold		7		57	57	29	29	43	57	29	14	86	14	29	43	86
**cr12>10%**			**total**	**amino acid clash**		**amino acid distance**	**amino acid neighbor count**	**contact order**	**loop length**	**loop closure**	**radius of gyration**	**SSE clash**	**SSE packing**	**strand pairing**	**SSPred JUFO**	**SSPred PSIPRED**	**sum**
all	rosetta		20	*20*		50	80	30	*40*	*10*	65	*55*	85	50	65	75	75
	perturbation		25	64		68	100	32	100	92	52	52	100	52	68	88	100
	fold		35	51		83	69	20	71	60	23	43	69	40	26	34	46
α-helical	rosetta		11	*27*		64	73	36	*27*	*0*	55	*64*	82	*9*	45	55	64
	perturbation		12	33		42	100	33	100	100	67	25	100	*0*	75	92	100
	fold		12	42		75	58	25	50	58	17	33	58	*8*	42	50	42
β-sheet	rosetta		4	*0*		50	100	50	*50*	*0*	50	*50*	75	100	75	100	75
	perturbation		3	67		67	100	33	100	67	67	67	100	100	67	67	100
	fold		8	38		88	75	25	88	63	13	63	63	63	13	13	38
α/β	rosetta		5	*20*		20	80	0	*60*	*40*	100	*40*	100	100	100	100	100
	perturbation		10	100		100	100	30	100	90	30	80	100	100	60	90	100
	fold		15	67		87	73	13	80	60	33	40	80	53	20	33	53
≤150 AA	rosetta		13	*23*		62	85	38	*38*	*8*	62	*62*	85	38	46	69	69
	perturbation		11	55		64	100	36	100	91	73	45	100	36	55	82	100
	fold		14	36		79	71	21	71	71	21	36	50	21	29	43	43
>150 AA	rosetta		7	*14*		29	71	14	*43*	*14*	71	*43*	86	71	100	86	86
	perturbation		14	71		71	100	29	100	93	36	57	100	64	79	93	100
	fold		21	62		86	67	19	71	52	24	48	81	52	24	29	48

For each score and benchmark set, the percentage of protein model sets that had significant improvement in enrichment (Z-score>1.0) for each of the knowledge based potentials are displayed. Three classifications for native-like models were used (RMSD, GDT_TS and CR12), and protein model sets have been classified as α with #helices ≥ 2, as β with #strands ≥ 2, and αβ if both conditions are fulfilled. Proteins were also classified as small when having ≤ 150 amino acids. Cells with bold percentages highlight the cases where for more protein model sets a significant improvement in enrichment was achieved versus worsening. Cells in italic are discussed and expected to not enrich the respective model set (for discussion, please see text).

**Table 3:** Weight set for consensus scoring function.

AA distance	AA neighbor	loop length	Radius of gyration	Loop closure	AA pair clash	SSE clash	SSE packing	Strand pairing	Contact Score	SSPred JUFO	SSPred PSIPRED
0.35	50	10	5	500	500	500	8	20	0.5	5	20

Monte Carlo optimization maximized the enrichment over the Rosetta model set. Loop closure, AA pair and SSE clash weights were set to 500. This weight set was used to calculate the score sum, as used to calculate enrichments for the benchmark set.

### BCL::Score Potentials Recapitulate Expected Amino Acid Interaction Preferences

The scoring function was developed for protein models consisting of disconnected, idealized SSEs. The absence of atomic-detail in the SSE-only protein models inherently prevents unambiguous identification of the native conformation in a set of models. Nevertheless, the amino acid pair potential and the amino acid environment potential both select for native-like arrangements of amino acids. The environment potential follows the expected trend preferring around three neighbors for the negatively charged Glutamate residue but around eleven neighbors for the apolar Valine. For Glycine two minima are observed – very few and very many neighbors. This is somewhat counter-intuitive as Glycine prefers exposed positions in loop regions. However, the potential 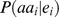 reflects the probability of encountering a specific amino acid type given a certain exposure value, rather than the most probable exposure for a given amino acid type. In densely packed positions with an extremely high number of neighbors only Glycine will fit giving it the high probability for such positions. Positions with neighbor counts above twelve are rare in folded proteins and should therefore be disfavored when predicting protein structures. However, this fact will be represented by 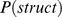 and is correctly omitted in 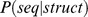. Leucine and Isoleucine are expected to interact favorably in the pair potential due to vdW attraction, which is reflected by the negative energies for short distances (Figure 2B). Arginine and Lysine with positively charged side chains are expected to experience Coulomb repulsion when approaching each other which is reflected by the positive energy for short C_β_-atom distances. Tryptophan pairs may engage in π-stacking interactions, which are evidenced by a preferred C_β_-atom distance around 4 Å (β-strand pairing) and 8 Å (SSE packing). Arginine and Lysine are both positively charged and repel each other at close proximity as reflected by the positive energies for C_β_-atom distances smaller 10 Å. These findings imply that for reduced SSE-only protein models a C_β_-atom side chain representation (H_α2_ for Glycine) is sufficient to estimate 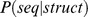.

### Secondary Structure Element Arrangement Determines the Domain Topology

The preferential arrangement of SSEs in a protein domain results from the sum of many atom-atom interactions. In the absence of atomic-detail in SSE-only protein models, BCL::Score knowledge-based potentials derived from 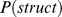 discriminate native-like SSE arrangements. An optimal β-strand distance between 4.25 and 5.00 Å is observed. The optimal twist angle is around −15° (parallel β-strand contact) and 165° (anti-parallel β-strand contact). A twist angle of 165° is more pronounced as anti-parallel β-strand contacts are slightly overrepresented in the database. Two α-helices pack in a preferred angle of −45°. The anti-parallel packing is slightly less common at around 135°. Further, weak minima around 15° and −165° are observed. Both cases of packing have a preferred distance of 9–12 Å (Figure 5 Figure 5B). For α-helix-β-sheet packing, the anti-parallel case with angles between 150° and 180° is most common as seen in the TIM-barrel fold or other “Rossman-Folds” [32] (Figure 5D). As for the α-helix-α-helix packing, the optimal distance is around 9–12 Å. β-sandwiches pack with a distance of 9–12 Å and twist angles of −30° or 150° (Figure 5C). Twist angles lead in general to an improved packing as the interacting side chains can reach into gaps left by the side chains of the opposite SSE [33]. Ridges and grooves are formed on the surface of helices. These ridges are formed by residues usually separated by four in sequence. This model explains the predominant packing angle of around 50°.

### Maximal Enrichment is Limited Due to the Incomplete, Reduced Representation of Protein Structure

The maximum enrichment for any of the scores for any set of models is never above 5 (Table S1). We attribute this finding to two limitations of BCL::Score: Firstly, the protein models used are incomplete. Contributions of loop and coil regions to the overall energy are neglected resulting in inherent inaccuracies. Secondly, amino acids are represented by their C_β_-atom only. This procedure introduces additional inaccuracies in the energetic evaluation. As discussed in the introduction, these inaccuracies are taken into account to enable a more rapid sampling of domain topology specifically in a limited experimental data setting. Subsequently protein models can be completed and refined using higher accuracy all-atom energy functions. Nevertheless, BCL::Score knowledge-based potentials enrich a diverse set of decoys with enrichments up to 7 for individual proteins with respect to the weighted consensus score (Table S1, last column). This is a respectable achievement in particular when keeping in mind that some of the protein models are created using an energy function that necessarily covers some or even most aspects of the BCL::Score knowledge-based potential (model sets 1 and 3). Additionally, the other models start from experimental protein structures (model set 2). Accordingly the model sets contain many native-like features that are expected to score well with BCL::Score.

### C_β_ Atom is Sufficient to Approximate Side Chain Position

The amino acid pair potential and the amino acid environment potential are both successful in discriminating for native-like protein structures. This implies that a C_β_ atom side chain representation (H_α2_ for Glycine) is sufficient not only for describing possible interactions with other amino acids as a pair potential but also as an environment potential.

### Enrichment was Achieved for a Diverse set of Protein Models Regardless of the Sampling Algorithm

We tested BCL::Score potentials in conjunction with Rosetta-generated models (model set 1) to assess the general applicability of the scoring approach. Rosetta models have a complete and defined backbone conformation. All BCL::Score potentials except for the loop length and contact order score can enrich Rosetta models for native like conformations. It is expected that the loop length potential will not enrich Rosetta models as they have a continuous amino acid chain. The loop length potential enriches BCL perturbed and folded structures with a discontinued amino acid chain. Due to the unrestrained sampling of the secondary structure elements, loops are violated and the potential is penalizing this arrangement. The contact order score prevents low and highly complex folds if several SSEs are swapped or not in close proximity. This is the case for BCL folded and perturbed structures, where the potential helps regardless of size and SSE composition, but unlikely in Rosetta models which are biased towards lower contact orders. As expected, the β-strand pairing score contributes only for β-strand containing proteins. The radius of gyration score performs well for proteins<150 residues, but seems to degrade for larger proteins. It can be observed that for GDT_TS and RMSD100 classification, the percentage drops under 50% for the BCL perturbed structures. This is expected as this model set was created to preserve protein size and relative positioning of SSEs that is native-like but create non-native topologies. We observe the best consensus function discrimination for native like models for this model set. The weighted sum of individual terms performs comparably over all benchmark sets and shows that a linear combination can overcome some weaknesses of the individual terms.

### BCL::Score Ranking and Enrichment Performance in Comparison to Other Energy Potentials

Table 4 shows the rank of the native structure for different small decoy sets (∼500 models) of the “decoys‘r’us” protein model sets [39]. The ranks for the energy function in the comparison are extracted from experiments for the DOPE potential [20]. Although BCL::Score energy potentials were not designed for full atom protein models represented in the protein model sets, it can rank the native first for 52% of the sets. Of the six tested energy potentials success rates vary (24%, 40%, 48%, 52%, 84%, 84%) placing BCL::Score somewhere in the middle. Keeping in mind, that the other scoring functions leverage additional detail, some even atomic detail, this is a respectable performance. BCL::Score filters reliably models of unlikely overall topology but has difficulty ranking models with native-like topologies. This notion is reinforced when ranking the native structure among the top ten models is counted as success. The BCL::Score success rate increases to 72% compared to 48%, 56%, 60%, 68%, 84% and 84% seen for the other energy functions.

**Table 4:** Ranking of native structure within different decosy‘r’us model sets.

set	Pdb-Chain	DFIRE	Rosetta	ModPipe-Pair	ModPipe-Surf	ModPipe-Comb	DOPE	BCL::Score
fisa	1fc2	254	158	491	1	453	375	480
fisa	1hdd-C	1	90	293	18	135	1	60
fisa	2cro	1	26	11	146	19	1	1
fisa	4icb	1	1	196	2	167	1	1
**correct**		**3**	**1**	**0**	**1 (2)**	**0**	**3**	**2**
fisa_casp3	1bg8-A	1	1068	1	1180	282	1	9
fisa_casp3	1bl0	1	960	4	912	86	1	246
fisa_casp3	1jwe	1	1177	1	1119	6	1	1
**correct**		**3**	**0**	**2 (3)**	**0**	**0 (1)**	**3**	**1 (2)**
lmds	1b0n-B	430	300	56	186	18	34	182
lmds	1bba	501	174	501	117	444	501	469
lmds	1fc2	501	291	325	54	222	476	501
lmds	1ctf	1	1	1	1	1	1	12
lmds	1dtk	1	9	4	1	1	1	4
lmds	1igd	1	1	1	3	1	1	1
lmds	1shf-A	1	5	24	18	7	1	2
lmds	2cro	1	2	4	28	12	1	1
lmds	2ovo	1	29	5	8	2	1	1
lmds	4pti	1	4	1	44	1	1	3
**correct**		**7**	**2 (6)**	**3 (6)**	**2 (4)**	**4 (6)**	**7**	**3 (6)**
lattice_ssfit	1beo	1	1	1	1	1	1	1
lattice_ssfit	1ctf	1	1	1	1	1	1	1
lattice_ssfit	1dtk-A	1	1	1	35	1	1	1
lattice_ssfit	1fca	1	1	1	4	1	1	1
lattice_ssfit	1nkl	1	1	1	1	1	1	1
lattice_ssfit	1pgb	1	1	1	3	1	1	1
lattice_ssfit	1trl-A	1	45	1	123	1	1	6
lattice_ssfit	4icb	1	1	1	3	1	1	1
**correct**		**8**	**7**	**8**	**3 (6)**	**8**	**8**	**7 (8)**
**sum rank 1**		**21**	**10**	**13**	**6**	**12**	**21**	**13**
**sum rank 10**		**21**	**14**	**17**	**12**	**15**	**21**	**18**

For different model sets from “decoys‘r’us” [39], the rank of the native structure, using different energy potentials, was determined. Ranks for DFIRE through DOPE were copied from the “DOPE” publication [20]. For each different model set, the number of sets for which the native was ranked 1^st^ was counted and reported. In brackets ranks among the top 10 were counted as correct.

Table 5 summarizes enrichments for the “moulder” decoy set [20] for different energy potentials. BCL::Score is able to enrich all of the model sets by a factor between 2.44 and 4.57. This performance is clearly reduced when compared to full atom potentials which achieve enrichments between 2.67 and 8. This decoy set was created by threading different sequences on similar homology models resulting in protein models with native-like overall topologies. As a result, the strength of the BCL::Score functions in discriminating non-native topologies is not assessed in this experiment.

**Table 5:** Enrichment of native like structures within the moulder decoy set.

pdbid	RMSD criteria [Å]	DFIRE	Rosetta	ModPipe-Pair	ModPipe-Surf	ModPipe-Comb	DOPE	BCL::Score
**1bbh**	3.53	7.00	7.33	5.00	8.33	7.33	8.67	4.22
**1c2r**	5.83	6.33	8.00	5.00	5.33	7.00	7.67	3.45
**1cau**	7.81	5.00	7.00	4.33	6.67	5.67	5.00	3.36
**1cew**	11.36	4.33	3.00	4.00	3.00	3.33	4.00	2.44
**1cid**	4.69	5.33	5.67	4.33	5.67	5.00	5.67	4.48
**1dxt**	3.52	5.33	4.33	5.00	4.67	5.33	6.67	2.93
**1eaf**	9.34	5.00	7.00	5.00	6.67	6.00	6.00	3.85
**1gky**	10.77	8.00	7.00	8.00	8.33	9.00	8.67	2.93
**1lga**	5.08	5.33	3.33	2.67	3.00	2.33	5.33	3.19
**1mdc**	3.26	7.67	6.00	4.33	6.33	6.00	7.67	4.40
**1mup**	4.83	8.00	6.33	7.33	7.67	7.67	8.67	4.57
**1onc**	3.60	7.33	6.67	6.67	7.67	7.00	7.67	4.05
**2afn**	5.47	4.67	6.00	5.00	5.67	6.67	4.00	3.45
**2cmd**	3.80	5.67	5.33	3.33	4.33	4.33	5.00	3.53
**2fbj**	5.06	7.33	7.00	7.00	6.67	7.33	6.33	3.71
**2mta**	3.52	4.00	5.00	2.67	4.33	3.67	4.33	4.20
**2pna**	3.89	6.33	5.33	6.33	7.33	6.67	7.33	4.05
**2sim**	6.13	6.67	4.67	4.00	4.00	4.00	6.00	3.36
**4sbv**	14.50	5.00	6.00	5.67	4.33	5.67	5.00	4.05
**8i1b**	4.17	5.67	5.33	4.00	4.67	5.67	5.33	3.65
**Average**		6.00	5.82	4.98	5.73	5.78	6.25	3.69
**Median**		5.67	6.00	5.00	5.67	5.84	6.00	3.68
**Min**		4.00	3.00	2.67	3.00	2.33	4.00	2.44
**Max**		8.00	8.00	8.00	8.33	9.00	8.67	4.57

For different model sets of the “moulder” decoy set [20], 10% enrichments were calculated. The 10% enrichment for different model sets also implies different RMSD cutoffs. The average, median, minimum, and maximum enrichment over the model sets is reported.

### Conclusions

A knowledge-based scoring function is presented optimized for SSE-only models. It enriches native-like topologies in diverse sets of protein models. We expect this scoring to be beneficial for certain settings in *de novo* protein structure determination: (1) When folding large proteins with complex topology, where simultaneous sampling of SSE arrangements and loop conformations would create a size limit for *de novo* protein structure determination. The BCL::Score potential for SSE-only models allows sampling of SSE arrangement independent of and prior to the sampling of loop conformations. This approach has the potential to increase the size limit in *de novo* protein structure determination. (2) Limited experimental datasets often restrain the position of SSEs, for example density maps obtained form cryo-Electron Microscopy [40] or EPR distance restraints [41]. We expect that the present potential can be applied to assemble the topology of large proteins from such datasets. In fact, an early version of BCL::Score has been successfully applied to medium resolution density maps form cryo-Electron Microscopy [9].

## Materials and Methods

### Divergent Databank of High Resolution Crystal Structures

Statistics have been derived from a divergent high resolution subset of the protein data bank (PDB) which was generated using the protein sequence culling server “PISCES” [42]. With a sequence identity limit of 25%, resolutions up to 2.0 Å, a maximum R-value of 0.3, sequence lengths of 40 residues minimum only X-ray structures have been culled from the PDB. This guarantees that similar sequences are not over represented, introducing a bias to proteins that are amenable to crystallography or are of higher interest in the scientific fields. All membrane proteins have been excluded. The resulting databank has 4,379 chains in 3,409 PDB entries. This approach to create the representative protein database might leave multiple members of the more popular fold groups thereby over-representing certain secondary structure packing motifs. An alternative approach would be a non-redundant fold databank created from SCOP [43] or CATH [44] classifications. Our rational for the first approach is that a non-redundant fold database would not cover the diversity of amino acid environments and interactions that are found within similar folds of diverse sequence worsening the statistics of the amino acid centric potentials. Further we argue that secondary structure packing motifs are conserved beyond the boundaries of individual folds. The statistics describing these packing interactions should therefore not be biased by occasional repetition of one fold group.

### Secondary Structure Element Packing

In order to develop statistics for the packing between two SSEs, SSE pairs were collected from protein models in the databank. α-helices with a length<7 residues and β-strands<5 residues have been ignored, and α-helices or β-strands have been described as overlapping sets of fragments of the length of 5 residues for α-helices and 3 residues for β-strands (Figure 4A). An ideal SSE fragment was superimposed with the backbone coordinates of the SSE fragment from the PDB to determine the orientation (translation and rotation in Euclidean space) of this fragment. The main axes have been considered to be line segments; a minimal interface length between the two SSE fragments of 4 Å was achieved by subtracting 2 Å from each end of each SSE’s main axis (Figure 4B). The packing between two fragments was described by the analytically shortest connection between those two line segments. If this connection was orthogonal, it was considered to be a full contact. If the connection was not orthogonal, a contact weight was defined as a function of the angle between the main axes and the shortest connection. This angle between 90° and 0° was then used to determine a weight between 0 and 1 using half of a cosine function and for both angles those weights are multiplied.
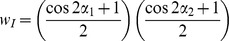



The twist between the SSE fragments is defined by the dihedral angle θ between the SSE main axes (Figure 4C). The relative offset, which is important when strand backbone hydrogen interactions could play a role, is defined by the offset angle ω between 0° and 90° (Figure 4D). For a strand-helix packing, only one offset angle can be defined, where an ω close to 90° is not favorable, a packing with an offset of 0° is desired, since it is dominated by amino acid side chain interactions. The weight is defined:
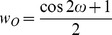



If two strands are involved in the interaction, it is necessary to distinguish a strand-strand backbone hydrogen bond mediated packing and a sheet-sheet (sandwich-like) amino acid side chain mediated interaction. For omega values near 90° it has a strand-strand interaction character; if the omega values are close to 0°, it is considered to be a sheet-sandwich interaction. Two weights can be defined:
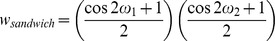


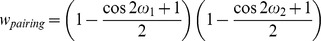



The packing between two SSEs is represented as a list of fragment interactions (Figure 4E), with distance and dihedral angle. For each fragment of the shorter SSE, the interaction weight to every fragment of the longer SSE (for identical sizes, the SSE that comes first in sequence is the “shorter” one) is calculated and the fragment pair with the highest interaction weight *w_I_* is added to the list of packing interactions. Since this is done for every fragment in the shorter SSE, the list will have as many entries as the number of fragments in the shorter SSE. Every packing interaction within this list is then considered for the statistics using the weight as the count. During scoring, all interactions in the list are scored, multiplied with the respective interaction weight and summed.

### Generation of Benchmark Sets

The benchmark sets of protein models were generated using three different methods. 53 sequences of length between ∼70 up to ∼300 residues have been selected to represent diversity regarding α-helical and β-strand content as well as sequence length : 1AAJA, 1BGCA, 1BJ7A, 1BZ4A, 1CHDA, 1DUSA, 1EYHA, 1G8AA, 1GAKA, 1GCUA, 1GS9A, 1HYPA, 1IAPA, 1ICXA, 1IFBA, 1J27A, 1JL1A, 1K6KA, 1LKFA, 1LKIA, 1LWBA, 1M5IA, 1NFNA, 1OA9A, 1OZ9A, 1PRZA, 1ROAA, 1TZVA, 1UBIA, 1UEKA, 1VGJA, 1VK4A, 1WBAA, 1WNHA, 1WR2A, 1WVHA, 1X91A, 1XGWA, 1XKRA, 1XQOA, 2CWYA, 2E3SA, 2EJXA, 2FM9A, 2ILRA, 2IU1A, 2OF3A, 2OPWA, 2OSAA, 2YV8A, 2YVTA, 2ZCOA, 3B5OA.

Three benchmark sets were created:

- Using Rosetta [45] 10,000 models have been folded *de novo* for each sequence. Since Rosetta does not assign secondary structure, DSSP [46] was used to add definitions to the models.
- 10,000 models each have been folded using the BCL::Fold program. For these simulations a scoring function with weights set to 1 was used. Further details on the folding simulations can be gleaned from a companion manuscript “De novo prediction of complex and large protein topologies by assembly of secondary structure elements” in the same issue of this journal.
- Additionally, 12,000 perturbed structures have been generated using the BCL::Fold program by starting with the native SSE arrangement and applying randomly the following perturbations to the starting structure: (1) SSE rotation and translation; (2) SSE flip; (3) swapping two SSEs; and (4) SSE removal.

Native-like models or positives were defined using three quality metrics: RMSD100 cutoff of 8 Å to the native, a GDT_TS cutoff of 25% and a contact recovery of 20% (see accompanying manuscript). The remaining models in each set were considered negatives or non-native-like. If there were less than 1% or more than 99% native-like models, that set was ignored for further analysis, since it indicates that the sampling algorithm is not suitable for that protein’s structure, either creating too many or too few native-like models. The ratio native-like/non-native-like is dependent on the performance of each protocol. The maximum enrichment a score can achieve is dependent upon this ratio. In order to facilitate comparison of the enrichment values, random sub-sets of models were created that contained 10% native-like models. For this, overall ratio in the complete sets of models had to be adjusted. The class of models over-represented with respect to the desired 1∶9 ratio (native or non-native like) was split into ten equally large subsets. From the under-represented class random models were added until the desired 1∶9 ration of native to non-native like models was achieved. This procedure uses all generated models without re-using models from the over-represented class. The enrichment values reported are the average over the ten experiments (Table S1).

### Weight Optimization

An optimized weight for the consensus scoring function was determined to calculate the sum of the scores (Table 3). The objective for optimizing the weight set was to maximize the sum of the square root of enrichments. This sum is calculated over a Rosetta model set of 53 proteins. Each protein model set is divided into 10 sets, of 10 % native-like models (RMSD100<8 Å) and 90% random structures (RMSD100 ≥ 8 Å), while the actual composition is randomly chosen from a set of 10,000 model structures.

The start weighting set is set to the inverse standard deviation of the score over the 53 * 10,000 models, so that the dynamic ranges of the scores are scaled to the same range. For every step two randomly chosen weights are modified randomly by adding or subtracting 10% of the starting weight, limiting the weights to a minimal value of 0. The minimization follows a Monte-Carlo/Metropolis simulated annealing protocol [47], [48] with 10,000 iterations maximum, terminating after 250 steps without improvement of the objective.

### BCL::Score Availability

All components of BCL::Score, including scoring, sampling, and pdb parsing methods are implemented as part of the BioChemical Library (BCL) that is currently being developed in the Meiler laboratory (www.meilerlab.org). BCL::Score is freely available for academic use along with several other components of the BCL library. Details and sample command lines can be found in Appendix S1.

## Supporting Information

Figure S1 **Contact order vs. chain length.**
(DOCX)

Figure S2 **Square radius of gyration vs. chain length.**
(DOCX)

Figure S3 **Minimal distances between amino acid pairs.**
(DOCX)

Figure S4 **Maximal loop length extension.**
(DOCX)

Figure S5 **Illustration of enrichment.**
(DOCX)

Table S1 **Cross validated average enrichments for individual protein models sets and different quality criteria.**
(DOCX)

Appendix S1 **BCL::Score availability and its usage.**
(DOCX)
