# Supplementary material for: BCL::Score—Knowledge Based Energy Potentials for Ranking Protein Models Represented by Idealized Secondary Structure Elements
Source: PLoS One. 2012 Nov 16;7(11):e49242. doi: 10.1371/journal.pone.0049242 (PMC3500277; doi:10.1371/journal.pone.0049242)
Supplement: Figure S1 — Contact order vs. chain length. (DOCX) [file pone.0049242.s001.docx]

A contact order potential was developed to discriminate against protein models with high complexitiy. If the connectivity is omitted by assembling secondary structure elements without their explicit loop connectivity, it is possible to bring secondary structure elements of high sequence distance close in space, resulting in an overall high contact order. Hence, one can observe the contact order as a function of sequence length (number of residues). Proteins with a low contact order relative to the sequence length are not considered, since they could arise from proteins with long loops not contacting the protein, but instead contacting neighboring proteins in the crystal structure.


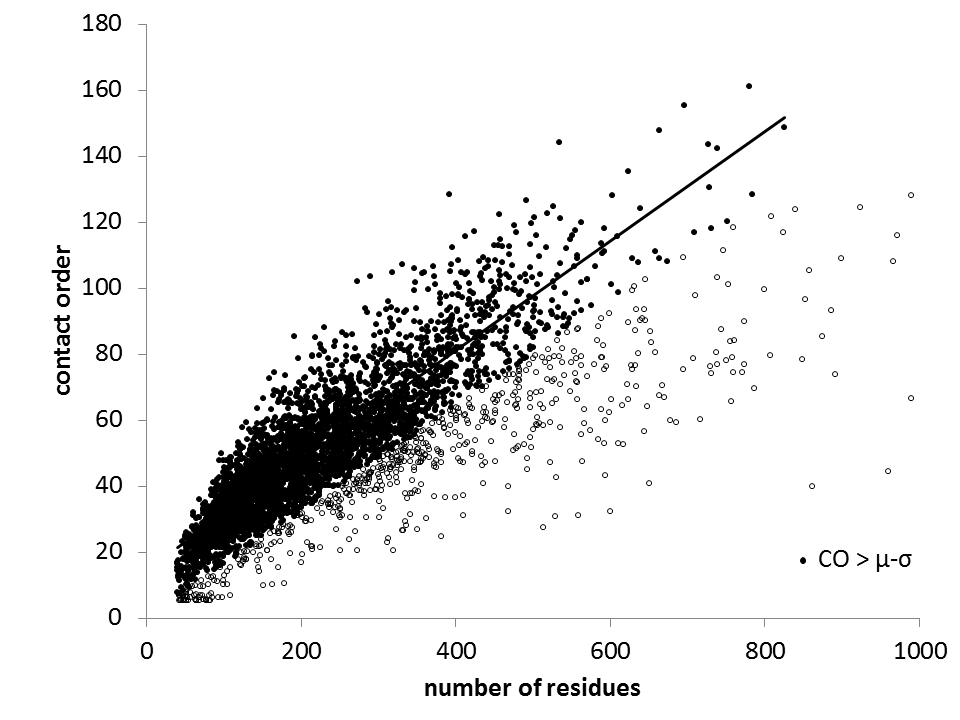


Figure S1 Contact order vs. chain length

Plotted is the amino acid chain contact order of 4303 protein chains. Empty circles have a ratio below the 86% statistical confidence interval and are not considered for the potential (475 chains). The filled circles with the linear fit line are CO/length ratios that are considered for the potential.
