# Supplementary material for: BCL::Score—Knowledge Based Energy Potentials for Ranking Protein Models Represented by Idealized Secondary Structure Elements
Source: PLoS One. 2012 Nov 16;7(11):e49242. doi: 10.1371/journal.pone.0049242 (PMC3500277; doi:10.1371/journal.pone.0049242)
Supplement: Figure S2 — Square radius of gyration vs. chain length. (DOCX) [file pone.0049242.s002.docx]

The radius of gyration is an ideal measure to evaluate the compactness of a polymer chain. It can be used for proteins as well even if the monomeric units have different substituents (side chains). A linear dependency between the square radius of gyration and the sequence length can be derived. A more compact protein has a lower square radius of gyration, unti the polymer chain clashes withit self. A high radius of gyration can be attributed to a non globular protein topology, as it is observed for loops that are not in contact with the structure, or for coil-coil structure that are expected in multimeric or membrane proteins.


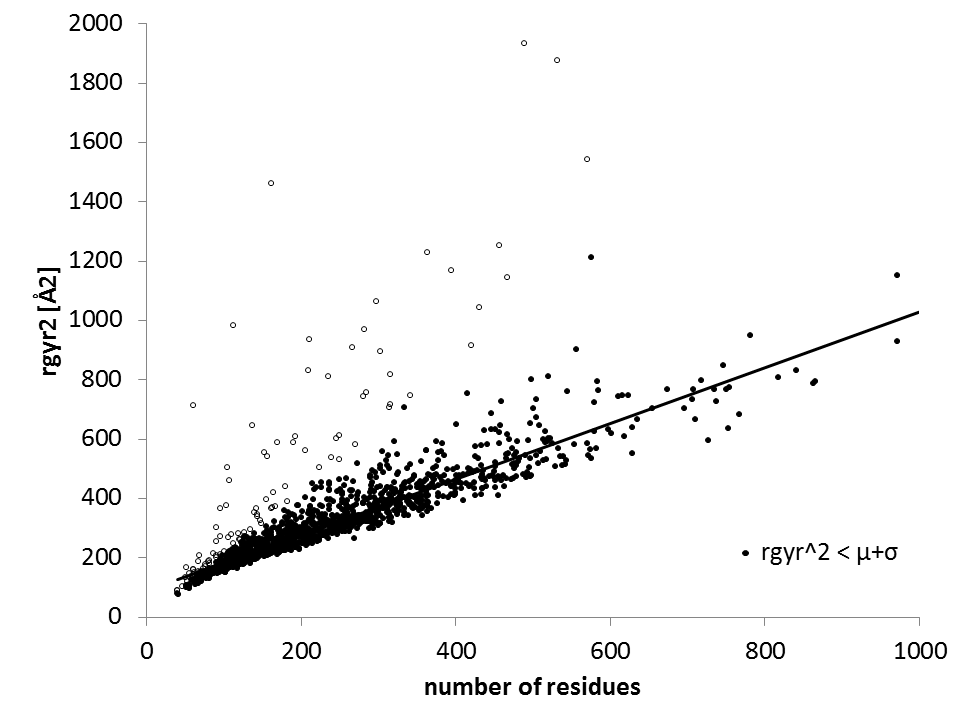


Figure S2 Square radius of gyration vs. chain length

Plotted is the amino acid chain square radius of gyration of 1342 single chain proteins. Empty circles have ratios below the 86% statistical confidence interval and are not considered for the potential (96 proteins). The filled circles with the linear fit line are rgyr^2^/length ratios that are considered for the potential.
