# Supplementary material for: BCL::Score—Knowledge Based Energy Potentials for Ranking Protein Models Represented by Idealized Secondary Structure Elements
Source: PLoS One. 2012 Nov 16;7(11):e49242. doi: 10.1371/journal.pone.0049242 (PMC3500277; doi:10.1371/journal.pone.0049242)
Supplement: Figure S3 — Minimal distances between amino acid pairs. (DOCX) [file pone.0049242.s003.docx]

In the reduced amino acid representation used in this manuscript, clashes between a pair of amino acids cannot be detected directly, since only C_β_ (H_α2_ for Glycine) are considered. Since the side chains are implicitly present in the databank of known protein structures, an absolute minimal distance between the first side chain atoms can be derived. Altough, this distance might not be very common, it is a hard limit below which it will never be possible to place two amino acid side chains in the gap between two amino acids.


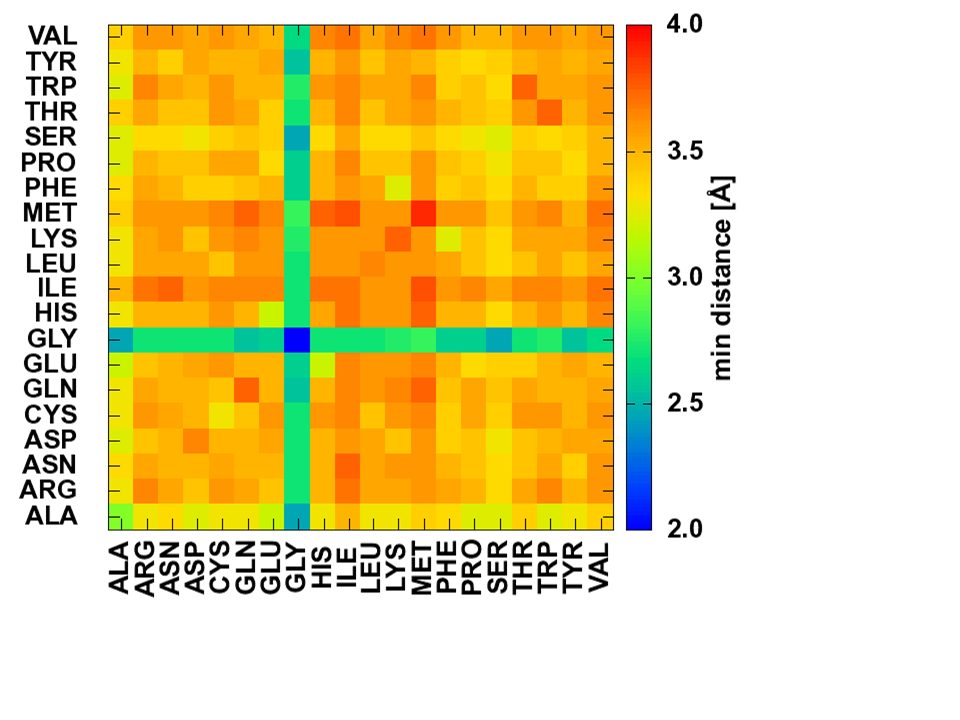


Figure S3 Minimal distances between amino acid pairs

The minimal distances determined by C_β_ atom distance or HA2 for GLY. The distances are color coded. Shorter distances like for Glycine are blue and green, little longer distances like for Alanine are yellow, while long distances go up to red.
