# Supplementary material for: BCL::Score—Knowledge Based Energy Potentials for Ranking Protein Models Represented by Idealized Secondary Structure Elements
Source: PLoS One. 2012 Nov 16;7(11):e49242. doi: 10.1371/journal.pone.0049242 (PMC3500277; doi:10.1371/journal.pone.0049242)
Supplement: Figure S4 — Maximal loop length extension. (DOCX) [file pone.0049242.s004.docx]

Assembling secondary structure elements without their connecting loops being present explcitily can lead to topologies in which the secondary structure elements cannot be connected. The maximal distance between two secondary structure elements, is a function of the number of amino acids in the loop. The rotation and amino acid composition of the loop is disregarded for this distance, but could have an impact as well. Theoretically, if one knows that maximal extend of a single amino acid, this linear function is trivial. But since the exact loop-SSE connection points are unknown, the maximal distance as a function of the length of the loop can be derived from the protein databank. The slope corresponds to a single amino acid extent, and the y-intersect can be interpreted as the C-N distance plus a safety margin that prevents the secondary structure elements from clashing for very short loops. Due to a few extreme geometries in some loops, 5% of the closest loops for each loop length are disregarded.


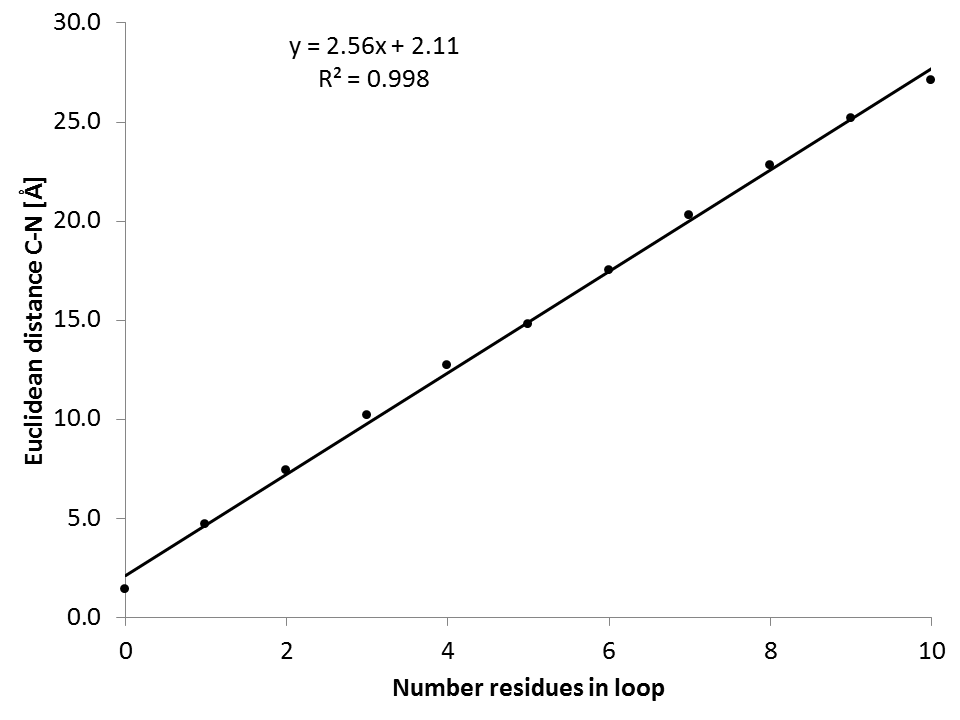


Figure S4 Maximal loop length extension

95% of the longest loop extensions as distance between the backbone carbon and nitrogen atoms vs. the number of residues in the loop. A linear fit shows the trend and can be used to estimate possible loop bridging distances.
