# Supplementary material for: BCL::Score—Knowledge Based Energy Potentials for Ranking Protein Models Represented by Idealized Secondary Structure Elements
Source: PLoS One. 2012 Nov 16;7(11):e49242. doi: 10.1371/journal.pone.0049242 (PMC3500277; doi:10.1371/journal.pone.0049242)
Supplement: Figure S5 — Illustration of enrichment. (DOCX) [file pone.0049242.s005.docx]

To measure how well an energy potential performs, the enrichment is one of the most trusted measures. It measures how many “correct” models are good in energy, relative to a random selection of models. This factor is the enrichment. The difficulty lies within the definition of “correct”. We observed that most incorrect protein topologies had an RMSD100 above 8Å, meaning that at least one SSE was incorrectly placed or flipped in comparison to the PDB structure. Hence, we choose this cutoff since we are interested in selecting proteins of correct topology.


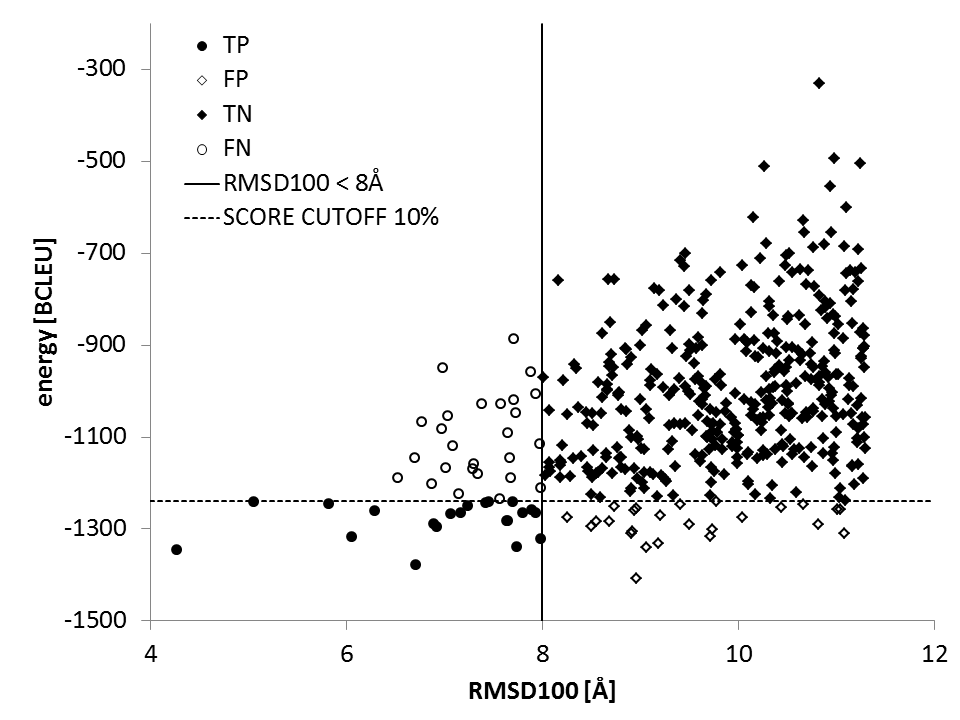


Figure S5 Illustration of enrichment

All protein models are plotted with their quality measure (RMSD100 [Å]) against the energy from the knowledge based potential. 10% models are considered native like if they are e.g left to a given cutoff for RMSD100 < 8Å (solid vertical line, true positive (TP, filled circles) and false negative (FN, empty circles)). Models are considered classified as native like, if they are within the best 10% by energy (dashed vertical line, true positives (TP, filled circles) and false positives (FP, empty squares)). The enrichment is better the larger the number of true negatives (TN, filled squares) and TP and the smaller the number of FP and FN.
