# Supplementary material for: BCL::Score—Knowledge Based Energy Potentials for Ranking Protein Models Represented by Idealized Secondary Structure Elements
Source: PLoS One. 2012 Nov 16;7(11):e49242. doi: 10.1371/journal.pone.0049242 (PMC3500277; doi:10.1371/journal.pone.0049242)
Supplement: Table S1 — Cross validated average enrichments for individual protein models sets and different quality criteria. (DOCX) [file pone.0049242.s006.docx]

| **PDB ID** | **#AAs** | **Class** | **#models Criteria met** | **native like [%]** | **amino acid clash** | | **amino acid distance** | | **amino acid neighbor count** | | **contact order** | | **loop length** | | **loop closure** | | **radius of gyration** | | **SSE clash** | | **SSE packing** | | **strand  pairing** | | **SSPred  JUFO** | | **SSPred PSIPRED** | | **sum** | |
| --- | --- | --- | --- | --- | --- | --- | --- | --- | --- | --- | --- | --- | --- | --- | --- | --- | --- | --- | --- | --- | --- | --- | --- | --- | --- | --- | --- | --- | --- | --- |
| **RMSD100 < 8Å rosetta** | | | | | **avg** | **sd** | **avg** | **sd** | **avg** | **sd** | **avg** | **sd** | **avg** | **sd** | **avg** | **sd** | **avg** | **sd** | **avg** | **sd** | **avg** | **sd** | **avg** | **sd** | **avg** | **sd** | **avg** | **sd** | **avg** | **sd** |
| **1BJ7A** | **156** | **αβ** | **494** | 4.9 | 1.0 | 0.0 | 0.7 | 0.1 | 1.7 | 0.2 | 0.8 | 0.1 | 0.9 | 0.1 | 1.0 | 0.0 | 4.4 | 0.7 | 1.0 | 0.0 | 3.1 | 0.8 | 1.7 | 0.3 | 2.3 | 0.3 | 2.4 | 0.4 | 3.9 | 0.6 |
| **1BZ4A** | **144** | **α** | **760** | 7.6 | 1.2 | 0.0 | 2.1 | 0.0 | 1.2 | 0.0 | 1.3 | 0.0 | 0.1 | 0.0 | 1.0 | 0.0 | 0.0 | 0.0 | 1.6 | 0.0 | 3.4 | 0.2 | 1.0 | 0.0 | 2.2 | 0.1 | 2.2 | 0.1 | 0.3 | 0.0 |
| **1EYHA** | **144** | **α** | **224** | 2.2 | 1.1 | 0.1 | 1.2 | 0.1 | 1.4 | 0.1 | 0.3 | 0.1 | 0.9 | 0.0 | 0.9 | 0.0 | 2.8 | 0.6 | 0.9 | 0.0 | 2.5 | 0.4 | 1.2 | 0.1 | 1.4 | 0.1 | 1.1 | 0.1 | 2.0 | 0.4 |
| **1GAKA** | **141** | **α** | **350** | 3.5 | 1.4 | 0.1 | 2.1 | 0.1 | 0.7 | 0.2 | 2.4 | 0.6 | 0.1 | 0.0 | 1.0 | 0.0 | 0.0 | 0.0 | 1.1 | 0.0 | 4.6 | 0.8 | 1.0 | 0.0 | 3.1 | 0.5 | 0.7 | 0.1 | 0.4 | 0.1 |
| **1GS9A** | **165** | **α** | **409** | 4.1 | 1.3 | 0.1 | 1.7 | 0.2 | 1.4 | 0.0 | 1.5 | 0.1 | 0.1 | 0.1 | 1.0 | 0.0 | 0.3 | 0.4 | 1.4 | 0.1 | 4.1 | 0.3 | 1.0 | 0.0 | 1.7 | 0.2 | 1.8 | 0.3 | 1.8 | 1.1 |
| **1HYPA** | **80** | **α** | **1634** | 16.3 | 1.7 | 0.2 | 1.9 | 0.3 | 1.7 | 0.3 | 1.0 | 0.1 | 0.6 | 0.1 | 1.0 | 0.0 | 3.1 | 0.5 | 1.1 | 0.0 | 1.4 | 0.1 | 1.0 | 0.0 | 0.9 | 0.1 | 0.8 | 0.1 | 3.8 | 0.7 |
| **1ICXA** | **155** | **αβ** | **126** | 1.3 | 0.9 | 0.1 | 1.0 | 0.2 | 1.3 | 0.3 | 1.4 | 0.4 | 1.3 | 0.1 | 1.0 | 0.1 | 3.7 | 1.2 | 1.0 | 0.0 | 3.1 | 0.8 | 2.7 | 0.6 | 2.6 | 0.6 | 2.0 | 0.5 | 5.0 | 1.3 |
| **1IFBA** | **131** | **β** | **2880** | 28.8 | 1.0 | 0.0 | 0.7 | 0.2 | 0.5 | 0.2 | 3.0 | 0.6 | 0.9 | 0.2 | 1.1 | 0.0 | 3.0 | 0.8 | 1.0 | 0.0 | 1.1 | 0.1 | 0.9 | 0.1 | 2.0 | 0.3 | 1.4 | 0.3 | 4.1 | 0.6 |
| **1J27A** | **102** | **β** | **508** | 5.1 | 1.0 | 0.1 | 1.0 | 0.0 | 1.1 | 0.0 | 1.2 | 0.1 | 0.5 | 0.0 | 0.9 | 0.0 | 0.4 | 0.0 | 1.0 | 0.0 | 1.8 | 0.1 | 1.1 | 0.0 | 1.3 | 0.1 | 0.9 | 0.0 | 0.7 | 0.0 |
| **1K6KA** | **143** | **α** | **156** | 1.6 | 1.0 | 0.0 | 1.2 | 0.0 | 1.1 | 0.1 | 1.6 | 0.4 | 1.3 | 0.1 | 1.0 | 0.0 | 2.8 | 0.9 | 0.9 | 0.0 | 1.6 | 0.3 | 1.0 | 0.0 | 1.0 | 0.1 | 1.0 | 0.1 | 2.2 | 0.6 |
| **1M5IA** | **125** | **α** | **625** | 6.3 | 2.2 | 0.1 | 3.3 | 0.1 | 0.9 | 0.2 | 0.2 | 0.0 | 0.0 | 0.0 | 1.0 | 0.0 | 0.0 | 0.0 | 1.5 | 0.0 | 1.8 | 0.3 | 1.0 | 0.0 | 3.5 | 0.1 | 2.9 | 0.0 | 0.0 | 0.0 |
| **1NFNA** | **191** | **α** | **465** | 4.7 | 0.8 | 0.0 | 1.0 | 0.0 | 1.0 | 0.0 | 1.0 | 0.1 | 0.3 | 0.1 | 1.0 | 0.0 | 0.6 | 0.2 | 1.2 | 0.0 | 2.1 | 0.2 | 1.0 | 0.0 | 1.3 | 0.1 | 1.4 | 0.2 | 1.3 | 0.3 |
| **1OZ9A** | **150** | **αβ** | **102** | 1.0 | 1.2 | 0.2 | 1.6 | 0.1 | 1.5 | 0.2 | 0.5 | 0.2 | 0.7 | 0.1 | 1.0 | 0.0 | 2.3 | 1.0 | 1.0 | 0.0 | 2.6 | 0.2 | 0.7 | 0.1 | 1.2 | 0.1 | 1.2 | 0.1 | 2.5 | 0.5 |
| **1TZVA** | **142** | **α** | **1684** | 16.8 | 1.0 | 0.0 | 1.6 | 0.0 | 2.1 | 0.2 | 0.1 | 0.0 | 0.9 | 0.0 | 1.0 | 0.0 | 1.9 | 0.4 | 1.0 | 0.0 | 3.3 | 0.2 | 1.0 | 0.0 | 1.5 | 0.1 | 1.0 | 0.1 | 2.6 | 0.5 |
| **1UBIA** | **76** | **β** | **122** | 1.2 | 1.0 | 0.1 | 1.7 | 0.4 | 1.5 | 0.6 | 1.2 | 0.1 | 0.9 | 0.0 | 1.0 | 0.0 | 1.7 | 0.7 | 1.0 | 0.0 | 1.9 | 0.8 | 2.6 | 0.1 | 2.6 | 0.3 | 1.1 | 0.2 | 2.1 | 0.8 |
| **1X91A** | **153** | **α** | **3462** | 34.6 | 0.9 | 0.1 | 2.1 | 0.5 | 1.9 | 0.5 | 1.3 | 0.1 | 0.2 | 0.1 | 1.0 | 0.0 | 2.7 | 0.5 | 1.2 | 0.1 | 5.4 | 1.4 | 1.0 | 0.0 | 2.3 | 0.4 | 1.3 | 0.2 | 4.9 | 1.3 |
| **1XGWA** | **176** | **α** | **130** | 1.3 | 0.7 | 0.1 | 0.7 | 0.1 | 0.6 | 0.1 | 0.2 | 0.2 | 1.2 | 0.2 | 1.0 | 0.0 | 2.1 | 0.6 | 0.9 | 0.0 | 2.1 | 0.6 | 1.0 | 0.1 | 0.8 | 0.1 | 0.8 | 0.1 | 1.2 | 0.3 |
| **2CWYA** | **94** | **α** | **2018** | 20.2 | 1.2 | 0.1 | 1.4 | 0.2 | 1.3 | 0.2 | 0.5 | 0.1 | 1.7 | 0.2 | 1.0 | 0.0 | 1.4 | 0.1 | 1.0 | 0.0 | 2.4 | 0.3 | 1.0 | 0.0 | 0.8 | 0.1 | 0.7 | 0.1 | 1.8 | 0.1 |
| **RMSD100 < 8Å bcl_perturb** | | | | |  |  |  |  |  |  |  |  |  |  |  |  |  |  |  |  |  |  |  |  |  |  |  |  |  |  |
| **1AAJA** | **105** | **β** | **938** | 7.8 | 2.2 | 0.1 | 2.5 | 0.1 | 3.2 | 0.2 | 1.0 | 0.0 | 4.6 | 0.2 | 3.2 | 0.1 | 1.5 | 0.1 | 2.2 | 0.2 | 3.1 | 0.2 | 3.6 | 0.3 | 0.7 | 0.0 | 0.7 | 0.0 | 3.7 | 0.3 |
| **1BGCA** | **174** | **α** | **1959** | 16.3 | 1.7 | 0.4 | 1.8 | 0.5 | 0.9 | 0.1 | 0.7 | 0.1 | 0.0 | 0.0 | 1.5 | 0.0 | 0.9 | 0.1 | 1.0 | 0.0 | 0.7 | 0.1 | 1.0 | 0.0 | 0.6 | 0.1 | 0.6 | 0.1 | 0.7 | 0.2 |
| **1BJ7A** | **156** | **αβ** | **1130** | 9.4 | 2.7 | 0.0 | 2.7 | 0.0 | 3.3 | 0.1 | 0.1 | 0.0 | 6.1 | 0.1 | 4.8 | 0.1 | 1.0 | 0.0 | 2.1 | 0.0 | 3.4 | 0.1 | 3.6 | 0.1 | 1.1 | 0.0 | 1.1 | 0.0 | 2.2 | 0.0 |
| **1BZ4A** | **144** | **α** | **1630** | 13.6 | 2.0 | 0.2 | 2.2 | 0.3 | 1.4 | 0.2 | 1.0 | 0.0 | 3.1 | 0.1 | 3.6 | 0.1 | 2.1 | 0.1 | 1.1 | 0.0 | 0.6 | 0.0 | 1.0 | 0.0 | 0.5 | 0.0 | 0.5 | 0.0 | 0.7 | 0.0 |
| **1CHDA** | **203** | **αβ** | **3839** | 32.0 | 2.7 | 0.9 | 3.0 | 1.0 | 1.6 | 0.2 | 0.1 | 0.1 | 5.0 | 1.5 | 3.7 | 1.2 | 0.9 | 0.1 | 2.0 | 0.3 | 3.6 | 1.2 | 2.9 | 0.7 | 1.1 | 0.0 | 1.1 | 0.0 | 1.4 | 0.1 |
| **1DUSA** | **194** | **αβ** | **2227** | 18.6 | 3.0 | 0.4 | 3.2 | 0.4 | 3.7 | 0.6 | 0.2 | 0.1 | 5.8 | 0.7 | 4.5 | 0.8 | 1.6 | 0.1 | 1.6 | 0.1 | 3.9 | 0.6 | 3.3 | 0.3 | 1.1 | 0.0 | 1.1 | 0.0 | 3.1 | 0.3 |
| **1EYHA** | **144** | **α** | **814** | 6.8 | 1.8 | 0.1 | 2.0 | 0.1 | 2.9 | 0.2 | 0.4 | 0.0 | 5.6 | 0.3 | 4.0 | 0.2 | 1.2 | 0.0 | 1.1 | 0.0 | 1.8 | 0.0 | 1.0 | 0.0 | 1.0 | 0.0 | 1.0 | 0.0 | 1.8 | 0.1 |
| **1G8AA** | **227** | **αβ** | **3062** | 25.5 | 3.2 | 0.5 | 3.4 | 0.6 | 2.8 | 0.4 | 0.1 | 0.1 | 5.8 | 1.1 | 5.0 | 1.3 | 0.4 | 0.1 | 1.8 | 0.1 | 4.6 | 1.0 | 2.9 | 0.4 | 1.2 | 0.0 | 1.0 | 0.0 | 2.0 | 0.4 |
| **1GAKA** | **141** | **α** | **621** | 5.2 | 2.0 | 0.1 | 2.3 | 0.1 | 3.3 | 0.2 | 1.3 | 0.1 | 3.1 | 0.1 | 3.6 | 0.3 | 1.9 | 0.6 | 1.1 | 0.0 | 1.9 | 0.1 | 1.0 | 0.0 | 0.9 | 0.1 | 1.0 | 0.0 | 3.0 | 0.2 |
| **1GCUA** | **295** | **αβ** | **1944** | 16.2 | 3.3 | 0.2 | 3.7 | 0.3 | 4.0 | 0.4 | 0.1 | 0.0 | 5.3 | 0.5 | 4.9 | 0.6 | 0.3 | 0.1 | 1.6 | 0.0 | 3.7 | 0.3 | 3.3 | 0.1 | 1.1 | 0.0 | 1.1 | 0.0 | 2.0 | 0.0 |
| **1GS9A** | **165** | **α** | **688** | 5.7 | 2.3 | 0.1 | 2.5 | 0.1 | 2.5 | 0.1 | 0.7 | 0.0 | 4.4 | 0.4 | 4.8 | 0.6 | 2.6 | 0.3 | 1.2 | 0.0 | 2.0 | 0.1 | 1.0 | 0.0 | 0.6 | 0.0 | 0.6 | 0.0 | 2.1 | 0.0 |
| **1HYPA** | **80** | **α** | **848** | 7.1 | 2.1 | 0.1 | 2.3 | 0.1 | 1.5 | 0.0 | 0.4 | 0.0 | 2.9 | 0.2 | 2.3 | 0.3 | 1.2 | 0.0 | 1.1 | 0.0 | 1.1 | 0.1 | 1.0 | 0.0 | 1.8 | 0.2 | 2.0 | 0.2 | 1.4 | 0.0 |
| **1IAPA** | **211** | **α** | **1008** | 8.4 | 1.7 | 0.0 | 2.2 | 0.0 | 3.0 | 0.1 | 1.3 | 0.0 | 5.7 | 0.3 | 5.2 | 0.2 | 1.5 | 0.1 | 1.0 | 0.0 | 2.0 | 0.1 | 1.0 | 0.0 | 1.0 | 0.0 | 1.0 | 0.0 | 3.0 | 0.1 |
| **1ICXA** | **155** | **αβ** | **832** | 6.9 | 3.3 | 0.3 | 3.3 | 0.3 | 3.6 | 0.3 | 0.9 | 0.0 | 6.8 | 0.6 | 5.5 | 0.4 | 1.5 | 0.1 | 2.5 | 0.3 | 5.0 | 0.6 | 3.8 | 0.4 | 1.2 | 0.0 | 1.1 | 0.0 | 5.1 | 0.6 |
| **1IFBA** | **131** | **β** | **1061** | 8.8 | 3.4 | 0.2 | 3.4 | 0.2 | 2.9 | 0.2 | 0.4 | 0.0 | 7.1 | 0.4 | 6.8 | 0.3 | 1.5 | 0.1 | 1.4 | 0.1 | 4.2 | 0.3 | 3.5 | 0.3 | 1.1 | 0.0 | 1.1 | 0.0 | 4.2 | 0.3 |
| **1J27A** | **102** | **β** | **385** | 3.2 | 2.3 | 0.3 | 2.4 | 0.3 | 2.6 | 0.2 | 0.7 | 0.0 | 5.4 | 0.3 | 4.7 | 0.5 | 1.2 | 0.1 | 1.8 | 0.2 | 3.5 | 0.3 | 2.4 | 0.2 | 0.9 | 0.1 | 0.9 | 0.1 | 3.1 | 0.3 |
| **1JL1A** | **155** | **αβ** | **1223** | 10.2 | 2.4 | 0.0 | 2.6 | 0.0 | 3.0 | 0.0 | 1.3 | 0.0 | 4.6 | 0.0 | 3.2 | 0.0 | 0.2 | 0.0 | 1.2 | 0.0 | 3.4 | 0.0 | 3.1 | 0.0 | 1.0 | 0.0 | 1.0 | 0.0 | 3.4 | 0.0 |
| **1K6KA** | **143** | **α** | **1123** | 9.4 | 2.3 | 0.0 | 2.8 | 0.0 | 1.7 | 0.0 | 1.5 | 0.0 | 3.8 | 0.1 | 3.5 | 0.0 | 1.2 | 0.0 | 1.4 | 0.0 | 2.2 | 0.0 | 1.0 | 0.0 | 1.1 | 0.0 | 1.1 | 0.0 | 2.9 | 0.0 |
| **1LKFA** | **299** | **αβ** | **1827** | 15.2 | 2.6 | 0.3 | 2.9 | 0.3 | 3.5 | 0.3 | 0.3 | 0.1 | 4.9 | 0.4 | 5.6 | 0.6 | 0.2 | 0.1 | 2.3 | 0.2 | 3.5 | 0.3 | 4.2 | 0.3 | 1.0 | 0.0 | 0.9 | 0.0 | 3.1 | 0.2 |
| **1LKIA** | **180** | **α** | **1786** | 14.9 | 2.1 | 0.4 | 2.2 | 0.5 | 0.9 | 0.1 | 0.7 | 0.1 | 0.2 | 0.1 | 1.9 | 0.1 | 0.7 | 0.0 | 1.0 | 0.0 | 0.8 | 0.1 | 1.0 | 0.0 | 0.5 | 0.1 | 0.5 | 0.1 | 0.7 | 0.1 |
| **1LWBA** | **122** | **α** | **596** | 5.0 | 2.6 | 0.1 | 2.8 | 0.2 | 1.3 | 0.1 | 0.7 | 0.0 | 3.7 | 0.3 | 4.5 | 0.5 | 1.1 | 0.1 | 1.3 | 0.0 | 1.6 | 0.1 | 1.0 | 0.0 | 0.6 | 0.0 | 1.2 | 0.1 | 1.4 | 0.0 |
| **1M5IA** | **125** | **α** | **1725** | 14.4 | 1.8 | 0.2 | 0.8 | 0.2 | 1.2 | 0.1 | 0.3 | 0.1 | 3.5 | 0.3 | 4.5 | 0.2 | 3.4 | 0.9 | 1.1 | 0.0 | 0.7 | 0.1 | 1.0 | 0.0 | 0.4 | 0.1 | 0.4 | 0.1 | 0.5 | 0.1 |
| **1NFNA** | **191** | **α** | **2249** | 18.7 | 1.8 | 0.3 | 1.9 | 0.4 | 1.4 | 0.2 | 0.6 | 0.1 | 3.5 | 0.2 | 3.3 | 0.0 | 1.1 | 0.1 | 1.0 | 0.0 | 0.7 | 0.0 | 1.0 | 0.0 | 0.6 | 0.1 | 0.6 | 0.1 | 0.7 | 0.0 |
| **1OA9A** | **214** | **αβ** | **6175** | 51.5 | 1.5 | 0.3 | 1.6 | 0.4 | 2.5 | 0.8 | 1.2 | 0.1 | 1.7 | 0.7 | 1.0 | 0.1 | 0.8 | 0.0 | 1.7 | 0.4 | 2.9 | 0.9 | 1.9 | 0.6 | 1.1 | 0.1 | 1.5 | 0.2 | 2.6 | 0.9 |
| **1OZ9A** | **150** | **αβ** | **1089** | 9.1 | 3.2 | 0.1 | 3.7 | 0.1 | 4.7 | 0.2 | 0.7 | 0.0 | 5.0 | 0.1 | 3.3 | 0.1 | 0.7 | 0.0 | 1.2 | 0.0 | 3.9 | 0.1 | 3.2 | 0.1 | 1.1 | 0.0 | 0.9 | 0.0 | 4.1 | 0.1 |
| **1PRZA** | **252** | **αβ** | **2209** | 18.4 | 3.0 | 0.6 | 3.1 | 0.6 | 1.7 | 0.1 | 0.1 | 0.0 | 4.9 | 0.7 | 3.4 | 0.6 | 0.6 | 0.1 | 2.1 | 0.2 | 3.3 | 0.6 | 2.7 | 0.3 | 1.0 | 0.0 | 0.9 | 0.1 | 2.1 | 0.2 |
| **1ROAA** | **122** | **β** | **653** | 5.4 | 2.8 | 0.2 | 2.9 | 0.2 | 1.8 | 0.1 | 0.7 | 0.0 | 3.9 | 0.5 | 4.7 | 0.9 | 1.8 | 0.2 | 2.6 | 0.2 | 2.8 | 0.1 | 1.7 | 0.1 | 0.7 | 0.1 | 0.9 | 0.0 | 2.5 | 0.1 |
| **1TZVA** | **142** | **α** | **681** | 5.7 | 1.8 | 0.1 | 2.6 | 0.1 | 4.3 | 0.4 | 0.2 | 0.0 | 6.1 | 0.6 | 4.2 | 0.4 | 1.4 | 0.1 | 1.0 | 0.0 | 1.8 | 0.1 | 1.0 | 0.0 | 1.0 | 0.0 | 1.0 | 0.0 | 2.1 | 0.1 |
| **1UBIA** | **76** | **β** | **802** | 6.7 | 2.4 | 0.1 | 2.4 | 0.2 | 1.7 | 0.1 | 1.1 | 0.0 | 2.6 | 0.1 | 2.1 | 0.2 | 1.0 | 0.1 | 1.7 | 0.0 | 2.1 | 0.0 | 1.5 | 0.0 | 0.7 | 0.0 | 0.7 | 0.0 | 2.5 | 0.1 |
| **1UEKA** | **275** | **αβ** | **1887** | 15.7 | 3.3 | 0.4 | 3.7 | 0.4 | 3.3 | 0.3 | 0.2 | 0.1 | 5.6 | 0.4 | 5.0 | 0.5 | 1.6 | 0.1 | 2.9 | 0.3 | 3.8 | 0.4 | 3.7 | 0.3 | 1.1 | 0.0 | 1.0 | 0.0 | 3.3 | 0.2 |
| **1VGJA** | **184** | **αβ** | **1409** | 11.7 | 4.1 | 0.1 | 4.4 | 0.2 | 4.1 | 0.2 | 1.1 | 0.0 | 4.6 | 0.2 | 4.6 | 0.3 | 0.4 | 0.0 | 3.0 | 0.1 | 4.7 | 0.2 | 3.4 | 0.1 | 1.0 | 0.0 | 0.9 | 0.0 | 5.1 | 0.2 |
| **1VK4A** | **298** | **αβ** | **4155** | 34.6 | 3.2 | 0.7 | 3.4 | 0.8 | 1.3 | 0.1 | 0.2 | 0.2 | 5.5 | 1.4 | 5.0 | 1.6 | 0.2 | 0.1 | 1.1 | 0.1 | 3.9 | 1.0 | 3.3 | 0.5 | 1.1 | 0.0 | 1.1 | 0.0 | 0.7 | 0.3 |
| **1WBAA** | **175** | **β** | **2707** | 22.6 | 2.0 | 0.2 | 2.2 | 0.2 | 2.3 | 0.3 | 0.3 | 0.1 | 3.2 | 0.8 | 2.0 | 0.3 | 0.3 | 0.0 | 1.6 | 0.1 | 2.1 | 0.2 | 2.7 | 0.3 | 1.1 | 0.0 | 1.1 | 0.0 | 0.4 | 0.1 |
| **1WNHA** | **225** | **αβ** | **1349** | 11.2 | 3.6 | 0.1 | 3.8 | 0.1 | 3.1 | 0.1 | 0.0 | 0.0 | 5.6 | 0.1 | 5.4 | 0.2 | 1.6 | 0.0 | 2.8 | 0.1 | 4.4 | 0.1 | 3.7 | 0.1 | 1.1 | 0.0 | 0.9 | 0.0 | 2.6 | 0.0 |
| **1WR2A** | **238** | **αβ** | **2074** | 17.3 | 3.1 | 0.4 | 3.6 | 0.5 | 4.1 | 0.5 | 0.5 | 0.0 | 5.3 | 0.6 | 4.4 | 0.6 | 0.4 | 0.1 | 1.9 | 0.1 | 3.5 | 0.5 | 2.9 | 0.3 | 1.1 | 0.0 | 1.1 | 0.0 | 3.4 | 0.3 |
| **1WVHA** | **134** | **β** | **1201** | 10.0 | 3.2 | 0.0 | 3.5 | 0.0 | 1.3 | 0.0 | 2.0 | 0.0 | 4.0 | 0.0 | 2.9 | 0.0 | 0.4 | 0.0 | 2.7 | 0.0 | 4.1 | 0.0 | 3.6 | 0.0 | 0.9 | 0.0 | 0.9 | 0.0 | 4.2 | 0.0 |
| **1X91A** | **153** | **α** | **661** | 5.5 | 2.2 | 0.2 | 2.3 | 0.1 | 2.0 | 0.1 | 0.8 | 0.0 | 4.5 | 0.3 | 4.7 | 0.5 | 2.5 | 0.2 | 1.1 | 0.0 | 1.6 | 0.0 | 1.0 | 0.0 | 0.6 | 0.0 | 1.0 | 0.0 | 1.5 | 0.0 |
| **1XGWA** | **176** | **α** | **1517** | 12.6 | 1.3 | 0.0 | 1.6 | 0.1 | 3.5 | 0.3 | 0.1 | 0.0 | 4.5 | 0.4 | 3.1 | 0.3 | 0.4 | 0.0 | 1.1 | 0.0 | 1.3 | 0.0 | 1.0 | 0.0 | 1.0 | 0.0 | 1.0 | 0.0 | 0.2 | 0.0 |
| **1XKRA** | **206** | **αβ** | **1395** | 11.6 | 2.5 | 0.1 | 3.2 | 0.2 | 1.9 | 0.1 | 1.1 | 0.0 | 3.1 | 0.2 | 3.0 | 0.3 | 0.7 | 0.0 | 2.2 | 0.0 | 3.5 | 0.1 | 2.6 | 0.1 | 1.1 | 0.0 | 1.0 | 0.0 | 4.1 | 0.1 |
| **1XQOA** | **256** | **α** | **1857** | 15.5 | 1.8 | 0.1 | 2.6 | 0.2 | 2.9 | 0.3 | 0.4 | 0.1 | 5.3 | 0.5 | 4.6 | 0.5 | 0.7 | 0.1 | 1.2 | 0.0 | 1.7 | 0.1 | 1.0 | 0.0 | 0.9 | 0.0 | 0.9 | 0.0 | 2.0 | 0.1 |
| **2CWYA** | **94** | **α** | **809** | 6.7 | 2.2 | 0.1 | 2.3 | 0.1 | 2.7 | 0.1 | 0.5 | 0.0 | 3.7 | 0.2 | 3.6 | 0.3 | 2.0 | 0.3 | 1.1 | 0.0 | 1.2 | 0.0 | 1.0 | 0.0 | 0.7 | 0.0 | 0.7 | 0.0 | 1.1 | 0.0 |
| **2E3SA** | **255** | **αβ** | **2027** | 16.9 | 3.1 | 0.3 | 3.4 | 0.3 | 3.7 | 0.6 | 0.3 | 0.1 | 4.4 | 0.7 | 3.9 | 0.7 | 0.3 | 0.0 | 2.3 | 0.1 | 3.3 | 0.4 | 3.4 | 0.3 | 1.2 | 0.0 | 1.2 | 0.0 | 2.5 | 0.1 |
| **2EJXA** | **139** | **αβ** | **1142** | 9.5 | 3.1 | 0.1 | 3.6 | 0.1 | 2.8 | 0.0 | 0.6 | 0.0 | 5.7 | 0.1 | 4.7 | 0.0 | 1.7 | 0.0 | 2.9 | 0.1 | 4.4 | 0.1 | 3.2 | 0.0 | 1.1 | 0.0 | 1.0 | 0.0 | 4.8 | 0.1 |
| **2FM9A** | **215** | **α** | **768** | 6.4 | 2.3 | 0.1 | 3.0 | 0.2 | 2.2 | 0.1 | 0.5 | 0.0 | 6.7 | 0.6 | 4.1 | 0.3 | 1.4 | 0.2 | 1.1 | 0.0 | 2.5 | 0.3 | 1.0 | 0.0 | 1.2 | 0.0 | 1.2 | 0.0 | 2.9 | 0.3 |
| **2ILRA** | **264** | **α** | **1724** | 14.4 | 1.8 | 0.1 | 2.5 | 0.1 | 3.0 | 0.2 | 0.0 | 0.0 | 6.9 | 0.4 | 4.9 | 0.5 | 0.5 | 0.0 | 1.1 | 0.0 | 1.4 | 0.0 | 1.0 | 0.0 | 1.1 | 0.0 | 1.1 | 0.0 | 0.0 | 0.0 |
| **2IU1A** | **208** | **α** | **1631** | 13.6 | 1.7 | 0.1 | 2.1 | 0.1 | 3.0 | 0.3 | 0.3 | 0.0 | 4.5 | 0.4 | 3.6 | 0.3 | 0.8 | 0.0 | 1.1 | 0.0 | 2.6 | 0.1 | 1.0 | 0.0 | 1.0 | 0.0 | 1.0 | 0.0 | 1.7 | 0.1 |
| **2OF3A** | **266** | **α** | **1649** | 13.7 | 1.9 | 0.1 | 2.4 | 0.2 | 3.8 | 0.3 | 0.0 | 0.0 | 6.5 | 0.3 | 5.1 | 0.4 | 0.5 | 0.0 | 1.1 | 0.0 | 2.3 | 0.1 | 1.0 | 0.0 | 1.1 | 0.0 | 1.1 | 0.0 | 0.0 | 0.0 |
| **2OPWA** | **291** | **αβ** | **2668** | 22.2 | 3.0 | 0.5 | 3.3 | 0.7 | 3.1 | 0.7 | 0.9 | 0.2 | 3.0 | 0.7 | 3.2 | 0.9 | 2.5 | 0.3 | 2.1 | 0.3 | 3.8 | 0.6 | 2.8 | 0.4 | 0.9 | 0.0 | 0.8 | 0.0 | 4.0 | 0.7 |
| **2OSAA** | **202** | **α** | **1036** | 8.6 | 1.6 | 0.0 | 2.1 | 0.1 | 4.7 | 0.2 | 0.2 | 0.0 | 5.3 | 0.2 | 4.1 | 0.1 | 1.2 | 0.0 | 1.1 | 0.0 | 1.4 | 0.0 | 1.0 | 0.0 | 1.1 | 0.0 | 0.9 | 0.0 | 1.4 | 0.0 |
| **2YV8A** | **164** | **β** | **2002** | 16.7 | 1.8 | 0.1 | 2.1 | 0.1 | 1.5 | 0.1 | 1.1 | 0.1 | 3.8 | 0.4 | 3.0 | 0.4 | 0.3 | 0.0 | 1.6 | 0.1 | 2.8 | 0.3 | 2.8 | 0.2 | 1.0 | 0.0 | 1.0 | 0.0 | 0.9 | 0.0 |
| **2YVTA** | **260** | **αβ** | **3045** | 25.4 | 3.6 | 0.9 | 3.9 | 1.0 | 3.4 | 0.8 | 0.7 | 0.2 | 4.2 | 0.9 | 4.2 | 1.2 | 2.2 | 0.4 | 2.2 | 0.2 | 4.0 | 1.3 | 2.9 | 0.5 | 1.1 | 0.0 | 1.0 | 0.0 | 3.7 | 0.6 |
| **2ZCOA** | **293** | **α** | **1485** | 12.4 | 2.0 | 0.1 | 2.5 | 0.2 | 3.9 | 0.2 | 0.1 | 0.0 | 5.8 | 0.2 | 5.4 | 0.4 | 0.7 | 0.0 | 1.1 | 0.0 | 3.0 | 0.0 | 1.0 | 0.0 | 1.1 | 0.0 | 1.1 | 0.0 | 0.5 | 0.1 |
| **3B5OA** | **244** | **α** | **736** | 6.1 | 2.0 | 0.1 | 2.4 | 0.2 | 3.8 | 0.4 | 1.3 | 0.0 | 4.2 | 0.3 | 3.7 | 0.3 | 1.0 | 0.0 | 1.3 | 0.0 | 3.1 | 0.3 | 1.0 | 0.0 | 1.1 | 0.0 | 1.0 | 0.0 | 3.3 | 0.3 |
| **RMSD100 < 8Å bcl_fold** | | | | |  |  |  |  |  |  |  |  |  |  |  |  |  |  |  |  |  |  |  |  |  |  |  |  |  |  |
| **1BGCA** | **174** | **α** | **1476** | 14.8 | 1.3 | 0.0 | 1.7 | 0.1 | 0.8 | 0.0 | 0.7 | 0.1 | 0.0 | 0.0 | 1.3 | 0.0 | 0.8 | 0.0 | 1.0 | 0.0 | 1.6 | 0.3 | 1.0 | 0.0 | 1.0 | 0.0 | 1.0 | 0.0 | 1.8 | 0.1 |
| **1BZ4A** | **144** | **α** | **2111** | 21.1 | 1.1 | 0.1 | 1.6 | 0.1 | 1.0 | 0.1 | 1.6 | 0.2 | 1.7 | 0.1 | 1.5 | 0.1 | 0.5 | 0.2 | 1.0 | 0.0 | 1.3 | 0.1 | 1.0 | 0.0 | 1.0 | 0.0 | 1.0 | 0.0 | 1.5 | 0.1 |
| **1HYPA** | **80** | **α** | **204** | 2.0 | 1.2 | 0.1 | 1.3 | 0.1 | 0.6 | 0.1 | 0.7 | 0.2 | 2.5 | 0.5 | 1.3 | 0.1 | 1.0 | 0.1 | 1.0 | 0.0 | 0.8 | 0.1 | 1.0 | 0.0 | 1.3 | 0.0 | 1.3 | 0.0 | 0.7 | 0.1 |
| **1J27A** | **102** | **β** | **1954** | 19.5 | 1.1 | 0.1 | 1.7 | 0.1 | 1.1 | 0.1 | 0.3 | 0.1 | 1.3 | 0.1 | 1.1 | 0.2 | 1.6 | 0.2 | 1.0 | 0.0 | 0.1 | 0.0 | 0.9 | 0.1 | 1.0 | 0.0 | 1.0 | 0.0 | 0.4 | 0.2 |
| **1LKIA** | **180** | **α** | **926** | 9.3 | 1.3 | 0.0 | 1.3 | 0.0 | 0.8 | 0.0 | 1.1 | 0.0 | 0.1 | 0.0 | 1.1 | 0.0 | 1.0 | 0.0 | 1.0 | 0.0 | 2.2 | 0.0 | 1.0 | 0.0 | 1.0 | 0.0 | 1.0 | 0.0 | 2.0 | 0.0 |
| **1M5IA** | **125** | **α** | **974** | 9.7 | 0.6 | 0.0 | 0.5 | 0.0 | 2.9 | 0.1 | 0.1 | 0.0 | 4.0 | 0.0 | 1.8 | 0.0 | 1.0 | 0.0 | 0.9 | 0.0 | 0.0 | 0.0 | 1.0 | 0.0 | 1.0 | 0.0 | 1.0 | 0.0 | 0.0 | 0.0 |
| **1NFNA** | **191** | **α** | **2462** | 24.6 | 0.9 | 0.1 | 1.7 | 0.3 | 1.3 | 0.1 | 0.9 | 0.4 | 1.7 | 0.3 | 1.3 | 0.2 | 1.5 | 0.2 | 1.0 | 0.0 | 1.0 | 0.1 | 1.0 | 0.0 | 1.0 | 0.0 | 1.0 | 0.0 | 1.5 | 0.3 |
| **1ROAA** | **122** | **β** | **720** | 7.2 | 0.7 | 0.0 | 0.9 | 0.0 | 1.9 | 0.1 | 0.7 | 0.0 | 2.1 | 0.2 | 1.7 | 0.1 | 2.3 | 0.3 | 1.0 | 0.0 | 0.9 | 0.0 | 0.7 | 0.0 | 1.0 | 0.0 | 1.0 | 0.0 | 0.7 | 0.1 |
| **1TZVA** | **142** | **α** | **277** | 2.8 | 1.0 | 0.1 | 1.4 | 0.3 | 0.3 | 0.3 | 0.1 | 0.2 | 0.5 | 0.8 | 1.6 | 0.1 | 0.3 | 0.4 | 1.0 | 0.0 | 0.3 | 0.3 | 1.0 | 0.0 | 0.3 | 0.4 | 0.3 | 0.4 | 0.2 | 0.4 |
| **1UBIA** | **76** | **β** | **985** | 9.9 | 1.1 | 0.0 | 0.8 | 0.0 | 0.3 | 0.0 | 3.4 | 0.0 | 4.2 | 0.0 | 1.4 | 0.0 | 0.8 | 0.0 | 1.0 | 0.0 | 0.7 | 0.0 | 0.0 | 0.0 | 1.0 | 0.0 | 1.0 | 0.0 | 4.3 | 0.0 |
| **1X91A** | **153** | **α** | **2191** | 21.9 | 0.9 | 0.0 | 0.9 | 0.2 | 1.4 | 0.3 | 1.2 | 0.1 | 2.1 | 0.4 | 0.7 | 0.2 | 1.2 | 0.0 | 1.0 | 0.0 | 2.2 | 0.4 | 1.0 | 0.0 | 1.1 | 0.0 | 1.1 | 0.0 | 2.1 | 0.4 |
| **1XGWA** | **176** | **α** | **374** | 3.7 | 1.3 | 0.1 | 2.1 | 0.5 | 0.8 | 0.1 | 0.3 | 0.0 | 0.2 | 0.2 | 0.7 | 0.2 | 0.3 | 0.1 | 1.0 | 0.0 | 1.0 | 0.1 | 1.0 | 0.0 | 1.0 | 0.0 | 1.0 | 0.0 | 0.5 | 0.0 |
| **2CWYA** | **94** | **α** | **452** | 4.5 | 3.2 | 0.8 | 0.0 | 0.0 | 0.0 | 0.0 | 0.0 | 0.0 | 0.4 | 0.7 | 1.4 | 0.1 | 0.0 | 0.0 | 1.0 | 0.0 | 0.0 | 0.0 | 1.0 | 0.0 | 0.0 | 0.0 | 0.0 | 0.0 | 0.0 | 0.0 |
| **2EJXA** | **139** | **αβ** | **142** | 1.4 | 1.6 | 0.2 | 1.0 | 0.1 | 0.4 | 0.1 | 0.5 | 0.1 | 3.8 | 0.2 | 2.8 | 0.3 | 1.1 | 0.2 | 1.0 | 0.0 | 0.5 | 0.4 | 1.0 | 0.2 | 0.9 | 0.0 | 0.9 | 0.0 | 0.7 | 0.3 |
| **GDT_TS > 25% rosetta** | | | | |  |  |  |  |  |  |  |  |  |  |  |  |  |  |  |  |  |  |  |  |  |  |  |  |  |  |
| **1AAJA** | **105** | **β** | **2506** | 25.1 | 0.9 | 0.0 | 1.0 | 0.1 | 1.0 | 0.1 | 0.9 | 0.1 | 0.8 | 0.1 | 1.1 | 0.0 | 1.4 | 0.2 | 1.0 | 0.0 | 1.0 | 0.1 | 0.8 | 0.1 | 0.8 | 0.1 | 0.7 | 0.0 | 1.3 | 0.1 |
| **1BGCA** | **174** | **α** | **4255** | 42.6 | 1.1 | 0.1 | 1.3 | 0.1 | 1.4 | 0.1 | 0.9 | 0.1 | 0.5 | 0.1 | 1.0 | 0.0 | 1.1 | 0.3 | 1.2 | 0.0 | 2.2 | 0.4 | 1.0 | 0.0 | 1.3 | 0.0 | 1.1 | 0.1 | 1.5 | 0.3 |
| **1BJ7A** | **156** | **αβ** | **9080** | 90.8 | 1.1 | 0.3 | 0.9 | 0.3 | 2.1 | 0.7 | 0.1 | 0.1 | 1.3 | 0.3 | 1.1 | 0.1 | 2.8 | 1.3 | 0.8 | 0.1 | 3.9 | 2.1 | 4.8 | 1.2 | 4.9 | 1.2 | 4.6 | 1.2 | 3.6 | 1.5 |
| **1BZ4A** | **144** | **α** | **9136** | 91.4 | 1.3 | 0.4 | 1.6 | 0.7 | 1.6 | 0.4 | 0.9 | 0.2 | 0.4 | 0.3 | 0.9 | 0.0 | 0.4 | 0.3 | 1.2 | 0.3 | 2.2 | 1.1 | 0.9 | 0.0 | 1.9 | 0.6 | 1.7 | 0.5 | 0.8 | 0.4 |
| **1CHDA** | **203** | **αβ** | **108** | 1.1 | 1.2 | 0.1 | 1.4 | 0.1 | 2.2 | 0.3 | 0.5 | 0.2 | 1.9 | 0.3 | 1.0 | 0.0 | 2.6 | 0.3 | 0.9 | 0.0 | 2.0 | 0.4 | 1.4 | 0.1 | 1.1 | 0.2 | 1.4 | 0.2 | 2.1 | 0.3 |
| **1DUSA** | **194** | **αβ** | **2553** | 25.5 | 0.9 | 0.1 | 0.8 | 0.0 | 2.2 | 0.5 | 0.2 | 0.1 | 2.3 | 0.4 | 1.0 | 0.0 | 1.9 | 0.2 | 1.0 | 0.0 | 3.3 | 0.5 | 1.7 | 0.3 | 2.4 | 0.4 | 2.5 | 0.4 | 1.9 | 0.3 |
| **1EYHA** | **144** | **α** | **9291** | 92.9 | 0.9 | 0.3 | 0.9 | 0.3 | 1.0 | 0.5 | 0.7 | 0.2 | 1.0 | 0.3 | 1.0 | 0.1 | 1.5 | 0.7 | 1.0 | 0.2 | 2.0 | 1.1 | 1.2 | 0.4 | 1.7 | 0.8 | 1.3 | 0.2 | 1.7 | 0.7 |
| **1GAKA** | **141** | **α** | **8225** | 82.3 | 1.5 | 0.2 | 1.3 | 0.5 | 0.6 | 0.1 | 2.6 | 0.5 | 0.2 | 0.2 | 1.0 | 0.0 | 0.2 | 0.2 | 1.1 | 0.1 | 3.2 | 1.5 | 1.0 | 0.0 | 3.0 | 1.1 | 1.6 | 0.3 | 0.6 | 0.3 |
| **1GS9A** | **165** | **α** | **6203** | 62.0 | 1.2 | 0.2 | 1.2 | 0.1 | 1.3 | 0.2 | 1.2 | 0.3 | 0.5 | 0.2 | 1.0 | 0.0 | 0.7 | 0.3 | 1.2 | 0.1 | 2.2 | 0.8 | 1.0 | 0.0 | 1.3 | 0.2 | 1.4 | 0.2 | 1.2 | 0.3 |
| **1IAPA** | **211** | **α** | **612** | 6.1 | 1.1 | 0.0 | 1.3 | 0.0 | 1.3 | 0.0 | 0.7 | 0.1 | 0.8 | 0.0 | 1.0 | 0.0 | 1.6 | 0.1 | 1.0 | 0.0 | 1.4 | 0.1 | 1.0 | 0.0 | 1.0 | 0.0 | 1.0 | 0.0 | 2.0 | 0.1 |
| **1ICXA** | **155** | **αβ** | **6638** | 66.4 | 0.9 | 0.1 | 0.9 | 0.2 | 1.7 | 0.3 | 0.1 | 0.1 | 1.6 | 0.1 | 0.8 | 0.1 | 2.1 | 0.8 | 1.0 | 0.0 | 2.7 | 1.0 | 3.2 | 1.0 | 3.2 | 0.8 | 3.0 | 0.7 | 2.5 | 0.9 |
| **1JL1A** | **155** | **αβ** | **1802** | 18.0 | 1.1 | 0.0 | 1.2 | 0.1 | 1.7 | 0.1 | 0.5 | 0.1 | 1.3 | 0.1 | 1.0 | 0.0 | 1.5 | 0.1 | 1.0 | 0.0 | 2.0 | 0.2 | 1.7 | 0.1 | 1.5 | 0.1 | 1.4 | 0.2 | 1.4 | 0.1 |
| **1K6KA** | **143** | **α** | **9867** | 98.7 | 1.5 | 0.7 | 2.4 | 0.6 | 1.8 | 0.9 | 0.3 | 0.5 | 1.3 | 0.5 | 0.8 | 0.0 | 2.1 | 0.7 | 0.8 | 0.0 | 2.0 | 1.0 | 0.8 | 0.0 | 2.6 | 1.3 | 1.9 | 1.1 | 2.5 | 1.2 |
| **1LKIA** | **180** | **α** | **1950** | 19.5 | 1.1 | 0.1 | 1.1 | 0.0 | 0.9 | 0.1 | 1.0 | 0.2 | 0.4 | 0.1 | 1.0 | 0.0 | 0.9 | 0.1 | 1.1 | 0.0 | 1.6 | 0.4 | 0.9 | 0.0 | 1.4 | 0.1 | 0.9 | 0.1 | 1.2 | 0.1 |
| **1LWBA** | **122** | **α** | **8647** | 86.5 | 1.2 | 0.3 | 1.4 | 0.3 | 1.4 | 0.3 | 1.0 | 0.3 | 0.6 | 0.2 | 1.0 | 0.0 | 0.9 | 0.2 | 1.0 | 0.0 | 2.4 | 0.7 | 1.0 | 0.0 | 1.7 | 0.3 | 0.8 | 0.2 | 1.6 | 0.5 |
| **1M5IA** | **125** | **α** | **9819** | 98.2 | 1.3 | 0.6 | 1.7 | 0.8 | 1.0 | 0.5 | 1.0 | 0.5 | 0.1 | 0.2 | 1.0 | 0.0 | 0.1 | 0.2 | 1.3 | 0.3 | 2.3 | 1.7 | 1.0 | 0.0 | 3.4 | 1.4 | 2.7 | 0.6 | 0.4 | 0.5 |
| **1NFNA** | **191** | **α** | **2443** | 24.4 | 1.0 | 0.0 | 1.2 | 0.1 | 1.2 | 0.1 | 1.1 | 0.1 | 0.3 | 0.1 | 1.0 | 0.0 | 0.4 | 0.1 | 1.3 | 0.1 | 1.9 | 0.1 | 1.0 | 0.0 | 1.7 | 0.3 | 1.2 | 0.2 | 0.7 | 0.0 |
| **1OZ9A** | **150** | **αβ** | **8588** | 85.9 | 0.9 | 0.1 | 1.2 | 0.2 | 1.7 | 0.4 | 0.5 | 0.1 | 0.6 | 0.2 | 1.0 | 0.0 | 1.6 | 0.4 | 1.0 | 0.1 | 1.3 | 0.5 | 0.9 | 0.2 | 1.5 | 0.3 | 1.3 | 0.2 | 1.4 | 0.3 |
| **1ROAA** | **122** | **β** | **7862** | 78.6 | 0.9 | 0.1 | 1.2 | 0.2 | 1.4 | 0.1 | 0.7 | 0.2 | 1.0 | 0.3 | 1.0 | 0.0 | 1.1 | 0.2 | 1.0 | 0.0 | 1.5 | 0.3 | 1.4 | 0.2 | 1.6 | 0.3 | 1.5 | 0.4 | 1.4 | 0.3 |
| **1TZVA** | **142** | **α** | **9771** | 97.7 | 0.9 | 0.3 | 1.6 | 0.9 | 2.6 | 1.7 | 0.4 | 0.4 | 1.4 | 0.7 | 0.8 | 0.0 | 3.2 | 2.1 | 0.5 | 0.2 | 3.0 | 1.8 | 0.8 | 0.0 | 1.6 | 1.0 | 1.2 | 0.6 | 4.0 | 2.0 |
| **1WBAA** | **175** | **β** | **628** | 6.3 | 0.7 | 0.1 | 1.0 | 0.0 | 1.1 | 0.1 | 0.6 | 0.1 | 1.9 | 0.3 | 0.7 | 0.0 | 1.5 | 0.1 | 1.0 | 0.0 | 2.2 | 0.2 | 2.5 | 0.3 | 2.8 | 0.6 | 3.1 | 0.5 | 2.2 | 0.1 |
| **1WVHA** | **134** | **β** | **2349** | 23.5 | 1.0 | 0.1 | 0.9 | 0.1 | 1.2 | 0.0 | 0.5 | 0.1 | 1.5 | 0.1 | 1.0 | 0.0 | 1.5 | 0.1 | 1.0 | 0.0 | 1.4 | 0.1 | 2.0 | 0.1 | 2.3 | 0.2 | 2.2 | 0.2 | 1.6 | 0.1 |
| **1XGWA** | **176** | **α** | **1797** | 18.0 | 0.9 | 0.1 | 1.0 | 0.1 | 0.8 | 0.0 | 0.9 | 0.0 | 0.9 | 0.0 | 1.0 | 0.0 | 1.4 | 0.0 | 1.0 | 0.0 | 1.4 | 0.1 | 1.0 | 0.0 | 1.0 | 0.0 | 1.0 | 0.0 | 1.4 | 0.1 |
| **1XKRA** | **206** | **αβ** | **268** | 2.7 | 1.1 | 0.1 | 0.6 | 0.1 | 1.7 | 0.1 | 0.8 | 0.0 | 0.8 | 0.2 | 0.9 | 0.0 | 1.9 | 0.1 | 1.1 | 0.1 | 1.3 | 0.1 | 1.2 | 0.0 | 1.4 | 0.1 | 1.2 | 0.1 | 1.8 | 0.1 |
| **2EJXA** | **139** | **αβ** | **7536** | 75.4 | 0.9 | 0.1 | 1.2 | 0.2 | 1.1 | 0.1 | 0.2 | 0.1 | 1.3 | 0.1 | 1.0 | 0.0 | 1.7 | 0.6 | 1.0 | 0.0 | 2.1 | 0.6 | 3.6 | 1.0 | 4.3 | 1.2 | 3.8 | 0.9 | 2.3 | 0.7 |
| **2FM9A** | **215** | **α** | **1464** | 14.6 | 0.9 | 0.0 | 1.3 | 0.0 | 1.2 | 0.0 | 0.5 | 0.1 | 0.7 | 0.0 | 1.0 | 0.0 | 1.8 | 0.1 | 1.1 | 0.0 | 2.4 | 0.2 | 1.0 | 0.0 | 1.6 | 0.1 | 1.2 | 0.0 | 1.9 | 0.1 |
| **2IU1A** | **208** | **α** | **911** | 9.1 | 0.7 | 0.0 | 1.2 | 0.0 | 1.5 | 0.0 | 0.3 | 0.0 | 1.2 | 0.0 | 1.0 | 0.0 | 1.9 | 0.0 | 1.1 | 0.0 | 2.4 | 0.1 | 1.1 | 0.0 | 0.9 | 0.0 | 0.8 | 0.0 | 1.6 | 0.0 |
| **2OF3A** | **266** | **α** | **305** | 3.1 | 0.9 | 0.1 | 1.6 | 0.1 | 1.4 | 0.2 | 0.2 | 0.1 | 1.3 | 0.1 | 0.9 | 0.0 | 1.6 | 0.2 | 1.0 | 0.0 | 2.3 | 0.3 | 1.0 | 0.0 | 1.1 | 0.0 | 1.5 | 0.2 | 1.0 | 0.1 |
| **2OSAA** | **202** | **α** | **2674** | 26.7 | 1.0 | 0.1 | 1.0 | 0.1 | 1.2 | 0.1 | 0.8 | 0.0 | 0.9 | 0.1 | 1.0 | 0.0 | 1.4 | 0.1 | 1.0 | 0.0 | 1.7 | 0.1 | 1.0 | 0.0 | 1.4 | 0.0 | 1.0 | 0.0 | 1.5 | 0.1 |
| **2YV8A** | **164** | **β** | **1539** | 15.4 | 1.2 | 0.0 | 1.4 | 0.0 | 1.8 | 0.0 | 0.3 | 0.0 | 1.3 | 0.1 | 1.0 | 0.0 | 1.6 | 0.1 | 1.0 | 0.0 | 2.0 | 0.1 | 1.3 | 0.1 | 1.8 | 0.1 | 1.4 | 0.0 | 1.7 | 0.0 |
| **GDT_TS > 25% bcl_perturb** | | | | |  |  |  |  |  |  |  |  |  |  |  |  |  |  |  |  |  |  |  |  |  |  |  |  |  |  |
| **1AAJA** | **105** | **β** | **3423** | 28.5 | 1.9 | 0.3 | 2.1 | 0.4 | 3.2 | 0.6 | 1.1 | 0.1 | 3.6 | 1.0 | 2.1 | 0.6 | 1.7 | 0.1 | 2.2 | 0.3 | 3.3 | 0.5 | 4.1 | 0.6 | 0.4 | 0.0 | 0.4 | 0.1 | 4.2 | 0.7 |
| **1BGCA** | **174** | **α** | **2998** | 25.0 | 0.7 | 0.0 | 0.8 | 0.1 | 1.5 | 0.1 | 1.0 | 0.1 | 0.9 | 0.2 | 1.2 | 0.0 | 1.1 | 0.1 | 1.0 | 0.0 | 1.9 | 0.2 | 1.0 | 0.0 | 1.4 | 0.0 | 1.4 | 0.0 | 1.8 | 0.1 |
| **1BJ7A** | **156** | **αβ** | **2240** | 18.7 | 2.3 | 0.3 | 2.3 | 0.3 | 3.6 | 0.4 | 0.1 | 0.0 | 5.2 | 0.9 | 3.3 | 0.6 | 0.8 | 0.1 | 2.1 | 0.1 | 3.8 | 0.2 | 4.3 | 0.5 | 1.3 | 0.0 | 1.3 | 0.0 | 2.6 | 0.1 |
| **1BZ4A** | **144** | **α** | **11199** | 93.3 | 0.2 | 0.1 | 0.6 | 0.4 | 1.5 | 1.4 | 3.6 | 0.5 | 0.6 | 0.9 | 0.2 | 0.3 | 0.0 | 0.0 | 0.8 | 0.1 | 3.8 | 1.2 | 0.9 | 0.0 | 9.7 | 0.2 | 9.7 | 0.2 | 4.0 | 1.5 |
| **1CHDA** | **203** | **αβ** | **2697** | 22.5 | 3.3 | 0.5 | 3.7 | 0.6 | 2.0 | 0.1 | 0.0 | 0.0 | 5.6 | 1.0 | 3.6 | 0.7 | 0.5 | 0.1 | 2.2 | 0.2 | 4.4 | 0.8 | 3.8 | 0.3 | 1.3 | 0.0 | 1.3 | 0.0 | 1.7 | 0.1 |
| **1DUSA** | **194** | **αβ** | **5168** | 43.1 | 3.1 | 0.9 | 3.3 | 1.1 | 4.1 | 1.4 | 0.5 | 0.2 | 5.5 | 1.7 | 3.4 | 1.5 | 1.4 | 0.2 | 1.8 | 0.1 | 4.1 | 1.3 | 4.7 | 1.0 | 1.3 | 0.0 | 1.3 | 0.0 | 4.4 | 1.1 |
| **1EYHA** | **144** | **α** | **3492** | 29.1 | 1.3 | 0.2 | 1.5 | 0.2 | 3.0 | 0.6 | 0.9 | 0.2 | 4.1 | 1.3 | 1.7 | 0.7 | 1.3 | 0.1 | 1.1 | 0.0 | 2.0 | 0.2 | 1.0 | 0.0 | 1.3 | 0.0 | 1.3 | 0.0 | 2.7 | 0.3 |
| **1G8AA** | **227** | **αβ** | **5107** | 42.6 | 3.6 | 1.1 | 3.9 | 1.2 | 3.0 | 0.9 | 0.3 | 0.1 | 6.0 | 1.7 | 4.1 | 1.7 | 0.5 | 0.2 | 2.3 | 0.4 | 5.1 | 1.7 | 4.7 | 0.9 | 1.3 | 0.1 | 1.1 | 0.0 | 3.4 | 0.6 |
| **1GAKA** | **141** | **α** | **6684** | 55.7 | 0.6 | 0.3 | 0.7 | 0.4 | 2.4 | 1.2 | 1.2 | 0.2 | 1.7 | 0.9 | 0.9 | 0.5 | 1.0 | 0.2 | 0.8 | 0.0 | 2.2 | 0.7 | 1.0 | 0.0 | 1.3 | 0.1 | 1.1 | 0.1 | 2.9 | 1.3 |
| **1GCUA** | **295** | **αβ** | **5247** | 43.7 | 3.2 | 0.9 | 3.6 | 1.1 | 4.2 | 1.4 | 0.7 | 0.2 | 4.9 | 1.6 | 4.0 | 1.7 | 0.4 | 0.2 | 2.1 | 0.3 | 3.8 | 1.2 | 4.2 | 0.9 | 1.2 | 0.0 | 1.0 | 0.0 | 3.4 | 0.8 |
| **1GS9A** | **165** | **α** | **9233** | 76.9 | 0.2 | 0.1 | 0.3 | 0.2 | 2.0 | 1.3 | 1.2 | 0.1 | 2.4 | 1.6 | 0.8 | 0.6 | 0.1 | 0.1 | 0.7 | 0.0 | 2.8 | 1.2 | 1.0 | 0.0 | 1.8 | 0.1 | 1.8 | 0.1 | 3.1 | 1.3 |
| **1HYPA** | **80** | **α** | **5480** | 45.7 | 0.5 | 0.1 | 0.9 | 0.4 | 1.8 | 0.3 | 1.1 | 0.2 | 2.4 | 0.8 | 1.2 | 0.2 | 1.4 | 0.1 | 1.0 | 0.0 | 1.6 | 0.3 | 1.0 | 0.0 | 0.6 | 0.1 | 0.6 | 0.1 | 2.0 | 0.5 |
| **1IAPA** | **211** | **α** | **3387** | 28.2 | 1.8 | 0.2 | 2.4 | 0.4 | 2.7 | 0.6 | 1.3 | 0.1 | 4.7 | 1.2 | 2.7 | 1.0 | 1.3 | 0.1 | 0.9 | 0.0 | 2.9 | 0.4 | 1.0 | 0.0 | 1.1 | 0.0 | 1.1 | 0.0 | 3.7 | 0.5 |
| **1ICXA** | **155** | **αβ** | **4439** | 37.0 | 2.5 | 0.8 | 2.5 | 0.8 | 2.4 | 0.8 | 1.4 | 0.1 | 4.9 | 1.5 | 2.8 | 1.2 | 1.7 | 0.2 | 2.5 | 0.4 | 4.0 | 1.2 | 4.4 | 0.9 | 1.3 | 0.0 | 1.1 | 0.0 | 5.0 | 1.4 |
| **1IFBA** | **131** | **β** | **7656** | 63.8 | 2.8 | 1.3 | 2.8 | 1.2 | 2.5 | 0.9 | 0.7 | 0.2 | 6.5 | 2.4 | 3.8 | 2.3 | 1.6 | 0.3 | 1.9 | 0.4 | 3.6 | 1.5 | 5.0 | 2.0 | 1.3 | 0.1 | 1.3 | 0.1 | 5.0 | 2.0 |
| **1J27A** | **102** | **β** | **8427** | 70.2 | 0.8 | 0.4 | 0.8 | 0.5 | 1.9 | 0.9 | 0.9 | 0.3 | 2.9 | 1.5 | 1.1 | 0.6 | 0.5 | 0.2 | 0.8 | 0.2 | 2.8 | 1.7 | 2.0 | 0.7 | 1.5 | 0.1 | 1.5 | 0.1 | 2.6 | 1.5 |
| **1JL1A** | **155** | **αβ** | **3593** | 29.9 | 1.8 | 0.5 | 1.9 | 0.5 | 3.4 | 0.7 | 1.3 | 0.1 | 4.0 | 1.2 | 1.7 | 0.6 | 0.2 | 0.1 | 1.4 | 0.0 | 3.5 | 0.9 | 3.5 | 0.6 | 1.0 | 0.0 | 1.0 | 0.0 | 3.5 | 0.9 |
| **1K6KA** | **143** | **α** | **3933** | 32.8 | 1.7 | 0.4 | 2.1 | 0.6 | 1.8 | 0.3 | 1.3 | 0.1 | 3.4 | 1.2 | 2.0 | 0.8 | 1.3 | 0.1 | 1.4 | 0.1 | 2.8 | 0.5 | 1.0 | 0.0 | 1.3 | 0.0 | 1.3 | 0.0 | 3.4 | 0.6 |
| **1LKFA** | **299** | **αβ** | **3716** | 31.0 | 3.4 | 0.6 | 3.7 | 0.7 | 4.1 | 0.9 | 0.6 | 0.2 | 4.7 | 1.2 | 4.1 | 1.3 | 0.4 | 0.2 | 2.7 | 0.5 | 5.1 | 0.9 | 6.2 | 0.9 | 1.0 | 0.0 | 0.8 | 0.0 | 5.0 | 0.7 |
| **1LKIA** | **180** | **α** | **2753** | 22.9 | 0.4 | 0.0 | 0.5 | 0.1 | 1.3 | 0.1 | 1.1 | 0.0 | 1.8 | 0.1 | 1.4 | 0.1 | 1.0 | 0.1 | 1.0 | 0.0 | 1.8 | 0.3 | 1.0 | 0.0 | 1.4 | 0.0 | 1.4 | 0.0 | 1.7 | 0.3 |
| **1LWBA** | **122** | **α** | **3384** | 28.2 | 0.7 | 0.1 | 0.7 | 0.2 | 1.4 | 0.1 | 1.1 | 0.1 | 2.4 | 0.8 | 0.8 | 0.3 | 0.9 | 0.2 | 1.0 | 0.1 | 1.8 | 0.5 | 1.0 | 0.0 | 1.1 | 0.0 | 0.7 | 0.1 | 2.0 | 0.4 |
| **1M5IA** | **125** | **α** | **11730** | 97.8 | 0.2 | 0.2 | 2.1 | 1.1 | 6.2 | 0.9 | 8.8 | 0.8 | 1.9 | 2.7 | 0.1 | 0.2 | 0.0 | 0.0 | 0.7 | 0.0 | 6.5 | 1.1 | 1.0 | 0.0 | 9.6 | 0.1 | 9.6 | 0.1 | 8.3 | 1.2 |
| **1NFNA** | **191** | **α** | **2657** | 22.1 | 0.4 | 0.0 | 0.5 | 0.1 | 1.7 | 0.4 | 1.0 | 0.1 | 3.3 | 0.6 | 1.6 | 0.2 | 1.6 | 0.1 | 1.0 | 0.0 | 1.9 | 0.1 | 1.0 | 0.0 | 1.3 | 0.0 | 1.3 | 0.0 | 1.8 | 0.1 |
| **1OZ9A** | **150** | **αβ** | **4516** | 37.6 | 2.2 | 0.7 | 2.6 | 0.9 | 4.2 | 1.2 | 0.8 | 0.1 | 4.2 | 1.5 | 2.0 | 0.8 | 1.1 | 0.1 | 1.0 | 0.1 | 3.3 | 1.0 | 3.3 | 0.7 | 1.3 | 0.1 | 1.0 | 0.1 | 3.9 | 1.2 |
| **1PRZA** | **252** | **αβ** | **3389** | 28.2 | 3.1 | 0.8 | 3.4 | 0.9 | 1.7 | 0.2 | 0.3 | 0.2 | 5.4 | 1.3 | 3.3 | 1.0 | 0.8 | 0.1 | 2.5 | 0.3 | 3.3 | 0.8 | 4.1 | 0.5 | 1.1 | 0.0 | 0.9 | 0.0 | 3.7 | 0.5 |
| **1ROAA** | **122** | **β** | **1264** | 10.5 | 1.0 | 0.0 | 1.2 | 0.0 | 2.0 | 0.0 | 0.8 | 0.0 | 3.8 | 0.1 | 2.1 | 0.1 | 2.3 | 0.0 | 1.3 | 0.0 | 2.9 | 0.1 | 2.1 | 0.1 | 1.2 | 0.0 | 1.0 | 0.0 | 3.2 | 0.1 |
| **1TZVA** | **142** | **α** | **3734** | 31.1 | 1.5 | 0.3 | 1.9 | 0.4 | 3.6 | 1.1 | 0.8 | 0.2 | 3.7 | 1.4 | 1.5 | 0.7 | 1.4 | 0.1 | 0.9 | 0.0 | 2.4 | 0.4 | 1.0 | 0.0 | 1.3 | 0.0 | 1.3 | 0.0 | 2.9 | 0.5 |
| **1UBIA** | **76** | **β** | **3952** | 32.9 | 0.6 | 0.1 | 0.7 | 0.2 | 2.2 | 0.8 | 1.1 | 0.1 | 2.3 | 0.4 | 1.3 | 0.1 | 1.3 | 0.2 | 1.0 | 0.2 | 1.9 | 0.7 | 1.9 | 0.4 | 1.3 | 0.1 | 1.3 | 0.1 | 2.2 | 0.9 |
| **1UEKA** | **275** | **αβ** | **5380** | 44.8 | 2.8 | 1.2 | 3.3 | 1.4 | 3.5 | 1.1 | 0.6 | 0.2 | 5.0 | 1.7 | 3.7 | 1.6 | 1.6 | 0.3 | 2.8 | 0.9 | 4.1 | 1.5 | 4.5 | 1.3 | 1.3 | 0.1 | 1.0 | 0.0 | 4.3 | 1.4 |
| **1VGJA** | **184** | **αβ** | **6496** | 54.1 | 3.2 | 1.4 | 3.7 | 1.7 | 4.1 | 1.7 | 1.1 | 0.1 | 4.7 | 2.0 | 3.3 | 1.7 | 0.4 | 0.1 | 3.0 | 1.1 | 4.3 | 1.6 | 4.4 | 1.4 | 1.2 | 0.0 | 1.0 | 0.0 | 5.5 | 2.1 |
| **1VK4A** | **298** | **αβ** | **5223** | 43.5 | 3.9 | 1.2 | 4.1 | 1.3 | 1.2 | 0.1 | 0.3 | 0.2 | 6.0 | 1.7 | 4.8 | 1.8 | 0.1 | 0.1 | 1.0 | 0.1 | 4.6 | 1.4 | 4.8 | 0.8 | 1.3 | 0.0 | 1.3 | 0.0 | 1.3 | 0.3 |
| **1WBAA** | **175** | **β** | **501** | 4.2 | 3.0 | 0.5 | 3.3 | 0.6 | 3.6 | 0.9 | 0.1 | 0.0 | 6.0 | 0.7 | 2.3 | 0.2 | 0.3 | 0.1 | 2.1 | 0.3 | 3.5 | 0.9 | 4.8 | 1.3 | 1.3 | 0.1 | 1.3 | 0.1 | 0.3 | 0.1 |
| **1WNHA** | **225** | **αβ** | **4644** | 38.7 | 3.3 | 1.1 | 3.6 | 1.3 | 3.9 | 0.9 | 0.6 | 0.1 | 4.6 | 1.4 | 3.3 | 1.2 | 1.7 | 0.2 | 3.1 | 0.7 | 4.3 | 1.5 | 4.7 | 1.0 | 1.1 | 0.0 | 0.7 | 0.1 | 4.7 | 1.2 |
| **1WR2A** | **238** | **αβ** | **4542** | 37.9 | 3.2 | 0.9 | 3.7 | 1.2 | 4.5 | 1.3 | 0.8 | 0.2 | 4.8 | 1.5 | 3.5 | 1.4 | 0.6 | 0.2 | 2.2 | 0.4 | 4.0 | 1.1 | 4.1 | 0.7 | 1.3 | 0.0 | 1.3 | 0.0 | 4.7 | 1.2 |
| **1WVHA** | **134** | **β** | **3472** | 28.9 | 2.3 | 0.7 | 2.5 | 0.7 | 1.5 | 0.1 | 1.8 | 0.3 | 3.4 | 0.9 | 1.8 | 0.5 | 0.4 | 0.0 | 2.4 | 0.4 | 3.4 | 0.9 | 4.0 | 0.7 | 1.0 | 0.0 | 0.8 | 0.0 | 3.9 | 1.0 |
| **1X91A** | **153** | **α** | **8405** | 70.0 | 0.3 | 0.1 | 0.4 | 0.1 | 2.5 | 1.1 | 1.1 | 0.1 | 2.1 | 1.2 | 0.6 | 0.3 | 1.1 | 0.2 | 1.0 | 0.0 | 2.6 | 0.8 | 1.0 | 0.0 | 1.5 | 0.1 | 1.4 | 0.1 | 2.6 | 0.9 |
| **1XGWA** | **176** | **α** | **1435** | 12.0 | 1.5 | 0.0 | 2.0 | 0.1 | 4.2 | 0.2 | 0.3 | 0.0 | 5.0 | 0.3 | 2.6 | 0.2 | 0.2 | 0.0 | 1.1 | 0.0 | 1.8 | 0.0 | 1.0 | 0.0 | 1.2 | 0.0 | 1.2 | 0.0 | 0.7 | 0.0 |
| **1XKRA** | **206** | **αβ** | **4875** | 40.6 | 2.4 | 0.8 | 3.0 | 1.1 | 1.8 | 0.5 | 1.2 | 0.1 | 3.9 | 1.2 | 2.3 | 1.0 | 1.0 | 0.2 | 2.4 | 0.5 | 3.6 | 1.2 | 3.7 | 0.8 | 1.3 | 0.1 | 1.1 | 0.1 | 4.7 | 1.6 |
| **1XQOA** | **256** | **α** | **4936** | 41.1 | 2.5 | 0.4 | 3.3 | 0.8 | 3.7 | 1.0 | 0.8 | 0.2 | 5.4 | 1.7 | 3.9 | 1.6 | 0.7 | 0.2 | 1.2 | 0.1 | 2.8 | 0.4 | 1.0 | 0.0 | 1.1 | 0.0 | 1.0 | 0.0 | 3.8 | 0.7 |
| **2CWYA** | **94** | **α** | **11371** | 94.8 | 0.1 | 0.1 | 0.4 | 0.2 | 3.4 | 2.2 | 2.5 | 0.7 | 1.0 | 1.9 | 0.1 | 0.1 | 0.1 | 0.1 | 0.9 | 0.1 | 4.6 | 1.2 | 1.0 | 0.0 | 9.6 | 0.4 | 9.6 | 0.4 | 5.8 | 1.3 |
| **2E3SA** | **255** | **αβ** | **3502** | 29.2 | 3.4 | 0.8 | 3.7 | 0.8 | 4.1 | 1.1 | 0.4 | 0.2 | 4.5 | 1.1 | 3.0 | 0.9 | 0.2 | 0.1 | 2.7 | 0.3 | 4.1 | 0.9 | 4.6 | 0.8 | 1.3 | 0.0 | 1.3 | 0.0 | 3.1 | 0.6 |
| **2EJXA** | **139** | **αβ** | **11561** | 96.3 | 0.5 | 0.9 | 0.6 | 1.1 | 3.7 | 1.9 | 3.3 | 1.1 | 4.9 | 2.4 | 1.2 | 2.4 | 0.0 | 0.0 | 0.5 | 0.6 | 2.0 | 2.2 | 3.1 | 1.9 | 7.8 | 0.4 | 7.8 | 0.2 | 4.1 | 2.9 |
| **2FM9A** | **215** | **α** | **4403** | 36.7 | 1.9 | 0.4 | 2.4 | 0.7 | 1.9 | 0.4 | 0.9 | 0.2 | 4.9 | 1.5 | 2.0 | 0.7 | 1.3 | 0.1 | 0.9 | 0.1 | 3.3 | 0.6 | 1.0 | 0.0 | 1.3 | 0.1 | 1.3 | 0.1 | 3.8 | 0.8 |
| **2ILRA** | **264** | **α** | **6048** | 50.4 | 2.3 | 0.5 | 3.3 | 1.0 | 3.4 | 1.2 | 0.2 | 0.2 | 5.7 | 2.0 | 3.6 | 1.8 | 0.5 | 0.1 | 1.1 | 0.1 | 3.1 | 0.5 | 1.0 | 0.0 | 1.3 | 0.1 | 1.3 | 0.1 | 1.7 | 0.5 |
| **2IU1A** | **208** | **α** | **2838** | 23.7 | 1.7 | 0.2 | 2.2 | 0.4 | 3.4 | 0.6 | 0.5 | 0.2 | 4.2 | 1.0 | 2.9 | 0.8 | 0.6 | 0.1 | 1.1 | 0.0 | 3.2 | 0.4 | 1.0 | 0.0 | 1.0 | 0.0 | 1.0 | 0.0 | 2.5 | 0.2 |
| **2OF3A** | **266** | **α** | **5231** | 43.6 | 2.3 | 0.5 | 3.0 | 0.7 | 4.2 | 1.2 | 0.4 | 0.2 | 5.6 | 1.8 | 3.4 | 1.6 | 0.6 | 0.1 | 1.2 | 0.0 | 3.2 | 0.5 | 1.0 | 0.0 | 1.3 | 0.1 | 1.3 | 0.1 | 2.0 | 0.5 |
| **2OPWA** | **291** | **αβ** | **1178** | 9.8 | 3.6 | 0.0 | 4.0 | 0.0 | 3.9 | 0.1 | 0.7 | 0.0 | 4.3 | 0.0 | 4.4 | 0.0 | 2.2 | 0.0 | 2.4 | 0.0 | 3.6 | 0.0 | 3.7 | 0.0 | 0.5 | 0.0 | 0.5 | 0.0 | 4.6 | 0.1 |
| **2OSAA** | **202** | **α** | **3524** | 29.4 | 1.7 | 0.3 | 2.1 | 0.4 | 4.4 | 0.9 | 0.7 | 0.2 | 4.1 | 1.2 | 2.4 | 0.8 | 1.2 | 0.0 | 1.2 | 0.0 | 2.0 | 0.2 | 1.0 | 0.0 | 1.1 | 0.0 | 0.7 | 0.1 | 2.8 | 0.2 |
| **2YV8A** | **164** | **β** | **1590** | 13.3 | 2.2 | 0.1 | 2.7 | 0.1 | 1.7 | 0.0 | 1.2 | 0.1 | 4.2 | 0.3 | 3.1 | 0.2 | 0.2 | 0.0 | 1.8 | 0.1 | 3.6 | 0.2 | 3.6 | 0.2 | 1.1 | 0.0 | 1.0 | 0.0 | 1.1 | 0.0 |
| **2YVTA** | **260** | **αβ** | **4312** | 35.9 | 3.7 | 1.0 | 4.1 | 1.1 | 3.8 | 1.1 | 0.8 | 0.2 | 5.0 | 1.2 | 4.2 | 1.4 | 1.8 | 0.3 | 2.5 | 0.4 | 4.4 | 1.3 | 4.2 | 0.6 | 1.3 | 0.0 | 1.1 | 0.0 | 4.3 | 0.9 |
| **2ZCOA** | **293** | **α** | **4957** | 41.3 | 2.6 | 0.6 | 3.1 | 0.9 | 4.2 | 1.3 | 0.4 | 0.2 | 5.3 | 1.7 | 3.4 | 1.5 | 0.9 | 0.1 | 1.0 | 0.1 | 4.2 | 1.0 | 1.0 | 0.0 | 1.1 | 0.0 | 1.1 | 0.0 | 2.6 | 0.4 |
| **3B5OA** | **244** | **α** | **4002** | 33.4 | 1.7 | 0.4 | 2.0 | 0.6 | 3.1 | 0.9 | 1.3 | 0.1 | 3.4 | 1.1 | 2.0 | 0.8 | 1.4 | 0.1 | 1.2 | 0.1 | 3.7 | 0.8 | 1.0 | 0.0 | 1.3 | 0.0 | 1.1 | 0.0 | 3.9 | 0.9 |
| **GDT_TS > 25% bcl_fold** | | | | |  |  |  |  |  |  |  |  |  |  |  |  |  |  |  |  |  |  |  |  |  |  |  |  |  |  |
| **1BGCA** | **174** | **α** | **4035** | 40.4 | 1.2 | 0.1 | 1.8 | 0.2 | 1.0 | 0.1 | 0.5 | 0.1 | 0.5 | 0.3 | 1.3 | 0.1 | 0.7 | 0.1 | 1.0 | 0.0 | 1.9 | 0.6 | 1.0 | 0.0 | 1.0 | 0.0 | 1.0 | 0.0 | 1.5 | 0.2 |
| **1EYHA** | **144** | **α** | **2893** | 28.9 | 1.0 | 0.1 | 1.4 | 0.1 | 1.3 | 0.1 | 1.0 | 0.1 | 1.4 | 0.3 | 0.9 | 0.1 | 1.0 | 0.1 | 1.0 | 0.0 | 1.6 | 0.4 | 1.0 | 0.0 | 1.0 | 0.0 | 1.0 | 0.0 | 1.6 | 0.3 |
| **1GAKA** | **141** | **α** | **9106** | 91.1 | 1.1 | 0.2 | 1.2 | 0.2 | 1.0 | 0.4 | 0.8 | 0.3 | 1.8 | 0.5 | 1.4 | 0.5 | 1.7 | 0.4 | 1.0 | 0.0 | 1.1 | 0.5 | 0.9 | 0.0 | 1.0 | 0.0 | 0.8 | 0.0 | 1.8 | 0.3 |
| **1GS9A** | **165** | **α** | **9238** | 92.4 | 1.1 | 0.3 | 2.1 | 1.8 | 0.4 | 0.6 | 4.5 | 2.8 | 5.7 | 2.9 | 2.6 | 1.3 | 1.0 | 0.0 | 1.0 | 0.0 | 2.1 | 1.7 | 1.0 | 0.0 | 1.0 | 0.0 | 1.0 | 0.0 | 5.1 | 2.2 |
| **1HYPA** | **80** | **α** | **7459** | 74.6 | 1.1 | 0.2 | 1.1 | 0.3 | 0.7 | 0.2 | 0.8 | 0.1 | 2.4 | 0.8 | 1.3 | 0.2 | 1.0 | 0.1 | 1.0 | 0.0 | 0.9 | 0.1 | 1.0 | 0.0 | 1.0 | 0.0 | 1.0 | 0.0 | 1.0 | 0.2 |
| **1ICXA** | **155** | **αβ** | **1403** | 14.0 | 1.3 | 0.0 | 0.7 | 0.0 | 0.7 | 0.0 | 0.5 | 0.0 | 1.7 | 0.2 | 1.3 | 0.1 | 1.2 | 0.0 | 1.0 | 0.0 | 2.0 | 0.0 | 2.0 | 0.0 | 1.0 | 0.0 | 0.9 | 0.0 | 1.9 | 0.0 |
| **1K6KA** | **143** | **α** | **483** | 4.8 | 1.1 | 0.0 | 1.5 | 0.1 | 1.2 | 0.1 | 1.0 | 0.0 | 2.0 | 0.4 | 1.6 | 0.1 | 0.9 | 0.1 | 1.0 | 0.0 | 1.0 | 0.1 | 1.0 | 0.0 | 1.0 | 0.0 | 1.0 | 0.0 | 1.2 | 0.0 |
| **1LKIA** | **180** | **α** | **5393** | 53.9 | 1.4 | 0.2 | 1.6 | 0.3 | 0.9 | 0.2 | 1.0 | 0.2 | 1.0 | 0.3 | 1.1 | 0.1 | 0.9 | 0.1 | 1.0 | 0.0 | 3.1 | 0.9 | 1.0 | 0.0 | 0.9 | 0.0 | 0.9 | 0.0 | 2.7 | 0.9 |
| **1LWBA** | **122** | **α** | **6327** | 63.3 | 0.9 | 0.1 | 1.6 | 0.4 | 0.7 | 0.3 | 2.0 | 0.3 | 1.1 | 0.4 | 1.3 | 0.3 | 0.0 | 0.0 | 1.0 | 0.0 | 1.5 | 0.6 | 1.0 | 0.0 | 1.4 | 0.0 | 0.0 | 0.0 | 2.5 | 0.6 |
| **1NFNA** | **191** | **α** | **7148** | 71.5 | 1.1 | 0.1 | 1.8 | 0.7 | 1.6 | 0.5 | 0.8 | 0.4 | 1.7 | 0.4 | 0.9 | 0.2 | 1.1 | 0.2 | 1.0 | 0.0 | 2.1 | 0.4 | 1.0 | 0.0 | 1.0 | 0.0 | 1.0 | 0.0 | 2.4 | 0.5 |
| **1OZ9A** | **150** | **αβ** | **518** | 5.2 | 1.3 | 0.0 | 1.2 | 0.0 | 1.9 | 0.1 | 0.7 | 0.1 | 2.2 | 0.3 | 1.0 | 0.1 | 1.0 | 0.0 | 1.0 | 0.0 | 1.6 | 0.1 | 0.9 | 0.1 | 1.0 | 0.0 | 1.1 | 0.0 | 1.6 | 0.2 |
| **1ROAA** | **122** | **β** | **2526** | 25.3 | 1.1 | 0.1 | 1.5 | 0.0 | 1.7 | 0.3 | 1.6 | 0.3 | 2.3 | 0.1 | 1.8 | 0.2 | 2.0 | 0.5 | 1.0 | 0.0 | 1.0 | 0.2 | 1.3 | 0.1 | 1.0 | 0.0 | 1.0 | 0.0 | 1.8 | 0.7 |
| **1TZVA** | **142** | **α** | **707** | 7.1 | 1.1 | 0.0 | 1.2 | 0.0 | 1.6 | 0.3 | 0.9 | 0.1 | 2.3 | 0.3 | 1.4 | 0.0 | 0.6 | 0.1 | 1.0 | 0.0 | 1.0 | 0.1 | 1.0 | 0.0 | 1.1 | 0.1 | 1.1 | 0.1 | 0.7 | 0.1 |
| **1UBIA** | **76** | **β** | **6234** | 62.3 | 1.1 | 0.2 | 0.8 | 0.4 | 0.6 | 0.5 | 3.6 | 1.7 | 3.9 | 2.1 | 1.1 | 0.3 | 0.8 | 0.3 | 1.0 | 0.0 | 0.1 | 0.1 | 0.6 | 0.5 | 1.0 | 0.0 | 1.0 | 0.0 | 3.6 | 1.8 |
| **1X91A** | **153** | **α** | **9650** | 96.5 | 0.7 | 0.3 | 1.4 | 0.4 | 4.1 | 0.5 | 3.0 | 0.6 | 0.3 | 0.3 | 0.0 | 0.0 | 2.4 | 0.5 | 0.8 | 0.0 | 6.2 | 1.5 | 0.8 | 0.0 | 3.5 | 0.1 | 3.5 | 0.1 | 6.2 | 1.6 |
| **1XGWA** | **176** | **α** | **118** | 1.2 | 1.5 | 0.2 | 2.0 | 0.5 | 0.9 | 0.2 | 0.4 | 0.0 | 0.5 | 0.1 | 0.4 | 0.3 | 0.2 | 0.1 | 1.0 | 0.0 | 1.9 | 0.8 | 0.9 | 0.0 | 0.9 | 0.0 | 0.9 | 0.0 | 0.4 | 0.3 |
| **2CWYA** | **94** | **α** | **8618** | 86.2 | 0.2 | 0.1 | 5.2 | 0.8 | 5.1 | 1.0 | 9.1 | 1.1 | 2.1 | 1.8 | 0.6 | 0.1 | 9.7 | 0.7 | 1.0 | 0.0 | 8.2 | 1.3 | 1.0 | 0.0 | 9.7 | 0.7 | 9.7 | 0.7 | 9.7 | 0.7 |
| **2EJXA** | **139** | **αβ** | **3183** | 31.8 | 1.0 | 0.0 | 1.0 | 0.1 | 0.9 | 0.1 | 0.7 | 0.1 | 1.3 | 0.4 | 0.7 | 0.2 | 1.1 | 0.1 | 1.0 | 0.0 | 1.2 | 0.2 | 1.2 | 0.2 | 1.0 | 0.0 | 1.0 | 0.0 | 1.2 | 0.1 |
| **CR12 > 20% rosetta** | | | | |  |  |  |  |  |  |  |  |  |  |  |  |  |  |  |  |  |  |  |  |  |  |  |  |  |  |
| **1BJ7A** | **156** | **αβ** | **7377** | 73.8 | 0.9 | 0.1 | 0.7 | 0.2 | 1.7 | 0.3 | 0.2 | 0.1 | 1.1 | 0.2 | 1.2 | 0.1 | 2.0 | 0.6 | 0.9 | 0.0 | 3.6 | 1.4 | 4.4 | 1.1 | 4.6 | 1.2 | 4.2 | 1.2 | 2.5 | 0.9 |
| **1BZ4A** | **144** | **α** | **403** | 4.0 | 1.2 | 0.1 | 1.8 | 0.1 | 1.3 | 0.1 | 1.3 | 0.1 | 0.2 | 0.0 | 1.0 | 0.0 | 0.2 | 0.0 | 1.4 | 0.0 | 2.8 | 0.5 | 1.0 | 0.0 | 1.6 | 0.1 | 1.6 | 0.1 | 0.7 | 0.3 |
| **1DUSA** | **194** | **αβ** | **878** | 8.8 | 1.0 | 0.0 | 0.9 | 0.0 | 2.7 | 0.1 | 0.2 | 0.0 | 2.6 | 0.1 | 1.0 | 0.0 | 1.9 | 0.0 | 1.0 | 0.0 | 3.7 | 0.2 | 2.4 | 0.1 | 3.3 | 0.2 | 3.2 | 0.2 | 2.4 | 0.0 |
| **1GAKA** | **141** | **α** | **475** | 4.8 | 1.4 | 0.0 | 2.2 | 0.2 | 1.2 | 0.0 | 2.1 | 0.3 | 0.0 | 0.0 | 1.0 | 0.0 | 0.1 | 0.0 | 1.1 | 0.0 | 4.2 | 0.5 | 1.0 | 0.0 | 2.7 | 0.5 | 0.6 | 0.0 | 0.6 | 0.1 |
| **1GS9A** | **165** | **α** | **151** | 1.5 | 0.9 | 0.0 | 1.5 | 0.1 | 1.0 | 0.2 | 1.3 | 0.0 | 0.3 | 0.1 | 1.0 | 0.0 | 0.6 | 0.3 | 1.3 | 0.1 | 3.3 | 0.6 | 1.0 | 0.0 | 1.2 | 0.3 | 1.4 | 0.1 | 2.2 | 1.1 |
| **1HYPA** | **80** | **α** | **3032** | 30.3 | 1.4 | 0.4 | 1.6 | 0.8 | 1.4 | 0.3 | 1.1 | 0.3 | 0.6 | 0.2 | 1.0 | 0.0 | 2.6 | 0.7 | 1.1 | 0.0 | 1.6 | 0.3 | 1.0 | 0.0 | 0.8 | 0.2 | 0.8 | 0.2 | 3.3 | 1.0 |
| **1ICXA** | **155** | **αβ** | **2542** | 25.4 | 0.9 | 0.1 | 0.8 | 0.1 | 1.4 | 0.1 | 0.2 | 0.0 | 1.3 | 0.0 | 0.8 | 0.0 | 1.6 | 0.2 | 1.0 | 0.0 | 2.5 | 0.4 | 3.6 | 0.4 | 3.8 | 0.4 | 3.6 | 0.3 | 2.2 | 0.3 |
| **1J27A** | **102** | **β** | **309** | 3.1 | 0.8 | 0.1 | 1.0 | 0.0 | 1.5 | 0.1 | 1.2 | 0.1 | 0.7 | 0.1 | 0.9 | 0.0 | 0.7 | 0.1 | 1.0 | 0.0 | 1.1 | 0.1 | 1.3 | 0.1 | 1.6 | 0.1 | 1.8 | 0.0 | 1.0 | 0.1 |
| **1K6KA** | **143** | **α** | **434** | 4.3 | 0.9 | 0.0 | 1.1 | 0.1 | 1.2 | 0.2 | 1.3 | 0.2 | 1.0 | 0.0 | 1.0 | 0.0 | 2.4 | 0.2 | 1.0 | 0.0 | 0.9 | 0.1 | 1.0 | 0.0 | 1.0 | 0.1 | 0.9 | 0.1 | 1.4 | 0.1 |
| **1LWBA** | **122** | **α** | **519** | 5.2 | 0.8 | 0.0 | 1.1 | 0.1 | 1.9 | 0.2 | 0.6 | 0.0 | 1.0 | 0.0 | 1.0 | 0.0 | 1.0 | 0.0 | 1.0 | 0.0 | 1.7 | 0.2 | 1.0 | 0.0 | 0.8 | 0.1 | 0.9 | 0.0 | 1.3 | 0.1 |
| **1M5IA** | **125** | **α** | **1144** | 11.4 | 1.5 | 0.0 | 2.1 | 0.0 | 0.7 | 0.0 | 0.6 | 0.0 | 0.4 | 0.0 | 1.0 | 0.0 | 0.4 | 0.0 | 1.2 | 0.0 | 1.4 | 0.1 | 1.0 | 0.0 | 2.4 | 0.1 | 1.9 | 0.0 | 0.4 | 0.0 |
| **1NFNA** | **191** | **α** | **142** | 1.4 | 0.5 | 0.1 | 0.5 | 0.1 | 1.1 | 0.2 | 0.9 | 0.2 | 0.4 | 0.0 | 1.0 | 0.0 | 0.5 | 0.2 | 1.0 | 0.1 | 1.3 | 0.4 | 1.1 | 0.0 | 1.5 | 0.2 | 1.4 | 0.2 | 0.8 | 0.1 |
| **1OZ9A** | **150** | **αβ** | **476** | 4.8 | 0.8 | 0.1 | 1.5 | 0.0 | 1.7 | 0.2 | 0.4 | 0.1 | 1.1 | 0.0 | 1.0 | 0.0 | 2.0 | 0.2 | 1.0 | 0.0 | 1.9 | 0.1 | 1.4 | 0.1 | 1.9 | 0.1 | 1.6 | 0.0 | 1.5 | 0.2 |
| **1ROAA** | **122** | **β** | **1772** | 17.7 | 0.8 | 0.0 | 1.0 | 0.0 | 1.1 | 0.0 | 0.5 | 0.0 | 1.2 | 0.1 | 0.9 | 0.0 | 0.9 | 0.0 | 1.0 | 0.0 | 1.3 | 0.1 | 2.3 | 0.2 | 2.6 | 0.1 | 2.6 | 0.1 | 1.3 | 0.0 |
| **1TZVA** | **142** | **α** | **1523** | 15.2 | 0.9 | 0.0 | 1.6 | 0.0 | 2.4 | 0.3 | 0.1 | 0.0 | 0.8 | 0.0 | 1.0 | 0.0 | 2.4 | 0.3 | 1.0 | 0.0 | 2.4 | 0.3 | 1.0 | 0.0 | 1.4 | 0.1 | 1.0 | 0.1 | 2.8 | 0.4 |
| **1UBIA** | **76** | **β** | **3422** | 34.2 | 0.8 | 0.1 | 1.5 | 0.1 | 1.7 | 0.2 | 1.2 | 0.1 | 1.0 | 0.1 | 1.0 | 0.0 | 1.9 | 0.3 | 1.0 | 0.0 | 1.9 | 0.2 | 1.4 | 0.2 | 1.3 | 0.2 | 1.0 | 0.0 | 2.3 | 0.4 |
| **1WBAA** | **175** | **β** | **228** | 2.3 | 0.5 | 0.1 | 0.8 | 0.0 | 2.0 | 0.4 | 0.5 | 0.1 | 1.6 | 0.6 | 0.9 | 0.0 | 2.0 | 0.3 | 1.0 | 0.0 | 2.3 | 0.5 | 2.7 | 0.7 | 2.6 | 1.0 | 3.2 | 1.1 | 2.2 | 0.5 |
| **1X91A** | **153** | **α** | **2857** | 28.6 | 0.9 | 0.0 | 1.9 | 0.4 | 1.8 | 0.5 | 1.0 | 0.1 | 0.2 | 0.1 | 1.0 | 0.0 | 2.3 | 0.4 | 1.2 | 0.1 | 5.0 | 1.1 | 1.0 | 0.0 | 2.3 | 0.2 | 1.3 | 0.2 | 4.2 | 1.0 |
| **2CWYA** | **94** | **α** | **4345** | 43.5 | 1.0 | 0.1 | 1.5 | 0.2 | 1.3 | 0.1 | 0.4 | 0.1 | 1.3 | 0.1 | 1.0 | 0.0 | 1.9 | 0.1 | 1.0 | 0.0 | 2.4 | 0.5 | 1.0 | 0.0 | 0.7 | 0.1 | 0.6 | 0.1 | 2.2 | 0.3 |
| **2EJXA** | **139** | **αβ** | **1827** | 18.3 | 0.8 | 0.1 | 0.9 | 0.0 | 1.0 | 0.1 | 0.2 | 0.0 | 0.9 | 0.0 | 1.2 | 0.0 | 1.8 | 0.3 | 1.0 | 0.0 | 1.9 | 0.1 | 2.9 | 0.1 | 3.0 | 0.1 | 2.4 | 0.1 | 2.1 | 0.2 |
| **CR12 > 20% bcl_perturb** | | | | |  |  |  |  |  |  |  |  |  |  |  |  |  |  |  |  |  |  |  |  |  |  |  |  |  |  |
| **1AAJA** | **105** | **β** | **3590** | 29.9 | 1.5 | 0.3 | 1.6 | 0.4 | 3.2 | 0.6 | 1.1 | 0.1 | 3.1 | 0.9 | 1.7 | 0.6 | 1.7 | 0.1 | 1.7 | 0.3 | 2.7 | 0.6 | 3.5 | 0.8 | 0.5 | 0.1 | 0.5 | 0.1 | 3.5 | 0.8 |
| **1BGCA** | **174** | **α** | **737** | 6.1 | 0.5 | 0.2 | 1.0 | 0.1 | 2.7 | 0.6 | 1.0 | 0.0 | 1.6 | 0.2 | 1.1 | 0.0 | 0.8 | 0.0 | 0.9 | 0.0 | 1.8 | 0.1 | 1.0 | 0.0 | 1.3 | 0.1 | 1.3 | 0.1 | 1.6 | 0.1 |
| **1BJ7A** | **156** | **αβ** | **2969** | 24.7 | 1.8 | 0.3 | 1.8 | 0.3 | 3.0 | 0.6 | 0.2 | 0.1 | 4.6 | 1.0 | 2.9 | 0.8 | 0.7 | 0.1 | 1.7 | 0.2 | 2.9 | 0.4 | 4.0 | 0.7 | 1.3 | 0.0 | 1.3 | 0.0 | 2.0 | 0.2 |
| **1BZ4A** | **144** | **α** | **658** | 5.5 | 0.4 | 0.2 | 0.8 | 0.2 | 3.6 | 0.5 | 2.1 | 0.2 | 2.4 | 0.1 | 1.5 | 0.1 | 0.7 | 0.2 | 0.9 | 0.1 | 1.9 | 0.0 | 1.0 | 0.0 | 1.3 | 0.1 | 1.3 | 0.1 | 2.5 | 0.2 |
| **1CHDA** | **203** | **αβ** | **4337** | 36.1 | 2.9 | 0.8 | 3.3 | 1.0 | 2.2 | 0.4 | 0.1 | 0.1 | 4.6 | 1.3 | 2.8 | 0.9 | 0.5 | 0.1 | 2.1 | 0.3 | 4.0 | 1.0 | 4.3 | 0.9 | 1.3 | 0.1 | 1.3 | 0.1 | 1.6 | 0.2 |
| **1DUSA** | **194** | **αβ** | **4351** | 36.3 | 2.8 | 0.7 | 3.0 | 0.8 | 3.7 | 1.0 | 0.6 | 0.2 | 5.2 | 1.4 | 3.3 | 1.2 | 1.4 | 0.1 | 1.5 | 0.1 | 3.5 | 0.9 | 4.7 | 1.0 | 1.3 | 0.0 | 1.3 | 0.0 | 3.8 | 0.8 |
| **1EYHA** | **144** | **α** | **1314** | 11.0 | 1.4 | 0.0 | 1.7 | 0.1 | 3.4 | 0.1 | 0.8 | 0.0 | 4.4 | 0.1 | 2.4 | 0.1 | 1.5 | 0.0 | 1.1 | 0.0 | 1.8 | 0.0 | 1.0 | 0.0 | 1.3 | 0.0 | 1.3 | 0.0 | 2.6 | 0.0 |
| **1G8AA** | **227** | **αβ** | **5822** | 48.5 | 3.3 | 1.2 | 3.5 | 1.3 | 2.7 | 0.7 | 0.2 | 0.2 | 5.5 | 1.7 | 3.7 | 1.5 | 0.4 | 0.2 | 2.1 | 0.4 | 4.6 | 1.6 | 5.0 | 1.4 | 1.3 | 0.1 | 1.1 | 0.0 | 2.8 | 0.3 |
| **1GAKA** | **141** | **α** | **883** | 7.4 | 1.6 | 0.2 | 2.0 | 0.1 | 4.7 | 0.3 | 1.4 | 0.1 | 2.5 | 0.0 | 2.1 | 0.1 | 1.7 | 0.1 | 1.0 | 0.0 | 2.7 | 0.1 | 1.0 | 0.0 | 1.2 | 0.1 | 0.7 | 0.0 | 4.3 | 0.3 |
| **1GCUA** | **295** | **αβ** | **3535** | 29.5 | 3.5 | 0.6 | 3.9 | 0.8 | 4.0 | 1.0 | 0.5 | 0.2 | 5.1 | 1.3 | 4.4 | 1.2 | 0.4 | 0.2 | 2.0 | 0.1 | 3.9 | 0.8 | 4.9 | 0.7 | 1.2 | 0.1 | 1.1 | 0.1 | 3.3 | 0.4 |
| **1GS9A** | **165** | **α** | **775** | 6.5 | 0.7 | 0.3 | 1.0 | 0.3 | 4.1 | 0.4 | 1.0 | 0.1 | 3.1 | 0.1 | 2.0 | 0.2 | 1.4 | 0.1 | 0.9 | 0.1 | 2.9 | 0.2 | 1.0 | 0.0 | 1.2 | 0.1 | 1.2 | 0.1 | 3.0 | 0.3 |
| **1HYPA** | **80** | **α** | **1429** | 11.9 | 0.5 | 0.0 | 1.2 | 0.1 | 2.6 | 0.1 | 0.9 | 0.1 | 2.1 | 0.2 | 1.1 | 0.0 | 1.5 | 0.0 | 0.9 | 0.0 | 1.3 | 0.1 | 1.0 | 0.0 | 0.5 | 0.0 | 0.3 | 0.0 | 1.8 | 0.1 |
| **1IAPA** | **211** | **α** | **1955** | 16.3 | 1.8 | 0.0 | 2.4 | 0.1 | 2.9 | 0.4 | 1.3 | 0.1 | 4.3 | 0.6 | 3.0 | 0.6 | 1.2 | 0.0 | 1.0 | 0.0 | 2.6 | 0.1 | 1.0 | 0.0 | 1.1 | 0.0 | 1.1 | 0.0 | 3.7 | 0.2 |
| **1ICXA** | **155** | **αβ** | **3517** | 29.3 | 2.3 | 0.5 | 2.4 | 0.5 | 2.6 | 0.8 | 1.2 | 0.1 | 4.9 | 1.1 | 2.8 | 0.9 | 1.6 | 0.2 | 2.2 | 0.3 | 3.8 | 0.9 | 4.6 | 0.9 | 1.3 | 0.1 | 1.1 | 0.1 | 4.6 | 1.1 |
| **1IFBA** | **131** | **β** | **4826** | 40.2 | 2.8 | 0.9 | 2.7 | 0.9 | 2.2 | 0.4 | 0.7 | 0.2 | 6.1 | 1.5 | 4.1 | 1.5 | 1.7 | 0.2 | 1.8 | 0.2 | 3.2 | 1.0 | 4.7 | 1.0 | 1.3 | 0.1 | 1.3 | 0.1 | 4.5 | 1.2 |
| **1J27A** | **102** | **β** | **2233** | 18.6 | 1.0 | 0.1 | 1.1 | 0.2 | 2.4 | 0.3 | 0.5 | 0.1 | 1.7 | 0.3 | 0.9 | 0.2 | 0.7 | 0.1 | 0.9 | 0.2 | 3.1 | 0.5 | 3.1 | 0.5 | 1.3 | 0.0 | 1.3 | 0.0 | 2.9 | 0.4 |
| **1JL1A** | **155** | **αβ** | **3495** | 29.1 | 1.5 | 0.4 | 1.6 | 0.4 | 3.9 | 0.9 | 1.3 | 0.1 | 3.6 | 1.1 | 1.5 | 0.5 | 0.2 | 0.1 | 1.0 | 0.1 | 3.1 | 0.8 | 3.5 | 0.7 | 1.1 | 0.0 | 1.0 | 0.0 | 3.1 | 0.8 |
| **1K6KA** | **143** | **α** | **1929** | 16.1 | 1.7 | 0.1 | 2.3 | 0.2 | 2.5 | 0.2 | 1.5 | 0.1 | 3.7 | 0.6 | 2.3 | 0.5 | 1.2 | 0.0 | 1.3 | 0.0 | 2.4 | 0.2 | 1.0 | 0.0 | 1.3 | 0.0 | 1.3 | 0.0 | 3.2 | 0.3 |
| **1LKFA** | **299** | **αβ** | **5612** | 46.8 | 2.8 | 1.0 | 3.1 | 1.1 | 3.9 | 1.2 | 0.6 | 0.2 | 4.4 | 1.5 | 3.9 | 1.4 | 0.6 | 0.2 | 2.2 | 0.7 | 4.6 | 1.5 | 5.9 | 1.7 | 1.1 | 0.0 | 0.8 | 0.1 | 4.5 | 1.5 |
| **1LKIA** | **180** | **α** | **650** | 5.4 | 0.5 | 0.3 | 0.8 | 0.2 | 2.3 | 0.7 | 0.8 | 0.0 | 2.2 | 0.1 | 1.5 | 0.1 | 0.7 | 0.0 | 0.9 | 0.1 | 1.9 | 0.1 | 1.0 | 0.0 | 1.3 | 0.1 | 1.3 | 0.1 | 1.6 | 0.0 |
| **1LWBA** | **122** | **α** | **739** | 6.2 | 0.8 | 0.2 | 0.9 | 0.2 | 2.4 | 0.5 | 1.0 | 0.1 | 3.8 | 0.1 | 2.0 | 0.4 | 1.1 | 0.1 | 1.1 | 0.0 | 2.0 | 0.1 | 1.0 | 0.0 | 1.1 | 0.0 | 0.6 | 0.1 | 2.3 | 0.1 |
| **1M5IA** | **125** | **α** | **593** | 4.9 | 0.2 | 0.0 | 0.6 | 0.0 | 3.6 | 0.2 | 0.8 | 0.0 | 2.8 | 0.1 | 1.2 | 0.1 | 0.5 | 0.1 | 0.8 | 0.0 | 1.6 | 0.1 | 1.0 | 0.0 | 1.4 | 0.1 | 1.4 | 0.1 | 1.3 | 0.0 |
| **1NFNA** | **191** | **α** | **599** | 5.0 | 0.4 | 0.2 | 0.8 | 0.2 | 3.1 | 0.5 | 0.5 | 0.0 | 3.2 | 0.2 | 1.5 | 0.1 | 1.6 | 0.3 | 0.9 | 0.1 | 1.9 | 0.1 | 1.0 | 0.0 | 1.3 | 0.1 | 1.3 | 0.1 | 1.5 | 0.1 |
| **1OA9A** | **214** | **αβ** | **2130** | 17.8 | 1.2 | 0.2 | 1.3 | 0.2 | 2.6 | 0.4 | 1.2 | 0.1 | 2.0 | 0.3 | 0.6 | 0.0 | 0.9 | 0.0 | 1.4 | 0.1 | 2.4 | 0.3 | 3.1 | 0.4 | 1.0 | 0.0 | 0.7 | 0.0 | 3.0 | 0.5 |
| **1OZ9A** | **150** | **αβ** | **3148** | 26.2 | 2.3 | 0.6 | 2.7 | 0.7 | 4.0 | 0.9 | 0.7 | 0.2 | 4.4 | 1.0 | 2.0 | 0.5 | 1.1 | 0.0 | 0.8 | 0.1 | 3.8 | 0.9 | 4.1 | 0.8 | 1.3 | 0.0 | 0.9 | 0.0 | 4.3 | 1.0 |
| **CR12 > 20% bcl_fold** | | | | |  |  |  |  |  |  |  |  |  |  |  |  |  |  |  |  |  |  |  |  |  |  |  |  |  |  |
| **1AAJA** | **105** | **β** | **629** | 6.3 | 0.4 | 0.0 | 1.5 | 0.1 | 2.2 | 0.2 | 0.9 | 0.0 | 0.9 | 0.1 | 1.1 | 0.0 | 1.1 | 0.1 | 1.0 | 0.0 | 1.3 | 0.1 | 0.2 | 0.0 | 1.0 | 0.0 | 1.0 | 0.0 | 1.0 | 0.1 |
| **1BGCA** | **174** | **α** | **1576** | 15.8 | 1.3 | 0.0 | 1.6 | 0.1 | 1.0 | 0.0 | 0.3 | 0.1 | 0.7 | 0.2 | 1.1 | 0.0 | 0.6 | 0.0 | 1.0 | 0.0 | 2.5 | 0.2 | 1.0 | 0.0 | 1.0 | 0.0 | 1.0 | 0.0 | 1.9 | 0.1 |
| **1BJ7A** | **156** | **αβ** | **2030** | 20.3 | 0.9 | 0.0 | 1.3 | 0.0 | 0.9 | 0.1 | 0.3 | 0.1 | 2.3 | 0.2 | 1.7 | 0.1 | 0.8 | 0.1 | 1.0 | 0.0 | 1.1 | 0.1 | 0.7 | 0.0 | 1.0 | 0.0 | 1.0 | 0.0 | 0.6 | 0.0 |
| **1BZ4A** | **144** | **α** | **3240** | 32.4 | 1.0 | 0.0 | 1.3 | 0.1 | 1.4 | 0.2 | 1.4 | 0.2 | 1.1 | 0.2 | 1.1 | 0.1 | 1.1 | 0.2 | 1.0 | 0.0 | 1.2 | 0.1 | 1.0 | 0.0 | 1.0 | 0.0 | 1.0 | 0.0 | 1.4 | 0.2 |
| **1DUSA** | **194** | **αβ** | **229** | 2.3 | 1.0 | 0.1 | 1.7 | 0.3 | 1.4 | 0.1 | 0.7 | 0.1 | 2.2 | 0.3 | 1.3 | 0.3 | 1.1 | 0.1 | 1.0 | 0.0 | 1.8 | 0.3 | 1.2 | 0.7 | 1.1 | 0.0 | 1.1 | 0.0 | 1.5 | 0.5 |
| **1G8AA** | **227** | **αβ** | **290** | 2.9 | 1.1 | 0.0 | 1.1 | 0.1 | 1.2 | 0.1 | 0.2 | 0.0 | 1.9 | 0.2 | 0.9 | 0.1 | 0.9 | 0.0 | 1.0 | 0.0 | 1.7 | 0.3 | 1.4 | 0.5 | 1.0 | 0.0 | 1.0 | 0.0 | 1.1 | 0.1 |
| **1GAKA** | **141** | **α** | **190** | 1.9 | 0.7 | 0.0 | 1.2 | 0.1 | 1.7 | 0.2 | 1.1 | 0.2 | 1.1 | 0.2 | 1.3 | 0.2 | 1.0 | 0.1 | 1.0 | 0.0 | 0.4 | 0.0 | 1.0 | 0.0 | 1.0 | 0.0 | 1.0 | 0.0 | 1.0 | 0.1 |
| **1GS9A** | **165** | **α** | **2032** | 20.3 | 1.3 | 0.1 | 2.2 | 1.0 | 0.0 | 0.0 | 2.7 | 1.4 | 3.7 | 1.2 | 1.3 | 0.4 | 1.0 | 0.0 | 1.0 | 0.0 | 1.2 | 0.2 | 1.0 | 0.0 | 1.0 | 0.0 | 1.0 | 0.0 | 2.1 | 0.8 |
| **1HYPA** | **80** | **α** | **4943** | 49.4 | 1.1 | 0.1 | 1.2 | 0.1 | 0.8 | 0.1 | 0.6 | 0.1 | 1.4 | 0.2 | 1.0 | 0.0 | 0.8 | 0.1 | 1.0 | 0.0 | 1.2 | 0.1 | 1.0 | 0.0 | 1.0 | 0.0 | 1.0 | 0.0 | 1.1 | 0.1 |
| **1ICXA** | **155** | **αβ** | **3338** | 33.4 | 1.1 | 0.1 | 1.0 | 0.1 | 1.4 | 0.1 | 0.8 | 0.2 | 1.8 | 0.2 | 1.2 | 0.1 | 1.2 | 0.1 | 1.0 | 0.0 | 1.2 | 0.1 | 1.6 | 0.2 | 1.0 | 0.0 | 0.9 | 0.0 | 1.6 | 0.1 |
| **1IFBA** | **131** | **β** | **1198** | 12.0 | 1.1 | 0.0 | 0.8 | 0.0 | 0.6 | 0.0 | 1.0 | 0.0 | 1.7 | 0.0 | 1.7 | 0.0 | 0.6 | 0.0 | 1.0 | 0.0 | 0.9 | 0.0 | 1.5 | 0.0 | 1.0 | 0.0 | 1.0 | 0.0 | 0.9 | 0.0 |
| **1J27A** | **102** | **β** | **8662** | 86.6 | 0.9 | 0.2 | 2.0 | 0.8 | 1.1 | 0.4 | 0.4 | 0.6 | 1.4 | 0.4 | 1.1 | 0.4 | 1.5 | 0.5 | 1.0 | 0.0 | 0.2 | 0.2 | 4.7 | 0.5 | 1.0 | 0.0 | 1.0 | 0.0 | 0.7 | 0.7 |
| **1JL1A** | **155** | **αβ** | **220** | 2.2 | 0.7 | 0.1 | 1.1 | 0.0 | 4.0 | 0.9 | 0.9 | 0.1 | 1.5 | 0.1 | 1.4 | 0.2 | 0.8 | 0.1 | 1.0 | 0.0 | 0.6 | 0.2 | 0.6 | 0.1 | 0.9 | 0.0 | 0.9 | 0.0 | 0.3 | 0.1 |
| **1LKFA** | **299** | **αβ** | **642** | 6.4 | 1.1 | 0.0 | 1.4 | 0.0 | 2.5 | 0.3 | 1.2 | 0.0 | 1.6 | 0.1 | 0.9 | 0.0 | 0.7 | 0.0 | 1.1 | 0.0 | 1.6 | 0.0 | 1.5 | 0.1 | 1.1 | 0.0 | 1.0 | 0.0 | 1.0 | 0.0 |
| **1LKIA** | **180** | **α** | **2499** | 25.0 | 1.4 | 0.1 | 1.5 | 0.2 | 0.5 | 0.2 | 1.0 | 0.1 | 0.9 | 0.1 | 1.1 | 0.0 | 0.9 | 0.1 | 1.0 | 0.0 | 2.2 | 0.4 | 1.0 | 0.0 | 1.0 | 0.0 | 1.0 | 0.0 | 2.5 | 0.5 |
| **1LWBA** | **122** | **α** | **404** | 4.0 | 0.7 | 0.0 | 1.2 | 0.2 | 1.0 | 0.1 | 1.7 | 0.3 | 0.8 | 0.2 | 1.5 | 0.2 | 0.8 | 0.3 | 1.0 | 0.0 | 0.1 | 0.0 | 1.0 | 0.0 | 1.1 | 0.1 | 0.7 | 0.5 | 0.4 | 0.1 |
| **1M5IA** | **125** | **α** | **639** | 6.4 | 0.6 | 0.0 | 1.0 | 0.1 | 4.1 | 0.8 | 0.2 | 0.0 | 3.0 | 0.1 | 1.1 | 0.2 | 1.0 | 0.0 | 0.9 | 0.0 | 0.1 | 0.0 | 1.0 | 0.0 | 1.0 | 0.0 | 1.0 | 0.0 | 0.0 | 0.0 |
| **1NFNA** | **191** | **α** | **3336** | 33.4 | 1.0 | 0.0 | 2.2 | 0.3 | 1.9 | 0.3 | 0.2 | 0.2 | 1.3 | 0.2 | 0.8 | 0.1 | 0.9 | 0.1 | 1.0 | 0.0 | 1.0 | 0.2 | 1.0 | 0.0 | 1.0 | 0.0 | 1.0 | 0.0 | 1.2 | 0.3 |
| **1OA9A** | **214** | **αβ** | **265** | 2.7 | 0.8 | 0.1 | 1.2 | 0.1 | 1.3 | 0.2 | 0.9 | 0.1 | 0.4 | 0.0 | 1.0 | 0.1 | 0.7 | 0.2 | 1.0 | 0.0 | 0.9 | 0.1 | 0.9 | 0.3 | 1.0 | 0.0 | 1.0 | 0.0 | 0.5 | 0.2 |
| **1OZ9A** | **150** | **αβ** | **403** | 4.0 | 1.0 | 0.0 | 1.7 | 0.2 | 1.7 | 0.4 | 0.9 | 0.0 | 1.6 | 0.1 | 1.0 | 0.0 | 1.0 | 0.0 | 1.0 | 0.0 | 1.5 | 0.2 | 0.2 | 0.0 | 1.0 | 0.0 | 1.0 | 0.0 | 1.3 | 0.1 |
| **1PRZA** | **252** | **αβ** | **114** | 1.1 | 1.4 | 0.1 | 1.5 | 0.2 | 0.5 | 0.1 | 0.8 | 0.1 | 1.4 | 0.1 | 0.8 | 0.1 | 1.3 | 0.1 | 1.0 | 0.0 | 1.5 | 0.2 | 3.2 | 0.5 | 1.0 | 0.0 | 1.0 | 0.0 | 1.4 | 0.0 |
| **1ROAA** | **122** | **β** | **4637** | 46.4 | 1.2 | 0.1 | 2.2 | 0.5 | 2.0 | 0.3 | 1.3 | 0.5 | 2.2 | 0.7 | 1.3 | 0.2 | 1.1 | 0.7 | 1.0 | 0.0 | 1.7 | 0.2 | 0.8 | 0.3 | 1.0 | 0.0 | 1.0 | 0.0 | 1.6 | 0.4 |
| **1UBIA** | **76** | **β** | **5808** | 58.1 | 0.9 | 0.2 | 1.7 | 0.3 | 1.6 | 0.5 | 4.3 | 1.1 | 4.4 | 0.8 | 1.1 | 0.2 | 1.2 | 0.5 | 1.0 | 0.0 | 0.1 | 0.1 | 0.0 | 0.0 | 1.0 | 0.0 | 1.0 | 0.0 | 4.3 | 1.0 |
| **1VGJA** | **184** | **αβ** | **374** | 3.7 | 1.2 | 0.1 | 1.3 | 0.1 | 1.4 | 0.1 | 0.7 | 0.1 | 1.2 | 0.2 | 1.2 | 0.1 | 0.9 | 0.0 | 1.0 | 0.0 | 1.3 | 0.1 | 1.8 | 0.3 | 1.0 | 0.0 | 1.0 | 0.0 | 1.2 | 0.1 |
| **1WBAA** | **175** | **β** | **1577** | 15.8 | 1.0 | 0.1 | 1.2 | 0.0 | 1.2 | 0.0 | 0.9 | 0.0 | 1.6 | 0.1 | 1.1 | 0.0 | 0.9 | 0.0 | 1.0 | 0.0 | 1.3 | 0.0 | 1.1 | 0.0 | 1.0 | 0.0 | 1.0 | 0.0 | 0.9 | 0.0 |
| **1WNHA** | **225** | **αβ** | **2544** | 25.4 | 1.3 | 0.1 | 1.3 | 0.1 | 0.9 | 0.1 | 0.3 | 0.2 | 3.0 | 0.6 | 1.9 | 0.2 | 1.1 | 0.0 | 1.0 | 0.0 | 1.2 | 0.1 | 1.8 | 0.2 | 1.0 | 0.0 | 1.0 | 0.0 | 1.0 | 0.2 |
| **1WR2A** | **238** | **αβ** | **416** | 4.2 | 1.4 | 0.1 | 0.8 | 0.0 | 0.7 | 0.1 | 0.7 | 0.0 | 1.6 | 0.2 | 1.1 | 0.0 | 1.0 | 0.0 | 1.1 | 0.0 | 1.7 | 0.2 | 3.5 | 0.6 | 1.0 | 0.0 | 1.0 | 0.0 | 1.4 | 0.2 |
| **1WVHA** | **134** | **β** | **2180** | 21.8 | 1.1 | 0.1 | 1.1 | 0.1 | 1.2 | 0.1 | 1.0 | 0.1 | 1.4 | 0.1 | 1.4 | 0.1 | 0.7 | 0.1 | 1.0 | 0.0 | 1.1 | 0.1 | 1.2 | 0.1 | 1.0 | 0.0 | 1.0 | 0.0 | 1.3 | 0.1 |
| **1X91A** | **153** | **α** | **2411** | 24.1 | 1.0 | 0.1 | 1.1 | 0.1 | 1.7 | 0.1 | 1.0 | 0.1 | 1.4 | 0.3 | 0.4 | 0.1 | 1.4 | 0.0 | 1.0 | 0.0 | 2.7 | 0.6 | 1.0 | 0.0 | 1.1 | 0.0 | 1.1 | 0.0 | 2.7 | 0.7 |
| **1XGWA** | **176** | **α** | **575** | 5.8 | 1.1 | 0.1 | 1.6 | 0.1 | 1.1 | 0.1 | 0.5 | 0.0 | 0.7 | 0.1 | 0.6 | 0.1 | 0.4 | 0.1 | 1.0 | 0.0 | 1.7 | 0.2 | 1.0 | 0.0 | 1.0 | 0.0 | 1.0 | 0.0 | 0.6 | 0.0 |
| **2CWYA** | **94** | **α** | **1705** | 17.1 | 0.3 | 0.0 | 1.4 | 0.2 | 2.1 | 0.3 | 0.3 | 0.1 | 5.2 | 0.3 | 1.1 | 0.0 | 1.2 | 0.0 | 1.0 | 0.0 | 0.9 | 0.1 | 1.0 | 0.0 | 1.2 | 0.0 | 1.2 | 0.0 | 0.9 | 0.1 |
| **2E3SA** | **255** | **αβ** | **2606** | 26.1 | 1.4 | 0.1 | 1.5 | 0.1 | 1.4 | 0.1 | 0.2 | 0.0 | 1.7 | 0.0 | 1.1 | 0.1 | 0.7 | 0.0 | 1.1 | 0.0 | 1.5 | 0.1 | 2.6 | 0.1 | 1.0 | 0.0 | 1.0 | 0.0 | 0.8 | 0.1 |
| **2EJXA** | **139** | **αβ** | **1510** | 15.1 | 1.1 | 0.0 | 1.5 | 0.0 | 1.4 | 0.1 | 1.0 | 0.0 | 2.7 | 0.3 | 1.9 | 0.2 | 1.2 | 0.0 | 1.0 | 0.0 | 1.0 | 0.0 | 0.7 | 0.1 | 1.0 | 0.0 | 1.0 | 0.0 | 1.3 | 0.0 |
| **2OPWA** | **291** | **αβ** | **493** | 4.9 | 1.3 | 0.1 | 1.1 | 0.0 | 1.4 | 0.1 | 1.1 | 0.1 | 0.8 | 0.0 | 1.0 | 0.0 | 0.9 | 0.1 | 1.1 | 0.0 | 1.5 | 0.0 | 1.7 | 0.2 | 1.0 | 0.0 | 1.0 | 0.0 | 0.9 | 0.0 |
| **2YV8A** | **164** | **β** | **1959** | 19.6 | 0.8 | 0.1 | 1.4 | 0.1 | 1.5 | 0.0 | 1.2 | 0.0 | 1.1 | 0.1 | 1.0 | 0.1 | 0.7 | 0.1 | 1.0 | 0.0 | 1.5 | 0.1 | 1.1 | 0.1 | 1.0 | 0.0 | 1.0 | 0.0 | 1.0 | 0.1 |

Table S1 Cross validated average enrichments for individual protein models sets and different quality criteria

The table lists decoy sets that had at least 1% and at most 99% native-like models according to the quality criteria (RMSD100 < 8Å, GDT_TS > 25%, CR12 > 20%). Ten cross validation sets of the protein models with 10% native-like and 90% non-native like structures have been assembled, the enrichment according to the potential was calculated, and averaged over the ten cross validation sets. The standard deviations are reported as well. Table 1 was derived from these numbers.
