# Supplementary material for: BCL::Score—Knowledge Based Energy Potentials for Ranking Protein Models Represented by Idealized Secondary Structure Elements
Source: PLoS One. 2012 Nov 16;7(11):e49242. doi: 10.1371/journal.pone.0049242 (PMC3500277; doi:10.1371/journal.pone.0049242)
Supplement: Appendix S1 — BCL::Score availability and its usage. (DOCX) [file pone.0049242.s007.docx]

Appendix S1 BCL::Score availability and its usage.

BCL::Score is available as a binary for download under <http://www.meilerlab.org>

An individual pdb file or a set of proteins can be scored at once, with all scoring function introduced in this manuscript. Additionally, if a template structure is given, protein similarity measures can be calculated as well. The output is a table, with one row for each protein, and columns for all scores and quality measures.

If a template pdb is given, the terminal output contains the rank of the template structure for all of the scores. It is also possible to give any quality measure and a cutoff to calculate the enrichment for models below that threshold. This gives an indicator for how well the individual potential discriminates for native like protein structures in a set of models.

Command line scoring single pdb:

bcl.exe ScoreProtein -pdb model.pdb -score_table_write scores.table -template template.pdb -quality RMSD GDT_TS -atoms CA -convert_to_natural_aa_type -sspred JUFO PSIPRED

Input files: model.pdb protein to score; implicitly -sspred JUFO and PSIPRED requires also model.jufo and model.psipred_ss2

template.pdb template reference for quality calculation

Output files: scores.table

Command line scoring multiple pdbs:

bcl.exe ScoreProtein -pdblist pdbs.ls -score_table_write scores.table -template template.pdb -quality RMSD GDT_TS -atoms CA -convert_to_natural_aa_type -sspred JUFO PSIPRED

Input files: pdbs.ls a list of pdb files names; implicitly -sspred JUFO and PSIPRED requires also *.jufo and *.psipred_ss2 for each pdb file

template.pdb template reference for quality calculation

Output files: scores.table

Commandline enrichment:

bcl.exe ScoreProtein -score_table_read scores.table -rank template.pdb -weight_set assembly.scoreweights -sspred JUFO PSIPRED -enrichment 0.1 8.0 10 RMSD100 less

Input files: scores.table; assembly.scoreweights

Output files: none, all the enrichment and ranks are written to the terminal

The enrichment is calculated by balancing the set of scores models, so that the resulting table has a fraction of 0.1 with RMSD100 less than 8Å. 10 different tables are generated with different subsets from the input scores.table. The assembly.scoreweights is used to calculate the sum – the weighted consensus score from each of the scoring terms.
